# Assessment of genotyping array performance for genome-wide association studies and imputation in African cattle

**DOI:** 10.1186/s12711-022-00751-5

**Published:** 2022-09-04

**Authors:** Valentina Riggio, Abdulfatai Tijjani, Rebecca Callaby, Andrea Talenti, David Wragg, Emmanuel T. Obishakin, Chukwunonso Ezeasor, Frans Jongejan, Ndudim I. Ogo, Fred Aboagye-Antwi, Alassane Toure, Jahashi Nzalawahej, Boubacar Diallo, Ayao Missohou, Adrien M. G. Belem, Appolinaire Djikeng, Nick Juleff, Josephus Fourie, Michel Labuschagne, Maxime Madder, Karen Marshall, James G. D. Prendergast, Liam J. Morrison

**Affiliations:** 1 The Roslin Institute and Royal (Dick) School of Veterinary Studies, University of Edinburgh, Midlothian, EH25 9RG UK; 2 Centre for Tropical Livestock Genetics and Health (CTLGH), Roslin Institute, University of Edinburgh, Easter Bush Campus, Midlothian, EH25 9RG UK; 3 Centre for Tropical Livestock Genetics and Health (CTLGH), ILRI Ethiopia, P.O Box 5689, Addis Ababa, Ethiopia; 4 Biotechnology Division, National Veterinary Research Institute, Vom, Plateau State Nigeria; 5 Biomedical Research Centre, Ghent University Global Campus, Songdo, Incheon, South Korea; 6 Department of Veterinary Pathology and Microbiology, University of Nigeria, Nsukka, Enugu State Nigeria; 7 Department of Veterinary Tropical Diseases, Faculty of Veterinary Science, University of Pretoria, Onderstepoort, South Africa; 8 National Veterinary Research Institute, Vom, Nigeria; 9 Department of Animal Biology and Conservation Sciences, University of Ghana, Accra, Ghana; 10 Laboratoire National d’Appui Au Dévéloppement Agricole(LANADA)/Laboratoire Central Vétérinaire de Bingerville, Bp: 206, Bingerville, Côte d’Ivoire; 11 Department of Microbiology, Parasitology and Biotechnology, Sokoine University of Agriculture, Morogoro, Tanzania; 12 Central Vétérinaire de Diagnostic (LCVD), Conakry, Guinea; 13 Ecole Inter-Etats des Sciences et Médecine Vétérinaires (EISMV) de Dakar, Dakar, Senegal; 14 Université Polytechnique de Bobo-Dioulasso (UPB), Bobo -Dioulasso, Burkina Faso; 15 Bill & Melinda Gates Foundation, Seattle, WA USA; 16 Clinvet, 1479 Talmadge Hill South, Waverly, NY 14892 USA; 17 Clinomics, Uitzich Road, Bainsvlei, Bloemfontein, 9338 South Africa; 18 Clinvet, Uitzich Road, Bainsvlei, Bloemfontein, 9338 South Africa; 19 Clinglobal, B03/04, The Tamarin Commercial Hub, Jacaranda Avenue, Tamarin, 90903 Mauritius; 20 Centre for Tropical Livestock Genetics and Health (CTLGH), ILRI Kenya, P.O. Box 30709, Nairobi, 00100 Kenya; 21 International Livestock Research Institute, P.O. Box 30709, Nairobi, 00100 Kenya

## Abstract

**Background:**

In cattle, genome-wide association studies (GWAS) have largely focused on European or Asian breeds, using genotyping arrays that were primarily designed for European cattle. Because there is growing interest in performing GWAS in African breeds, we have assessed the performance of 23 commercial bovine genotyping arrays for capturing the diversity across African breeds and performing imputation. We used 409 whole-genome sequences (WGS) spanning global cattle breeds, and a real cohort of 2481 individuals (including African breeds) that were genotyped with the Illumina high-density (HD) array and the GeneSeek bovine 50 k array.

**Results:**

We found that commercially available arrays were not effective in capturing variants that segregate among African indicine animals. Only 6% of these variants in high linkage disequilibrium (LD) (r^2^ > 0.8) were on the best performing arrays, which contrasts with the 17% and 25% in African and European taurine cattle, respectively. However, imputation from available HD arrays can successfully capture most variants (accuracies up to 0.93), mainly when using a global, not continent-specific, reference panel, which partially reflects the unusually high levels of admixture on the continent. When considering functional variants, the GGPF250 array performed best for tagging WGS variants and imputation. Finally, we show that imputation from low-density arrays can perform almost as well as HD arrays, if a two-stage imputation approach is adopted, i.e. first imputing to HD and then to WGS, which can potentially reduce the costs of GWAS.

**Conclusions:**

Our results show that the choice of an array should be based on a balance between the objective of the study and the breed/population considered, with the HD and BOS1 arrays being the best choice for both taurine and indicine breeds when performing GWAS, and the GGPF250 being preferable for fine-mapping studies. Moreover, our results suggest that there is no advantage to using the indicus-specific arrays for indicus breeds, regardless of the objective. Finally, we show that using a reference panel that better represents global bovine diversity improves imputation accuracy, particularly for non-European taurine populations.

**Supplementary Information:**

The online version contains supplementary material available at 10.1186/s12711-022-00751-5.

## Background

The African continent is home to many livestock breeds that are adapted to their local environments across diverse agro-ecological zones, with a diversity that has been shaped by a delicate balance between human and environmental selection [1]. Livestock, particularly cattle, are central to the African society and economy. They are sources of food and generate income through meat, milk, and hide. They also provide draft power and manure in crop production, are a means of transportation, are used in festivals and traditional ceremonies (marriage, birth, death, coronation, and initiation ceremonies) and are a source of pride, prestige, and status [1]. However, in spite of these benefits, livestock productivity in Africa is currently less than optimal. Hence, developing genetic improvement programmes to increase the productivity of African cattle breeds is essential to ensure that they can match and fulfil demand and population growth.

In a study that aimed at the genome characterisation of five indigenous African cattle breeds, Kim et al. [2] highlighted and mapped several unique African adaptation-related traits, representing responses to climatic challenges, disease resistance, and artificial selection. Thus, these results provided genomic evidence and options for implementing genetic strategies to improve cattle productivity and resilience in Africa [2]. A key step to understand African livestock production is to map the genetic loci that underlie important traits and phenotypes. Traditionally, this is most commonly achieved through the use of genome-wide association studies (GWAS) that have successfully identified hundreds of single-nucleotide polymorphisms (SNPs) associated with complex traits in cattle. However, these studies have focused mainly on cattle that derive from European or Asian breeds. Furthermore, the existing genotyping arrays that are used in GWAS cover only a limited repertoire of sequence variation, which is often biased towards variants that are common to the European breeds used in the SNP discovery step of the development of arrays.

In cattle, the densities of commercially available SNP genotyping arrays range from ~ 3000 up to ~ 777,000 variants. The first array used was the BovineSNP50 Genotyping (now V3) BeadChip (https://www.illumina.com/Documents/products/datasheets/datasheet_bovine_snp5O.pdf; SNP50V3) soon after followed by the Illumina HD (https://www.illumina.com/documents/products/datasheets/datasheet_bovineHD.pdf) and the Axiom Genome-Wide BOS1 array (https://www.thermofisher.com/order/catalog/product/901791#/901791). Several other arrays have been subsequently developed for more specific purposes. Some arrays that were intended to be used for genomic selection were designed with a lower density of variants, such as the Illumina Golden Gate Bovine3K Genotyping Beadchip (BOVGGPV3K; http://www.illumina.com/Documents/products/datasheets/datasheet_bovine3k.pdf); the BovineLD Genotyping BeadChip (BOVLDC and BOVLDV2A; https://www.illumina.com/Documents/products/datasheets/datasheet_bovineLD.pdf); and the GeneSeek Genomic Profiler (GGP) low-density BeadChip for Dairy Cattle (GGPLDV1; GGPLDV3; and GGPLDV4). Other arrays have been developed for more specific applications, for example, the GGPF250 array that contains 34,000 common variants present on many of the genotyping assays currently used by the cattle industry and 199,000 predicted functional variants [3]; the GGP BeadChip GGPHDV3 (https://www.neogen.com/en-gb/categories/genotyping-arrays/ggp-bovine-150k/) that uses the most informative SNPs from the three Illumina chips, plus comprehensive parentage, disease and trait relevant SNPs; the IDBV3 array (http://www.icbf.com/?page_id=2170) that contains diagnostic probes for genetic diseases and traits; and the GGP Bovine 50K for Dairy (BOVG50V1) array, which comprises SNPs that were selected for maximum informativeness in the Holstein, Jersey, Brown Swiss, Ayrshire, Guernsey, Gyr, and Girolando dairy breeds. Although indicine breeds have been used in the design of many of these arrays, they tend to focus on providing a more uniform genome coverage for a majority of the taurine breeds. However, two arrays have been designed and used specifically for indicine breeds: the GGP Bos indicus HD array (IND90KH) and the GGP indicus array (GGPIND35; https://www.neogen.com/globalassets/pim/assets/original/10000/official_ggp-indicus_brochure.pdf). Therefore, in view of this wide range of available arrays, it is difficult to predict which one would be optimal and suitable for African cattle (which are often a mixture of taurine and indicine breeds).

Although assessment of genome-wide genetic variation is now possible using next-generation sequencing technologies, it remains prohibitively expensive to whole-genome sequence large cohorts of animals [4]. Consequently, genotyping arrays remain the most commonly used tool in GWAS, but how well these different arrays capture the genetic variation of African breeds and work in GWAS of such breeds is unclear.

Imputation is often used to improve coverage in GWAS, where untyped variants are inferred by combining partial haplotypes found in a study sample (i.e., a target set) using genotyping arrays, with the full haplotypes available in a more densely characterised reference set or panel (for a review see [5]). Highly accurate genotype imputation methods have been developed to predict large numbers of genetic variants from a much smaller subset of known genotypes [6]. In humans, it has been reported that imputation works very well for common variants but comparatively much less well at lower minor allele frequencies [7]. Performing imputation to the whole-genome level requires a high-density, high-quality reference panel and has been successfully achieved in several species including cattle [8, 9]. However, imputation performance has been little explored in African cattle breeds. Since the design of the bovine genotyping arrays has primarily been focused on European taurine cattle breeds, they may perform less well on African cattle, which are composed of a mixture of African taurine and indicine backgrounds. Since these lineages have arisen from two different ancestral Auroch (*Bos primigenius*) populations for which the last common ancestor is estimated to have lived at least 210,000 years ago [10, 11], they display substantial genetic divergence that is poorly represented by the current arrays. Furthermore, only a few suitable reference haplotypes are available, which hampers the use of imputation in African cattle breeds and means that imputation has relied on haplotypes from other less suitable, primarily European taurine, breeds.

The objective of this study was to determine the best strategy for performing GWAS in African cattle. We assessed which of the currently available bovine arrays best capture the diversity across African breeds, and which are the most effective for performing genome-wide imputation. We compared imputation performance using different reference panels and evaluated which approaches and arrays best capture putatively functional variants. Finally, we demonstrate the utility of these approaches on a real cohort of 2481 genotyped African cattle samples.

## Methods

### Evaluation of the performance of bovine genotyping arrays for African cattle

This analysis was undertaken to assess the relative merits of using the currently available bovine genotyping arrays on African cattle breeds. Thus, we evaluated the performance of 23 commercial bovine genotyping arrays with densities ranging from ~ 3000 to ~ 777,000 SNPs on a European taurine breed (Holstein–Friesian) and two indigenous African cattle breeds, West African taurine (NDama) and East African indicine Zebu (Boran). Table 1 provides the list of the arrays tested and the number of variants on each array. It should be mentioned that the names used in this paper for the arrays are not the official designation; instead, for consistency with previous studies and ease of cross-reference, we use the nomenclature used for the arrays on the NGARP Data Repository (https://www.animalgenome.org/repository/cattle/UMC_bovine_coordinates).

**Table 1:** Arrays used in this study, numbers of variants per array, number of retained variants from the WGS data, before and after quality control (QC), whether imputation was successful or not

Array	Number of variants on the array	Number of variants retained from WGS before QC	Number of variants retained from WGS after QC	Imputation successful or not
HD	777,962	685,468 (88%)	409,013 (53%)	Yes
BOS1	648,875	595,938 (92%)	257,217 (40%)	Yes
GGPF250	227,234	137,609 (61%)	51,803 (23%)	Yes
GGPHDV3	139,977	125,712 (90%)	72,456 (52%)	Yes
GGP90KT	76,999	70,603 (92%)	40,749 (53%)	Yes
IND90KH	74,150	66,879 (90%)	42,484 (57%)	Yes
ZMD2	68,213	53,314 (78%)	25,223 (37%)	Yes
ZOETIS1	59,825	45,559 (76%)	22,657 (38%)	Yes
BOVMD	57,134	43,860 (77%)	20,151 (35%)	Yes
IDBV3	53,450	44,447 (83%)	20,711 (39%)	Yes
SNP50V3	53,218	47,788 (90%)	21,341 (40%)	Yes
ANGGS	49,541	44,082 (89%)	21,957 (44%)	Yes
BOVG50V1	47,844	41,214 (86%)	25,304 (53%)	Yes
GGPIND35	35,339	31,903 (90%)	22,253 (63%)	Yes
GGPLDV4	30,105	26,330 (87%)	16,072 (53%)	Yes
GGPLDV3	26,504	23,035 (87%)	14,216 (54%)	Yes
ZLD4	20,503	17,378 (85%)	9358 (46%)	No
ZLD2	18,206	15,769 (87%)	8649 (47%)	No
GGPLDV1	8762	6265 (71%)	3645 (42%)	No
BOVLDV2A	7931	7163 (90%)	4234 (53%)	No
BOVLDC	6909	6305 (91%)	3670 (53%)	No
DAIRYULDB	4227	3651 (86%)	2276 (54%)	No
BOVGGPV3K	2900	2603 (90%)	1720 (59%)	No

Values in brackets are percentages relative to the overall number of variants on each array

We collated 120 Illumina whole-genome sequences (WGS) from 40 individuals of each of the three bovine breeds. The NDama sequences included samples from Guinea (n = 21), Nigeria (n = 10) and Senegal (n = 9); and the Boran samples from Ethiopia (n = 30) and Kenya (n = 10). The sequences of the 40 NDama and 20 Ethiopian Boran samples were recently generated as part of the Genomic Reference Resource for African Cattle (GRRFAC) Initiative (https://grrfac.ilri.org), while the remaining 20 African cattle and 40 Holstein–Friesian sequences were retrieved from public databases, for more details on the samples and project accession numbers (see Additional file 1: Tables S1 and S2).

Sequencing reads were aligned to the cattle reference ARS-UCD1.2 genome [12], using the BWA-mem version 0.1.17 software [13], and the GATK best practice recommendations were followed to call SNPs. The identified SNPs were subjected to the GATK’s variant quality score recalibration (VQSR) approach, following the steps specified in [14], using multiple sources of predefined high-quality SNPs. These include the BQSR file from the 1000 Bulls genome project [15], 24 SNP chip datasets, and known variants from Ensembl v95 (ftp://ftp.ensembl.org/pub/release-95/variation/vcf/bos_taurus/). After VQSR, we retained the SNPs in the 99% tranche. Additional file 2: Figure S1 shows the tranche-specific true positives (TP), false positives (FP) and the cumulative TP, as well as the ratio of transition (Ti) to transversion (Tv) SNPs (i.e., Ti/Tv ratio), generated using VQSR. Furthermore, we used the BCFtools version 1.11 [16] to select biallelic SNPs with a QUAL score > 100 and excluded the variants with a high proportion (> 25%) of missingness in each cattle breed, thus approximately 5500 variants were further excluded from the final SNP call.

For each cattle breed, we used the VCFtools version 0.1.15 package [17] to select the WGS SNPs that overlap with the positions of the SNPs in each of the 23 available bovine genotyping arrays. Subsequently, we used the PLINK software [18] v1.90b4 64-bit (www.cog-genomics.org/plink/1.9/) to estimate the pairwise linkage disequilibrium (LD, r^2^) correlation between the WGS variants and the array SNPs, which was calculated across 500-kb genome windows. The WGS SNPs that were highly correlated (r^2^ > 0.8) with those on each array were identified and used for further analyses. PLINK was also used to estimate the allele frequencies of the SNPs, and the LD decay was compared in the three cattle breeds.

### Imputation analysis

#### Reference genotype data

##### Reference panel 1—whole-genome sequence data

In addition to the 120 WGS for the three breeds of interest, Illumina WGS data for 427 other bovine genomes representing a wide diversity of global cattle breeds were collated to act as an imputation reference panel. These were aligned to the cattle reference ARS-UCD1.2 genome [12]. More details on how the WGS data were processed are in Dutta et al. [14]. For quality control (QC), we applied the following criteria: individuals with a high proportion (> 25%) of missing genotypes were again removed, as well as all highly related individuals (relatedness value from vcftools -relatedness2 > 0.0625, [17]) to minimise biases in the downstream analyses, and variants with a call rate (CR) ≥ 75% and genotype quality (GQ) ≥ 25 were retained. The final WGS reference panel, referred to as the Global Reference Panel, comprised 289 distinct individuals, which spanned a diverse range of breeds and geographic locations (55 populations, among which 13 European, 12 African, 28 Asian, and 2 Middle Eastern) (see Additional file 1: Table S1 and Additional file 3: Figure S2). We also evaluated imputation accuracy using three subset reference panels that were derived from the global 289 sample set, which hereafter we refer to as the African Reference Panel (87 individuals), the Asian Reference Panel (106 individuals) and the European Reference Panel (77 individuals), to test the effect of using a mixed reference panel compared to using continent-specific panels.

##### Reference panel 2—Illumina bovine HD array

In the two-step imputation analysis [19, 20] from 50 k to WGS (50 k > HD > WGS), a slightly different QC was applied for the HD data used as the intermediate imputation reference panel. The same criteria were applied for minor allele frequency (MAF) as well as SNP and individual missingness, as described in the “Target genotype data” section. Furthermore, individuals represented in the 50 k data were removed from the HD reference panel, as well as the highly related individuals (relatedness value from vcftools -relatedness2 > 0.0625, [17]).

#### Target genotype data

##### Subsets of the WGS data

For the masked analysis, target sets were created by retaining the WGS genotypes that overlapped with the variants of each of the 23 available bovine genotyping arrays from the Global Reference Panel as well as its three African (n = 87), Asian (n = 106) and European (n = 77) subsets. These target sets were then used to impute to WGS level, using the Global Reference Panel. Table 1 shows the number of variants on each array and how many were retained from the WGS data before and after QC (65,107,956 and 10,282,187 variants, respectively), and also whether the imputation was successful or not.

##### Illumina bovine HD array data

In total, 3092 cattle from four African countries (Tanzania, Ghana, Nigeria, and Burkina Faso) were genotyped using the Illumina bovine HD array (777,962 SNPs). These 3092 cattle were combined with other available samples genotyped with the Illumina HD array producing a final set of 3852 samples. For more details on the number of animals genotyped with the Illumina HD array per breed/population from each data source (see Additional file 4: Table S3). After lifting-over the positions to the cattle reference ARS-UCD1.2 [12] and fixing allele strand inconsistencies (fully described in the relevant section), 718,874 genotypes were retained [(see Additional file 5: Table S4) for the coordinates of the variants retained after liftover on both the UMD3.1 and ARS-UCD1.2 assemblies].

The PLINK software [18] v1.90b4 64-bit (www.cog-genomics.org/plink/1.9/) was used to remove: (i) SNPs located on the sex chromosomes, or the mitochondrial genome or without position information on the ARS-UCD1.2 genome; (ii) SNPs with a call rate lower than 0.90; (iii) SNPs with a MAF lower than 0.01; and (iv) individuals with more than 10% missing genotypes. Furthermore, highly related individuals (i.e., duplicated genotypes of the same individuals; relatedness value from vcftools -relatedness2 > 0.40, [17]) were also removed. After QC, 2481 samples (among which 1740 were from four African countries, Tanzania, Ghana, Nigeria, and Burkina Faso) and 577,345 variants were retained.

##### GeneSeek bovine 50 k array (BOVG50V1) data

From the 3092 cattle sampled from the four African countries and genotyped with the Illumina HD array, 668 were also genotyped using the GeneSeek (a Neogen Company, Lincoln, NE, USA) bovine 50 k array (47,844 SNPs). The same QC criteria as for the HD data were applied. After QC, 602 samples and 37,118 variants were retained.

##### LiftOver and strand fixing

Since the coordinates of the array variant data were originally mapped to the bovine UMD3.1 genome assembly (http://www.cbcb.umd.edu/production_assemblies), it was necessary to lift them over to the ARS-UCD1.2 bovine genome assembly [12], making sure to account for any strand switches. Bovine HD genotype data in the TOP coding format were converted to the FOR coding format using the iConvert.py tool from SNPchiMp v.3 (https://webserver.ibba.cnr.it/SNPchimp/; https://github.com/nicolazzie/SNPchimpRepo). Map positions were updated from UMD3.1 (http://www.cbcb.umd.edu/production_assemblies) to the cattle reference ARS-UCD1.2 genome [12] for the Bovine HD array map, by cross-referencing SNP ID with those in a file of updated Bovine HD array coordinates that is available online at the NGARP Data Repository (https://www.animalgenome.org/repository/cattle/UMC_bovine_coordinates). Bovine HD array data were processed in PLINK [18] v1.90b4 64-bit (www.cog-genomics.org/plink/1.9/) using the updated map file to retain only autosomal SNPs and to set the reference allele (-a1-allele) for each SNP to match a VCF file of Bovine HD array SNPs extracted from WGS data aligned to ARS-UCD1.2 [12]. To address any remaining allele inconsistencies between the datasets that were not resolved by the iConvert and PLINK reference allele setting steps above, we partitioned each dataset by chromosome and applied two custom scripts. The first script, written in R (http://www.r-project.org), reads as inputs a VCF file and a CSV file of the SNPchiMp v.3 data for the Bovine HD array and its allele coding formats (A/B forward and A/B top) from the native platform. The script identifies SNPs with missing REF and/or ALT allele annotations in the VCF file, cross-references the CSV file for the appropriate alleles according to its coding format, and updates the missing values. The second script, written in Python (Python Software Foundation, https://www.python.org/), reads as inputs a query VCF file and a reference VCF file, and uses the latter to re-annotate the former. Briefly, it identifies different types of allele inconsistencies (REF and ALT in reverse order, REF and/or ALT strand mismatch, allele ambiguity AT and CG) and updates those that can be corrected faithfully, and discards those that cannot (due to ambiguity, or absent in the reference VCF, or non-biallelic). The fixed chromosomal VCF files were concatenated and the resulting VCF files for each dataset were merged using bcftools v1.3 [16].

##### Imputation approaches

Imputation performance was tested in two scenarios:

1. a masked analysis was carried out using the four target sets (i.e. Global, African, Asian, and European) that were created by retaining only the WGS genotypes that overlapped with the variants of each available bovine genotyping array. These target sets were then used to impute to WGS level, using the Global Reference Panel. This analysis allowed us to evaluate which array would, in general, perform better and whether some arrays might be better than others in some subsets, depending on how they were designed.
2. Using the 50 k and HD genotype data directly imputed to WGS as well as the two-step imputation from 50 k to WGS (50 k > HD > WGS), both using the Global Reference Panel and the subsets (i.e., African, Asian and European Reference Panels), to evaluate whether imputation worked better when using a mixed Reference Panel or a continent-specific panel.

##### Imputation analysis and estimation of imputation accuracy

The reference panels were pre-phased using BEAGLE v5.0 [21], whereas phasing of the target sets was tested with both BEAGLE v5.0 [21] and SHAPEIT4 [22], using one genotyping array (BOVMD). Since the imputation accuracies were similar [(see Additional file 6: Figure S3), for an example of the comparison of the imputation accuracies when phasing was done with either BEAGLE or SHAPEIT4], BEAGLE-phased data were used in all subsequent analyses.

Imputation was performed with Minimac4 (https://genome.sph.umich.edu/wiki/Minimac4), which uses an ad-hoc method to estimate imputation accuracy at genotyped sites in the target set. For each genotyped site, Minimac4 hides all known genotypes for that site and calculates an imputed dosage (in addition to the usual alternate allele dosage that is calculated by assuming the genotypes are known at the site). This special imputed value is called Leave-One-Out dosage (LooDosage) and is only available for genotyped sites and is used to calculate empirical R^2^ (ER^2^) which is the correlation between the true genotyped values and the imputed dosages that were obtained by hiding all known genotypes for the given variant.

In the masked analyses, imputed genotypes were compared to those called in the WGS data (except for the variants that overlapped with the bovine genotyping arrays) by calculating genotype concordance (i.e., dosage R^2^) using bcftools stats [16].

Imputation accuracy was evaluated by leave-one-out cross-validation using 100 of the 289 animals from the WGS data as the target set. In this procedure, one individual was removed from the reference panel and its genotypes were imputed using the remaining animals as the reference panel. This was repeated for each of the 100 animals, randomly selected from the WGS data.

#### Annotated variants

Variants in both sets of WGS data were annotated using the Ensembl Variant Effect Prediction (VEP) v95 tool [23] configured to define the impact of variants (LOW, MODERATE and HIGH) according to their locations. Imputation accuracy (i.e., dosage R^2^) was calculated using bcftools stats [16] for each of the three classes (LOW, MODERATE and HIGH) using the target sets from the masked analysis with the Global Reference Panel.

## Results

To determine the best strategy for performing GWAS in African cattle, we compiled two large datasets of genotyping data. The first dataset was a collection of 409 WGS spanning the three lineages (European taurine, African taurine and indicine) from which global cattle breeds derive, and that included 40 genomes each of the NDama, Boran and Holstein–Friesian breeds. The second dataset included 1740 animals genotyped with the Illumina HD array and originating from four African countries (Tanzania, Ghana, Nigeria, and Burkina Faso). For comparison purposes, these data were combined with other available samples that spanned global breeds and genotyped with the same Illumina HD array, for a combined total of 2481 samples. Among the 1740 cattle sampled from the four African countries and genotyped with the Illumina HD array, 602 samples genotyped with the GeneSeek bovine 50 k array were also available.

### The commercial arrays tagged indicine cattle poorly

**Fig. 1:**
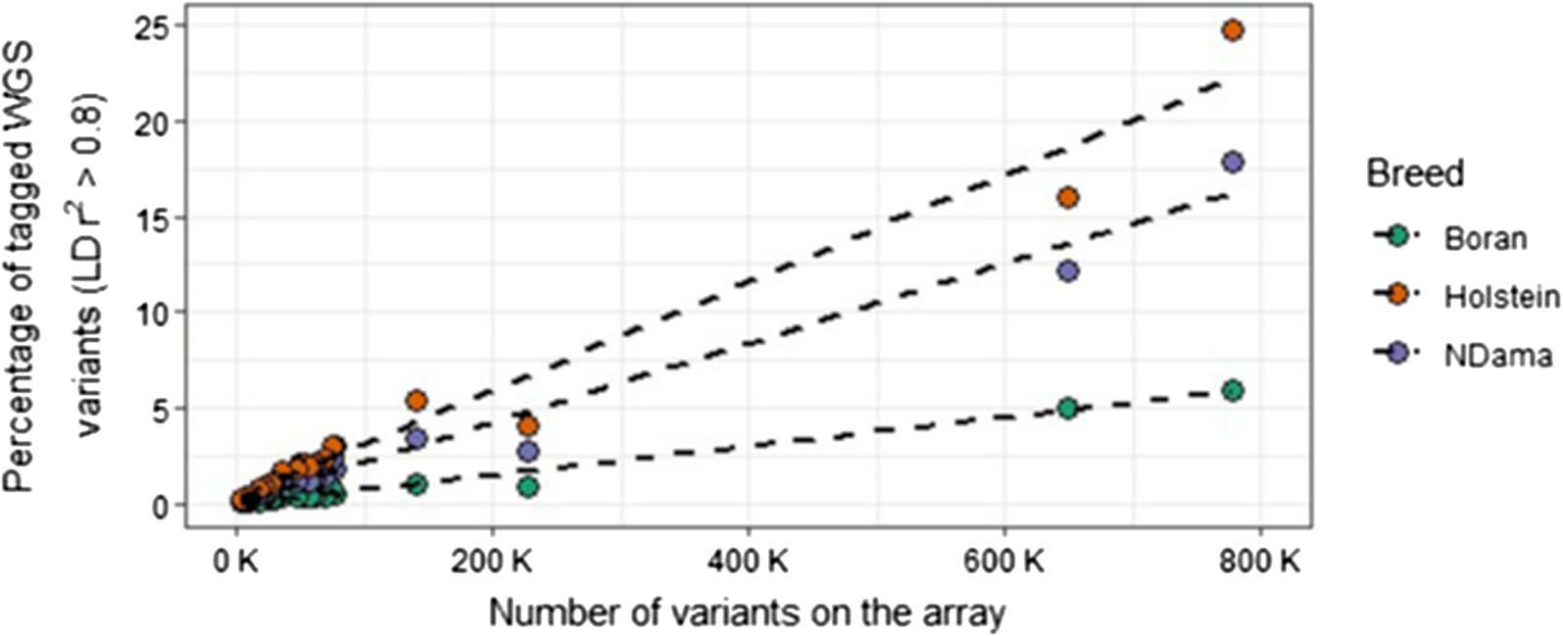
Percentage of variants tagged by the bovine genotyping arrays in European and African cattle breeds. Percentage of variants tagged in the whole-genome sequence data, with an  LD r^2^ > 0.8, by the 23 currently available bovine genotyping arrays

After variant QC, we identified 32.0, 17.8 and 13.3 million SNPs in the Boran, NDama and Holstein–Friesian cohorts, respectively. Although more than twice the number of SNPs segregated among the Boran (indicine) than among the taurine breeds, the percentage of WGS variants tagged by the arrays was consistently higher in the NDama and Holstein–Friesian breeds than in the Boran breed (Fig. 1). This is potentially partly due to the fact that the Boran breed has a slightly lower LD profile compared to the taurine breeds (see Additional file 7: Figure S4). Furthermore, the performance of any given array, in terms of the number of variants that it tags with a high R^2^, is largely a function of the number of variants it carries. It may be surprising, but the arrays that target indicine breeds did not show a substantial improvement for the Boran compared to the NDama and Holstein–Friesian cattle. This confirms that variants on commercially available arrays do not tag well the variants that segregate in indicine breeds, which has implications for performing GWAS in African breeds.

This comparison reveals more differences between the taurine breeds than between the indicine breeds across the 23 commercial bovine genotyping arrays. For example, the HD array tagged up to 25 and 17% (for Holstein–Friesian and NDama breeds, respectively) of the WGS variants, considering an LD r^2^ > 0.8, whereas only about 6% for the indicine Boran (Fig. 1). Intriguingly, there were more tagged variants for the taurine breeds than for the Boran breed, even in the indicus-based array such as IND90KH.

### Imputation performance in African cattle

Consequently, it seems that a large proportion of the variants specific to African indicine cattle are poorly captured across all of the major genotyping arrays. Thus, we investigated to what extent imputation could help mitigate this issue. For this analysis, we used the WGS data of 289 broadly unrelated individuals [14], belonging to a diverse range of breeds and geographic locations (55 populations, among which 13 were European, 12 African, 28 Asian, and 2 Middle Eastern), as a Reference Panel (i.e., Global Reference Panel). The target sets were created by retaining the WGS genotypes that overlapped with the variants of each of the 23 available bovine genotyping arrays. The Global Reference Panel was also split into African (n = 87), Asian (n = 106) and European (n = 77) subsets, which allowed us to perform imputation with only the subset of variants found on each specific array and to compare the imputed genotypes to those found in the WGS data, and thus to evaluate which array performed better in African cattle.

Table 1 shows the numbers of variants on each of the 23 commercially available arrays and how many were retained from the WGS data, before and after QC (65,107,956 and 10,282,187 variants, respectively). Eighteen of the 23 arrays are well represented in the unfiltered WGS, with more than 80% of variants retained before QC, suggesting that the sites were mostly polymorphic among the 289 samples. The panel with the lowest proportion of variants retained from the unfiltered WGS was the GGPF250 array (61%). After QC, these proportions dropped further, with only 23% variants from the GGPF250 array retained. This likely reflects the allelic frequencies of the variants in the population under study. The GGPF250 array contains many very rare variants. For a variant occurring at a very low allele frequency, the probability of observing it in 289 samples is close to zero, even if it is present in the population.

Arrays with less than 10,000 variants remaining after QC were excluded from further analysis (Table 1) and imputation was performed using Minimac4 (https://genome.sph.umich.edu/wiki/Minimac4). Two related metrics were calculated for each array. ER^2^ is calculated directly by Minimac4 and measures the imputation accuracy of variants on the array, with each variant being left out one by one when performing imputation, and dosage R^2^ represents the genotype concordance between imputed and WGS genotypes at the variants that are not on the array.

**Fig. 2:**
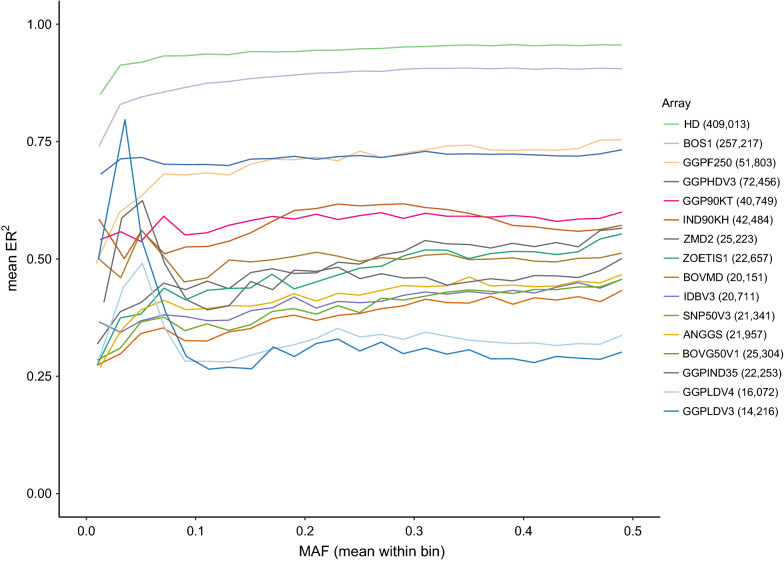
Imputation accuracy (ER^2^) when imputing from bovine array variants to WGS level, using the Global Reference Panel (i.e., 289 individuals). The target sets were created by retaining the WGS genotypes that overlapped with the variants of each of the 23 available bovine genotyping arrays from the Global Reference Panel (i.e., Global target set, with 289 individuals). Results are presented only for 16 arrays (i.e., those retaining more than 10,000 variants after QC), for which imputation was successful. The number of variants retained from the WGS data for each array is between brackets

**Fig. 3:**
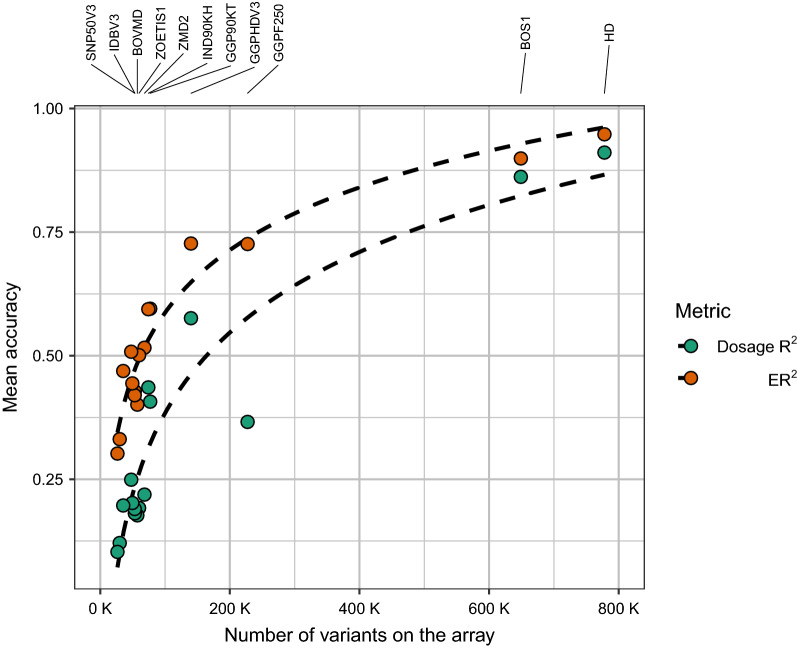
Array evaluation based on number of variants per array vs. mean accuracy per array. Scatterplot comparing the number of variants per array vs. mean accuracy (either ER^2^ or R^2^) per array. Arrays with more than 50 k variants are labelled

Figure 2 shows the mean ER^2^ for all the bovine arrays when using the Global target set (i.e., the complete set of 289 samples as the reference panel). The highest accuracies were observed for the HD and BOS1 arrays (mean ER^2^ across target sets ranging from 0.55 to 0.96 and from 0.48 to 0.91, respectively), which is consistent with the fact that these two arrays have the largest number of variants. These results were supported by those obtained with the leave-one-out cross-validation (see Additional file 8: Figure S5) and in the comparison of the number of variants per array vs. mean accuracy (either ER^2^ or dosage R^2^) per array (Fig. 3).

The scatterplot in Fig. 3 shows that the HD and BOS1 arrays present the highest mean accuracies. Since our results show that the best performing arrays are those with the largest number of variants, the result that the GGPHDV3 array outperforms the GGPF250 array on these metrics (i.e., ER^2^ and dosage R^2^) may appear contradictory although they have almost half as many variants. However, as reported in Table 1, the number of variants retained from the WGS data for GGPHDV3 is larger than the number retained for GGPF250 (72,456 vs. 51,803).

**Fig. 4:**
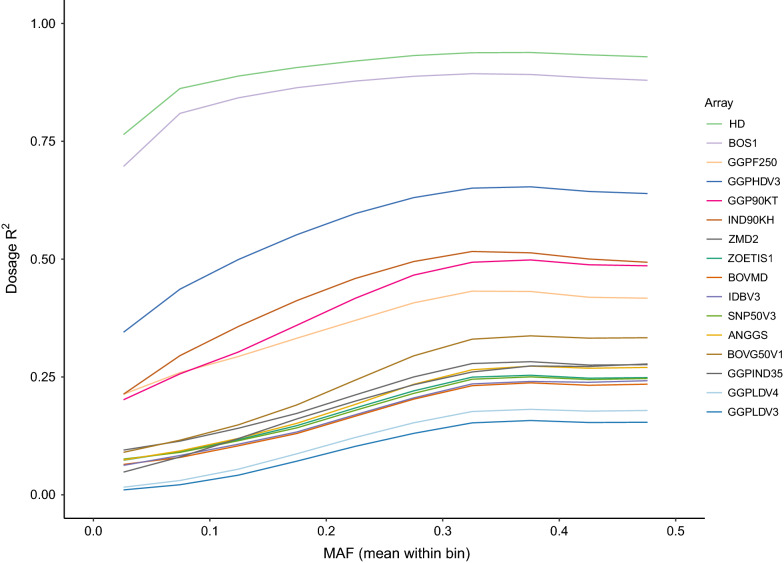
Imputation accuracy (dosage R^2^) when imputing from bovine array variants to WGS level, using the Global Reference Panel (i.e., 289 individuals). The target sets were created by retaining the WGS genotypes that overlapped with the variants of each of the 23 available bovine genotyping arrays from the Global Reference Panel (i.e., Global target set, with 289 individuals). Results are presented only for 16 arrays (i.e., those retaining more than 10,000 variants after QC), for which imputation was successful. Dosage R^2^ is only calculated for the variants that are not on the array

A similar trend was observed when considering the dosage R^2^ between imputed genotypes and those called in the WGS data (Fig. 4). Although the HD and BOS1 arrays were primarily designed by focusing on European taurine cattle breeds, they perform better than lower density arrays that were designed explicitly for indicine breeds (i.e., IND90KH and GGPIND35), which suggests that a large number of variants in the target set is the most critical array feature for imputation. However, the GGPF250 array is a notable outlier. Although GGPF250 is the third largest commercially available bovine array, after the HD and BOS1 arrays, the number of variants retained from the WGS was relatively small, with only 61 and 23% retained when considering the WGS before and after QC, respectively. While the ER^2^ was reasonably high for this array (~ 0.6–0.7 across MAF thresholds $\ge$ 0.05), i.e. the variants on the array could be imputed with reasonable accuracy, imputation performance was much poorer for variants that were not on the array (dosage R^2^ < 0.4) and worse than for arrays with less than half the number of variants.

### Effect of breed on imputation performance

As the genotyping arrays were originally designed for different breeds and lineages (i.e., taurine vs. indicine), we investigated whether imputation performance was better for specific populations of animals and whether some arrays might perform better than others when applied to these population subsets.

**Fig. 5:**
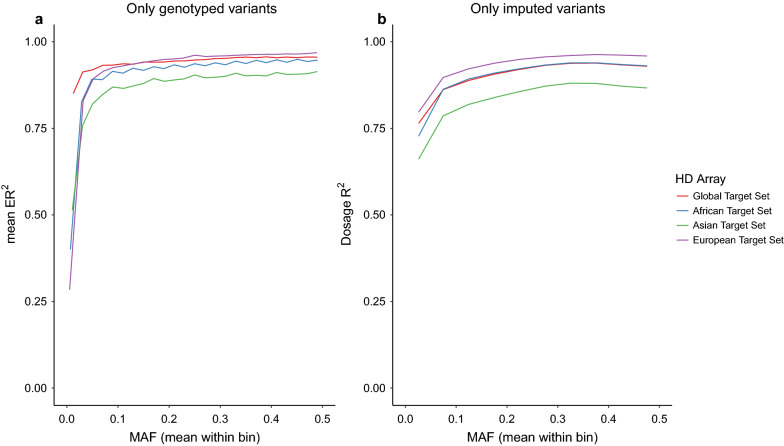
Imputation accuracies ER^2^ (**a**) and dosage R^2^ (**b**) when imputing from HD array to WGS level, using the Global Reference Panel (i.e., 289 individuals) and several target sets. The target sets were created by retaining the WGS genotypes that overlapped with the variants of the HD array, from the four available Reference Panels (i.e. Global, African, Asian, and European). These target sets were then used to impute to WGS level using the Global Reference Panel

As shown in Fig. 5, imputation performance of the African and European animals (i.e., African and European target sets) were broadly similar when imputing from the HD array variants to the Global Reference Panel. In contrast, imputation was notably lower among the Asian samples, which likely reflects that Asian cattle originate predominantly from *Bos indicus,* and are less well represented by the variants on the HD array. For most arrays, the highest accuracies were estimated when imputing European samples, with the mean ER^2^ or dosage R^2^ being similar to or higher than for the global set (see Additional file 9: Figures S6–S20). In general, the imputation performance of the Asian samples was poorest across all arrays, including those that were designed to capture indicine variation, such as IND90KH and GGPIND35.

### Array performances at functional variants

**Fig. 6:**
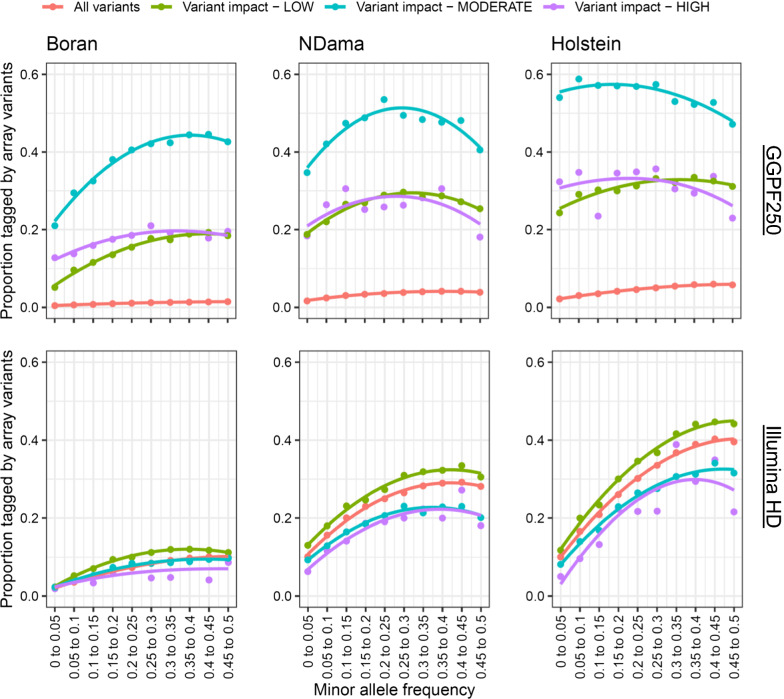
Proportions of all variants and functional variants tagged with an LD r^2^ > 0.8 by the GGPF250 and HD genotyping arrays in European and African cattle breeds. Proportions of all variants and functional variants (as annotated by the Ensembl VEP software (LOW, MODERATE and HIGH)) tagged with an LD r^2^ > 0.8 by GGPF250 and HD genotyping arrays in European and African cattle breeds

In GWAS, the ability to genotype or impute functional variants is essential, given their role in driving phenotypes. Thus, we characterised the performance for precisely capturing these variants. First, we investigated the proportions of functional variants annotated by the Ensembl VEP v95 software (LOW, MODERATE, and HIGH predicted functional impact) that were tagged by the genotyping arrays in the WGS data, for both taurine (i.e., Holstein–Friesian and NDama) and indicine (i.e., Boran) breeds. Figure 6 shows the trends for two arrays (GGPF250 and HD).

Although the HD array can tag more variants at an r^2^ > 0.80 than the other arrays (Fig. 1), it does not perform well for tagging functional variants, especially those predicted to have a moderate or high impact. In spite of its lower density, the GGPF250 array tagged higher proportions of functional variants across all allele frequencies, which is consistent with its design that is aimed at targeting functional variants. However, the performance of an array for tagging functional variants is potentially also partly due to the similarity between the allele frequencies of the two sets of variants. Since the LD between two variants, as measured by r^2^, is maximised when they have equal allele frequencies, the common variants (moderate to high MAF) on most arrays will do a poor job at tagging the functional variants simply due to the difference in allele frequency. Therefore, the GPPF250 array potentially performs better than the other arrays for tagging functional variants because, besides including a high proportion of variants in genic regions, the allele frequencies of these variants are more closely matched. However, functional variants that segregate among the Boran cattle were still less well captured by this array than those among the taurine breeds. Notably, the GGPF250 array performed best at tagging variants with a MODERATE impact (missense variants) rather than those with a HIGH impact (e.g. stop gain or splice variants).

**Fig. 7:**
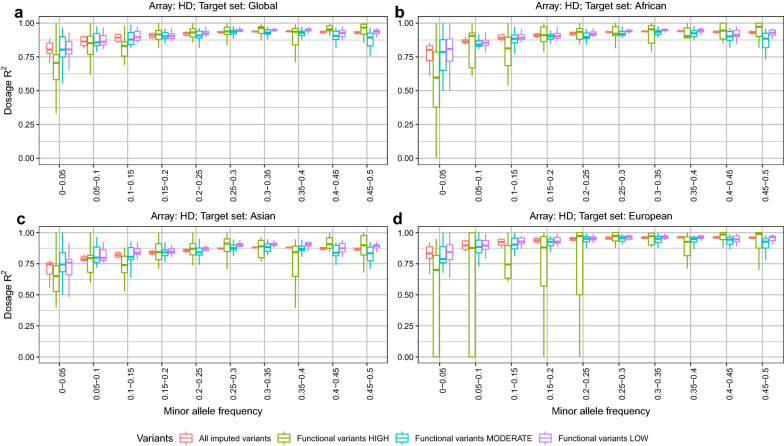
Dosage R^2^ for all imputed variants and for functional variants when imputing from HD array to WGS level, using the Global Reference Panel (i.e., 289 individuals) and several target sets. Dosage R^2^ for all imputed variants and for functional variants (as annotated by the Ensembl VEP software (LOW, MODERATE and HIGH)) when imputing from HD array to WGS level. The target sets were created by retaining the WGS genotypes that overlapped with the variants of the HD array, from the four available Reference Panels (i.e. Global, African, Asian, and European). These target sets were then used to impute to WGS level using the Global Reference Panel

To assess how well functional variants can be imputed, we calculated the imputation accuracy (i.e., dosage R^2^) for each of the three classes (LOW, MODERATE and HIGH) of functional variants (both SNPs and indels). The masked analysis was performed considering the four target sets (i.e., Global, African, Asian, and European) and the Global Reference Panel. As shown in Fig. 7a, when considering the Global set of 289 individuals, SNPs with a low to moderate frequency (MAF < 0.15) and of HIGH predicted impact (e.g. stop gained or splice variants) are particularly poorly imputed in general by the HD array. In contrast, the imputation of the SNPs of MODERATE and LOW predicted impact generally follows the trend of all the other SNPs. Therefore, these results suggest that although the HD array is the best performing array according to the results from our masked analysis, it might not be ideal for imputing high impact SNPs, especially if they are rare.

A similar trend was observed for most arrays (see Additional file 10: Figures S21–S35, panels a). However, the GGPF250 and GGPIND35 arrays performed better when imputing HIGH, MODERATE and LOW impact SNPs than when imputing all other SNPs at any frequencies (see Additional file 10: Figures S22 and S33, respectively). This reflects the design of these arrays, with the GGPF250 array including 199,000 predicted functional variants [3], and the GGPIND35 array including 35,090 SNPs that are optimally selected for *Bos indicus* breeds [24] as well as *Bos indicus* specific SNPs for parentage testing; GGPIND35 also includes variants known to be causative for particular genetic diseases (https://www.neogen.com/globalassets/pim/assets/original/10000/official_ggp-indicus_brochure.pdf). Similar trends were also observed when using the continent-specific target sets (i.e., African, Asian and European). However, dosage R^2^ tended to be consistently lower for all target sets (see Additional file 10: Figures S21–S35, panels b–d), which might be explained by the relatively smaller size of these target sets compared to the global one. It should be noted that imputation for high impact variants generally performed less well when considering the European compared to the African target set.

We also examined the imputation performance of indels that are more likely to be functionally important. These were consistently poorly imputed across arrays, irrespective of their frequencies and the target set used [i.e., Global, African, Asian, and European; (see Additional file 11: Figures S36–S51)]. It should be noted that this may partly reflect the difficulty of accurately calling indels in WGS data, rather than necessarily being only a problem with their imputation.

### Validation analysis imputing 50 k and HD genotype target sets to WGS

**Fig. 8:**
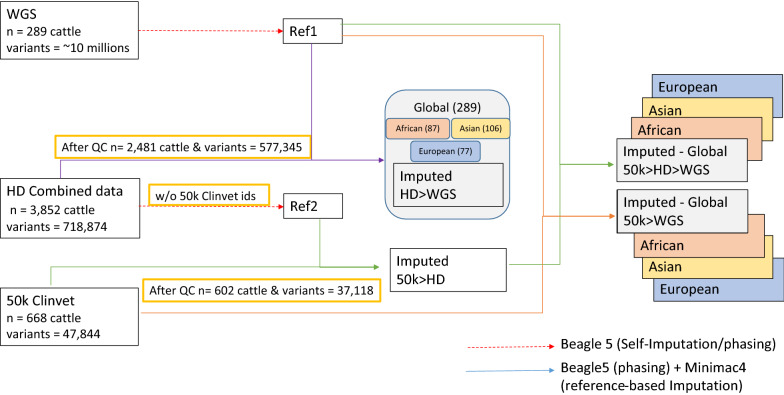
Flowchart of the imputation analysis for the real data cohort. Flowchart of the imputation analysis from either the 50 k or the combined HD data to WGS (50 k > WGS and HD > WGS, respectively), as well as the two-step approach from 50 k to WGS (i.e., 50 k > HD > WGS) using the Global Reference Panel and the continent-specific reference panels (i.e., African, Asian, and European Reference Panels)

An issue with testing imputation performance by subsetting variants from WGS data is that not all variants for an array can be called in the dataset, which reduces the apparent performance of each array. Thus, we also tested imputation performance using large collections of real array data collected from four African countries (Tanzania, Ghana, Nigeria, and Burkina Faso). This included 602 African samples genotyped on the GeneSeek 50 k array and 1740 on the Illumina HD array. Principal component analysis (PCA) of these samples shows that they represent well cattle from both West and East Africa (see Additional file 12: Figures S52 and S53). The 1740 samples were combined with other available samples genotyped with the Illumina HD array, reaching a total of 2481 samples. The PC plot of PC1 and PC2 for the combined HD genotyped individuals (see Additional file 12: Figure S54) shows a distinct separation between the European taurine, African taurine and indicine samples. PC1 seems to separate individuals by their degree of taurine or indicine lineage, with the Jersey and Holstein breeds to the left of the plot, whereas PC2 separates European and African taurine animals. The 1740 samples collected across the four African countries separate from the former breeds, and form a cluster between the African taurine and the indicine breeds. Using these data, we compared the use of different imputation approaches and reference panels on imputation accuracy. The flowchart for this analysis is in Fig. 8.

**Fig. 9:**
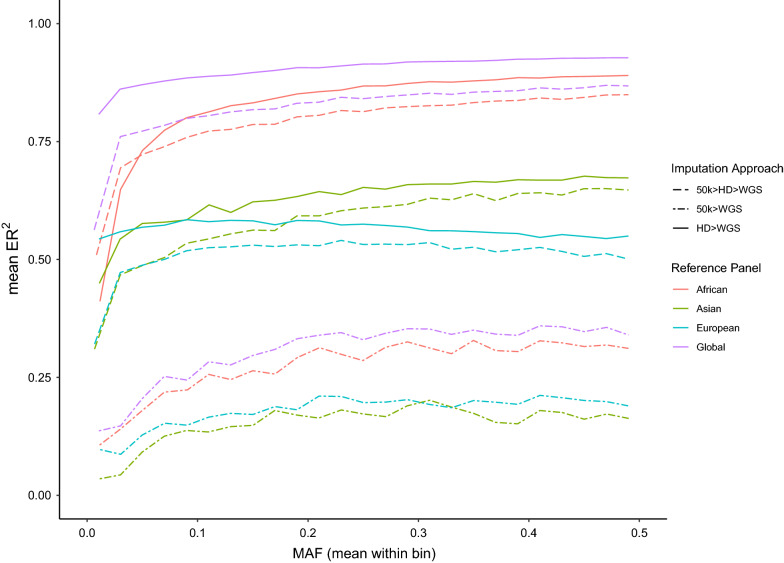
Imputation accuracy (ER^2^) in the real data cohort, genotyped with either 50 k or HD arrays. Imputation accuracy (ER^2^) when imputing from either the 50 k or HD arrays directly to WGS (50 k > WGS and HD > WGS, respectively), as well as the two-step approach from 50 k to WGS (i.e., 50 k > HD > WGS) using the Global Reference Panel and the continent-specific subsets (i.e., African, Asian, and European Reference Panels). Coloured by Reference Panel (i.e., African, Asian, European, and Global Reference Panels). Different line types were used for the three approaches

We compared two imputation approaches. The 50 k and HD genotype data imputed directly to WGS, as well as a two-step imputation from 50 k to HD then WGS (50 k > HD > WGS), both using the Global Reference Panel and the continent level subsets (i.e., African, Asian and European Reference Panels). Generally, imputation from the 50 k array directly to WGS performed poorly irrespective of the reference panel used (Fig. 9), with mean ER^2^ lower than 0.4 across all allele frequencies. The global and Africa-specific reference panels performed the best, with little difference in their respective performances. The reference panels of European and Asian cattle breeds also performed poorly when attempting to impute from the Illumina HD array, with mean ER^2^ values of 0.65 or lower across the allele frequency spectrum. In spite of this, supplementing the African reference panel with these European and Asian panels produced the best imputation performance (mean ER^2^ ranging from 0.81 to 0.93). This Global reference panel performed better than the Africa specific reference panel alone (mean ER^2^ ranging from 0.41 to 0.89).

The performance of the two-step imputation from 50 k to WGS (50 k > HD > WGS) was between that of the imputation from 50 k or Illumina HD directly to WGS, with ER^2^ trends similar to those of the Illumina HD array, for all panels tested. Consequently, larger global reference panels are likely to be more effective for imputation than more targeted representative panels, and this is consistent with previously reported results e.g., [3, 25]. Likewise, a two-step imputation strategy performs better when starting from a low-density array.

**Fig. 10:**
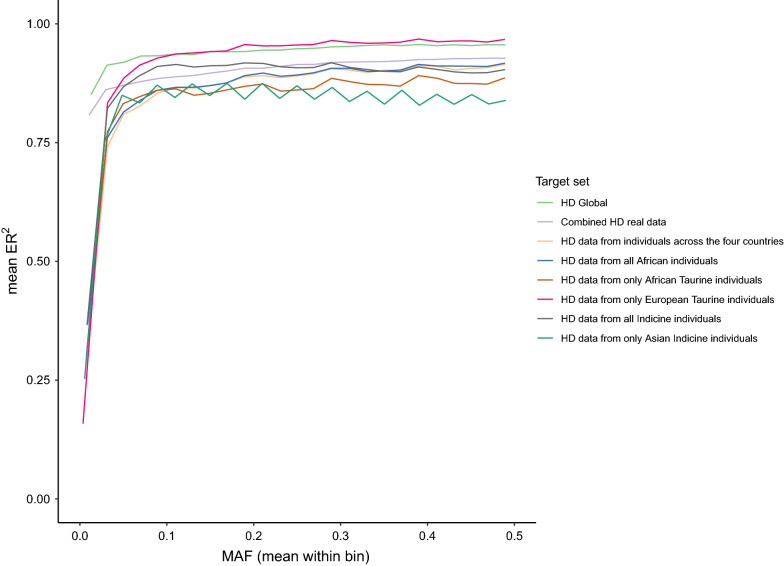
Imputation accuracy (ER^2^) in the real data cohort, when considering different subsets of individuals. Imputation accuracy (ER^2^) when imputing from the combined data (genotyped with the HD array) as well as the six subsets (i.e., samples collected across the four African countries, all African samples, African taurine samples, European taurine samples, all indicine samples, and Asian indicine samples), reflecting the variety of breeds/populations represented in the HD combined data. Imputation to WGS level was carried out using the Global Reference Panel. The results from the masked analysis using the target set that was created by retaining the WGS genotypes that overlapped with the variants of the HD array are also reported

Considering the variety of breeds/populations represented in the HD combined data [for a PC plot (see Additional file 12: Figure S54)], another analysis was carried out by splitting the samples to be imputed into six subsets (samples collected across the four African countries, all African, African taurine, European taurine, all indicine, and Asian indicine samples). The sizes of the sets were balanced to 55 individuals each, and imputation accuracies (ER^2^) are shown in Fig. 10. All subsets show very low ER^2^ at low MAF, probably due to the comparatively small size of the sets.

While the imputation accuracies obtained when considering the first two African target subsets (i.e., samples collected from the four African countries and all African) were consistent with those obtained for the combined HD data, a slightly different trend was observed when all indicine and the European taurine target subsets were used. All indicine sets (which included Asian and African indicine cattle) performed better than the combined HD data at lower MAF but followed the same trend as the latter at MAF higher than 0.3. In contrast, the European taurine set performed better, with mean ER^2^ ranging from 0.88 (at MAF ≈ 0.03) to 0.97. We hypothesise that the European taurine subset performs better because, although its sample size is small, the individuals are more similar to each other (given the small effective population size of these breeds), and it is therefore easier to define haplotypes and subsequently impute missing data. Moreover, the Illumina HD array was primarily designed for European cattle breeds.

## Discussion

A critical step for improving production in cattle and other livestock species is to map the genetic loci that underlie important traits and phenotypes, so that they can potentially be used in marker-assisted selection programs. During the last few decades, the use of GWAS has allowed the successful identification of hundreds of SNPs associated with complex traits in cattle. However, these studies have focused mainly on cattle that derive from European or Asian breeds, using genotyping arrays mostly designed for European breeds, and are therefore biased towards variants common to these breeds. To determine the best strategy for carrying out GWAS in African cattle, we assessed the performance of currently available bovine genotyping arrays using two large datasets of genotyping data; a collection of 409 WGS spanning global cattle breeds, and a cohort of 2481 individuals genotyped with the Illumina HD array (1740 of which were collected from four African countries) and the GeneSeek bovine 50 k array (602 samples collected from four African countries). We have demonstrated that the HD and BOS1 arrays (i.e., arrays with the largest number of variants) are those that best capture the diversity across African breeds (calculating the proportions of variants tagged by the genotyping arrays in the WGS data, with an LD r^2^ > 0.80) and are consequently the most effective arrays for performing genome-wide imputation. However, these arrays might not be the best choice when the aim is to tag functional variants, especially when considering rare variants. We have also shown that using a reference panel that better represents global bovine diversity improves imputation accuracy, particularly for non-European taurine populations.

Our analysis demonstrates that a substantial proportion of the variants present in a population of the indicine Boran breed are poorly captured across all the major genotyping arrays, with the number of WGS variants that are highly correlated with SNPs on the genotyping arrays being much larger for the populations of taurine breeds (i.e., NDama and Holstein–Friesian). Although the WGS data of the Boran (indicine) breed has more than twice the number of variants than that of the taurine breeds (i.e., 32 million SNPs for Boran vs. 17.8 and 13.3 million SNPs for NDama and Holstein–Friesian, respectively), the proportions of variants tagged by the genotyping arrays in the WGS data (with LD r^2^ > 0.8) is higher in the taurine samples (i.e., NDama and Holstein–Friesian) than in the Boran samples. For example, the Illumina HD array tagged up to 25% and 17% of the variants in the WGS data for Holstein–Friesian and NDama, respectively, but only 6% for the Boran. While the other arrays overall performed poorly compared to the Illumina HD, their performance followed the same trend across the three breeds. The taurine breeds had more highly correlated WGS SNPs than the Boran, even with the indicus-specific arrays, such as IND90KH and GGPIND35. Therefore, these results suggest that using any of the currently available genotyping arrays for African indicine breeds is likely to result in significantly underpowered analysis and lack of ability to detect most linkages between phenotype and genotypes. It could be hypothesised that combining two different arrays (which potentially tag different variants) could be beneficial. However, it should be taken into account that the amount of DNA required to process both arrays, and the budget, are likely to be limiting factors.

Although the performance of the genotyping arrays for directly capturing the diversity of African indicine cattle is not promising, the results obtained in the imputation analyses were quite different, which is probably because imputation is based on haplotypes rather than individual SNPs. In the imputation analyses, the highest accuracies considering both metrics (i.e., ER^2^ and dosage R^2^) were obtained from arrays with the largest number of variants (i.e., HD and BOS1), regardless of the target sample set used (Global, African, Asian or European). This suggests that, although the HD and BOS1 arrays were designed primarily to focus on European taurine cattle breeds, they perform better than arrays designed specifically for indicine breeds (i.e., IND90KH and GGPIND35), which indicates that for imputation a large number of variants in the target set is more important than the way it is designed. Marker density is indeed one of the main factors that affect genotype imputation accuracy, together with the size of the reference population, the relationship between the reference population and the validation population, MAF, and reference population composition [26]. However, it should be mentioned that the accuracies obtained in our study might be slightly biased by the fact that we did not remove variants that are within potentially misassembled regions, as suggested by Null et al. [27].

Since the four target sets were generated by retaining the individuals from the reference panel (i.e., 289 for the Global target set, 87 for the African, 106 for the Asian and 77 for the European), we tested whether having the same individuals in the reference and target set populations could cause an overestimation of the accuracies. This was done by carrying out a leave-one-out cross-validation using 100 of the 289 animals from the WGS data as the target set, in which one individual was removed from the reference panel and its genotypes imputed using the remaining animals as the reference panel, repeated for each of the 100 animals, randomly selected from the WGS data. The leave-one-out cross-validation analysis was consistent with the results of the masked analysis, across all genotyping arrays analysed.

One of the most interesting results was that although GGPF250 is the third largest array currently available in terms of number of SNPs, it did not perform well in either of the analyses (i.e. capturing diversity in African cattle or imputation). However, GGPF250 was the array that performed better when considering the functional variants, which is probably related to its design. The GGPF250 array was designed to query genotypes at many rare, potentially functional variants and is very gene-centric (i.e. focusing on SNPs located within the coding regions of genes) [3]. It contains only ~ 30 k common variants, which have been included to allow for imputation and genomic prediction applications. Rowan et al. [3] suggested that using the GGPF250 array would help researchers to refine GWAS signals, identify putative quantitative trait nucleotides due to increased marker density within QTL regions, and improve imputation accuracy to WGS level within genic regions. Thus, our results suggest that it is not possible to establish a single array that would perform the best in any scenario. Still, the choice of an array over the others should be based on a balance between the objective of the study and the breed/population considered. For example, the HD and BOS1 arrays may be the best choice for taurine and indicus breeds, when performing genome-wide association or related studies, whereas the GGPF250 array may be preferable for fine-mapping analyses or for searching for functional variants in general. However, leaving aside cost issues, our data suggest that there is no advantage to using the indicus-specific arrays for African indicus breeds, regardless of the objective.

The analysis on imputation performance on the cohort genotyped with the HD array (including the African cattle deriving from Nigeria, Ghana, Burkina Faso and Tanzania) showed that the African breed WGS reference panel performed very well. However, using the Global WGS reference panel (i.e. supplementing the African reference panel with the European and Asian panels) produced the best imputation performance. This is likely due to the increased size of the reference panel as well as the increased haplotype diversity present in a mixed reference panel, which improves the accuracy of the imputation of haplotypes that are not present in a more specific reference panel [3]. This result is in agreement with most reports on livestock [3, 8, 28, 29] and humans (e.g., [25, 30]). Moreover, Howie et al. [30] showed that a mixed reference panel can improve imputation accuracy for SNPs with a low MAF because an allele with a low frequency in one population can be more frequent in another population. However, Mdyogolo et al. [31] reported quite a different result when assessing the accuracy of genotype imputation in the Afrikaner, Brahman and Brangus breeds of South Africa. In that study, animals were genotyped with the GeneSeek Genomic Profiler 150 k array, genotypes were masked and then imputed to array level, using both a breed-specific and a multi-breed reference panel. They observed that the imputation accuracy was reduced by more than 10% when a multi-breed reference panel was used, compared to breed-specific reference panels [31]. However, their sample sizes were very small, with only a small percentage of SNPs (i.e., less than 10% for each breed) being masked and imputed. In contrast in our study, we imputed from HD to WGS level (i.e., from 577,345 variants after QC to over 10 million variants of the WGS, increasing genome coverage by almost 20%). It is worth noting that increasing genome coverage by only 10% as reported by these authors, has minimal benefit from a practical point of view.

Generally, imputation from the 50 k array directly to WGS performed poorly, with mean ER^2^ lower than 0.4 across all allele frequencies. However, our results showed that the two-step approach (i.e., from 50 k to HD and then to WGS) yielded high imputation accuracies, with an increase in accuracy of almost 50%. Similar results were previously reported (e.g., [32, 33]), even when starting from lower density arrays (e.g., [33]). In particular, VanRaden et al. [33] reported an increase in accuracy of about 2% when imputing from 3000 SNPs to 50 k and then to HD, rather than imputing directly from 3000 to HD. Although the increase of 2% reported by VanRaden et al. [33] seems small, one must take into account that the accuracy obtained in the one-step imputation (i.e., from 3000 SNPs to HD) was already high, which was probably achieved because of the large sample size genotyped in that study with the 3 k array (i.e., 38,441), compared with 602 samples genotyped with the 50 k array in our study. One potential reason for the two-step imputation being more effective than the one-step imputation from lower density to WGS is the larger sample size of the intermediate reference panel (i.e., HD array data), which allows for a better estimation of haplotypes when imputing from 50 k to HD, thus refining the haplotype blocks for the second step. In the one-step imputation from lower density to WGS, it is indeed more difficult for the imputation algorithm to select the correct haplotype since there might be multiple possible matches between the lower density (i.e., 50 k in our case) haplotypes and the WGS haplotypes. In contrast there are fewer possible matches when HD SNPs are added as an intermediate step. In this case, there is a higher probability of selecting the long-range haplotypes in the first step and the short-range haplotypes in the second step, which increases imputation accuracy [32].

Our results clearly show the limitations of the existing arrays for imputation in samples from Africa, i.e. imputation accuracy decreases as genetic distance increases. A solution for these populations could be to use low-pass sequencing and imputation, with one caveat being that a sufficient number of individuals needs to be included in the WGS reference panels used for imputation from low-pass sequencing.

## Conclusions

In this study, our aim was to determine the best strategy for carrying out GWAS in African cattle by assessing the performance of the bovine genotyping arrays that are currently available to capture the diversity across African breeds and their utility in performing genome-wide imputation. Our results show that the choice of an array over the others should be based on a balance between the objective of the study and the breed/population considered. The HD and BOS1 arrays performed best for both capturing diversity and performing imputation, so they would be the best choice for both taurine and indicus breeds when performing genome-wide association or related studies. However, the GGPF250 array may be preferable when undertaking fine-mapping studies or assessing functional variants in general. Moreover, our results suggest that there is no advantage to using the indicus-specific arrays for African indicus breeds, regardless of the objective. We also show that using a reference panel that better represents global bovine diversity improves imputation accuracy, particularly for non-European taurine populations.

## Supplementary Information

**Additional file 1: Table S1.** Populations with whole-genome sequence data. Number of animals, with whole-genome sequence data per breed/population used in this study, indicating sources and/or project accession numbers. **Table S2.** Accession numbers of the Boran samples used in this study, which are publicly available.

**Additional file 2: Figure S1.** Variant Quality Score Recalibration for WGS variants using GATK: tranches plot (a) and specificity versus tranche truth sensitivity (b). Quality metrics of the WGS data. Tranche-specific TP are true-positive calls gained when adding a slice to the plate. Cumulative TP are true-positive calls contained in all the slices already added. Thus, this differentiation allows to evaluate how many more TP are gained *vs.* the additional false positives (FP) that have to be taken on, when going to the next tranche up. The ratio of transition (Ti) to transversion (Tv) SNPs (i.e., Ti/Tv ratio) is a useful diagnostic tool to measure the quality of the WGS data generated. A high Ti/Tv ratio (> 2.0) often indicates a high-accuracy SNP set, whereas a low value (~ 0.5) implies low-quality SNP calling.

**Additional file 3: Figure S2.** Principal component plot of the individuals included in the WGS data. Plot for principal component (PC) 1 and PC2 as well as PC1 and PC3 for the 289 distinct individuals included in the WGS data. The data spanned a diverse range of breeds and geographic locations (55 populations, among which 13 European, 12 African, 28 Asian, and 2 Middle Eastern). Coloured by population and location [34].

**Additional file 4: Table S3.** Populations represented in the combined HD data. Number of animals genotyped with the Illumina HD array per breed/population used in this study, and data source

**Additional file 5: Table S4.** Coordinates (chromosome and position in bp) of the variants retained after lift-over on both UMD3.1 and ARS-UCD1.2 assemblies. Variants retained after combining and lifting-over the different HD array data. Coordinates (chromosome and position in bp) on both UMD3.1 and ARS-UCD1.2 assemblies are provided.

**Additional file 6: Figure S3.** Comparison between imputation accuracies (ER^2^) when phasing was done with either BEAGLE or SHAPEIT4. Comparison between imputation accuracies (ER^2^, as estimated in Minimac4) when phasing was done with either BEAGLE or SHAPEIT4. Since the imputation accuracies were similar, BEAGLE phased data were used for all subsequent analyses.

**Additional file 7: Figure S4.** Linkage disequilibrium (r^2^) decay in European and African cattle breeds. Comparison of linkage disequilibrium decay in taurine (both European and African) and African indicine breeds.

**Additional file 8: Figure S5.** Imputation accuracy (ER^2^) with the leave-one-out cross-validation, using 100 of the 289 animals from the WGS data, for all bovine genotyping arrays considered. In this procedure, one individual was removed from the reference panel and its genotypes imputed using the remaining animals as the reference panel. This was repeated for each of the 100 animals, randomly selected from the WGS data and for each array. The results are presented only for 16 arrays (i.e., those retaining more than 10,000 variants after QC), for which imputation was successful. The number of variants retained from the WGS data for each array is between brackets.

**Additional file 9: Figure S6.** Imputation accuracies ER^2^ (A) and dosage R^2^ (B) for the BOS1 array when using the Global Reference Panel and four target sets (i.e., Global, African, Asian and European). The target sets were created by retaining only the WGS genotypes that overlapped with the variants of the BOS1 array, from the Global Reference Panel as well as its subsets, generated according to the continent of origin (African (87 individuals), Asian (106 individuals) and European (77 individuals) subsets). These target sets (i.e. Global, African, Asian, and European) were then used to impute to WGS level using the Global Reference Panel. The results for the HD array when using the Global target set are also reported. **Figure S7.** Imputation accuracies ER^2^ (A) and dosage R^2^ (B) for the GGPHDV3 array when using the Global Reference Panel and four target sets (i.e., Global, African, Asian and European). The target sets were created by retaining only the WGS genotypes that overlapped with the variants of the GGPHDV3 array, from the Global Reference Panel as well as its subsets, generated according to the continent of origin (African (87 individuals), Asian (106 individuals) and European (77 individuals) subsets). These target sets (i.e. Global, African, Asian, and European) were then used to impute to WGS level using the Global Reference Panel. The results for the HD array when using the Global target set are also reported. **Figure S8.** Imputation accuracies ER^2^ (A) and dosage R^2^ (B) for the GGPF250 array when using the Global Reference Panel and four target sets (i.e., Global, African, Asian and European). The target sets were created by retaining only the WGS genotypes that overlapped with the variants of the GGPF250 array, from the Global Reference Panel as well as its subsets, generated according to the continent of origin (African (87 individuals), Asian (106 individuals) and European (77 individuals) subsets). These target sets (i.e. Global, African, Asian, and European) were then used to impute to WGS level using the Global Reference Panel. The results for the HD array when using the Global target set are also reported. **Figure S9.** Imputation accuracies ER^2^ (A) and dosage R^2^ (B) for the IND90KH array when using the Global Reference Panel and four target sets (i.e., Global, African, Asian and European). The target sets were created by retaining only the WGS genotypes that overlapped with the variants of the IND90KH array, from the Global Reference Panel as well as its subsets, generated according to the continent of origin (African (87 individuals), Asian (106 individuals) and European (77 individuals) subsets). These target sets (i.e. Global, African, Asian, and European) were then used to impute to WGS level using the Global Reference Panel. The results for the HD array when using the Global target set are also reported. **Figure S10.** Imputation accuracies ER^2^ (A) and dosage R^2^ (B) for the GGP90KT array when using the Global Reference Panel and four target sets (i.e., Global, African, Asian and European). The target sets were created by retaining only the WGS genotypes that overlapped with the variants of the GGP90KT array, from the Global Reference Panel as well as its subsets, generated according to the continent of origin (African (87 individuals), Asian (106 individuals) and European (77 individuals) subsets). These target sets (i.e. Global, African, Asian, and European) were then used to impute to WGS level using the Global Reference Panel. The results for the HD array when using the Global target set are also reported. **Figure S11.** Imputation accuracies ER^2^ (A) and dosage R^2^ (B) for the ZMD2 array when using the Global Reference Panel and four target sets (i.e., Global, African, Asian and European). The target sets were created by retaining only the WGS genotypes that overlapped with the variants of the ZMD2 array, from the Global Reference Panel as well as its subsets, generated according to the continent of origin (African (87 individuals), Asian (106 individuals) and European (77 individuals) subsets). These target sets (i.e. Global, African, Asian, and European) were then used to impute to WGS level using the Global Reference Panel. The results for the HD array when using the Global target set are also reported. **Figure S12.** Imputation accuracies ER^2^ (A) and dosage R^2^ (B) for the ZOETIS1 array when using the Global Reference Panel and four target sets (i.e., Global, African, Asian and European). The target sets were created by retaining only the WGS genotypes that overlapped with the variants of the ZOETIS1 array, from the Global Reference Panel as well as its subsets, generated according to the continent of origin (African (87 individuals), Asian (106 individuals) and European (77 individuals) subsets). These target sets (i.e. Global, African, Asian, and European) were then used to impute to WGS level using the Global Reference Panel. The results for the HD array when using the Global target set are also reported. **Figure S13.** Imputation accuracies ER^2^ (A) and dosage R^2^ (B) for the BOVMD array when using the Global Reference Panel and four target sets (i.e., Global, African, Asian and European). The target sets were created by retaining only the WGS genotypes that overlapped with the variants of the BOVMD array, from the Global Reference Panel as well as its subsets, generated according to the continent of origin (African (87 individuals), Asian (106 individuals) and European (77 individuals) subsets). These target sets (i.e. Global, African, Asian, and European) were then used to impute to WGS level using the Global Reference Panel. The results for the HD array when using the Global target set are also reported. **Figure S14.** Imputation accuracies ER^2^ (A) and dosage R^2^ (B) for the IDBV3 array when using the Global Reference Panel and four target sets (i.e., Global, African, Asian and European). The target sets were created by retaining only the WGS genotypes that overlapped with the variants of the IDBV3 array, from the Global Reference Panel as well as its subsets, generated according to the continent of origin (African (87 individuals), Asian (106 individuals) and European (77 individuals) subsets). These target sets (i.e. Global, African, Asian, and European) were then used to impute to WGS level using the Global Reference Panel. The results for the HD array when using the Global target set are also reported. **Figure S15.** Imputation accuracies ER^2^ (A) and dosage R^2^ (B) for the SNP50V3 array when using the Global Reference Panel and four target sets (i.e., Global, African, Asian and European). The target sets were created by retaining only the WGS genotypes that overlapped with the variants of the SNP50V3 array, from the Global Reference Panel as well as its subsets, generated according to the continent of origin (African (87 individuals), Asian (106 individuals) and European (77 individuals) subsets). These target sets (i.e. Global, African, Asian, and European) were then used to impute to WGS level using the Global Reference Panel. The results for the HD array when using the Global target set are also reported. **Figure S16.** Imputation accuracies ER^2^ (A) and dosage R^2^ (B) for the ANGGS array when using the Global Reference Panel and four target sets (i.e., Global, African, Asian and European). The target sets were created by retaining only the WGS genotypes that overlapped with the variants of the ANGGS array, from the Global Reference Panel as well as its subsets, generated according to the continent of origin (African (87 individuals), Asian (106 individuals) and European (77 individuals) subsets). These target sets (i.e. Global, African, Asian, and European) were then used to impute to WGS level using the Global Reference Panel. The results for the HD array when using the Global target set are also reported. **Figure S17.** Imputation accuracies ER^2^ (A) and dosage R^2^ (B) for the BOVG50V1 array when using the Global Reference Panel and four target sets (i.e., Global, African, Asian and European). The target sets were created by retaining only the WGS genotypes that overlapped with the variants of the BOVG50V1 array, from the Global Reference Panel as well as its subsets, generated according to the continent of origin (African (87 individuals), Asian (106 individuals) and European (77 individuals) subsets). These target sets (i.e. Global, African, Asian, and European) were then used to impute to WGS level using the Global Reference Panel. The results for the HD array when using the Global target set are also reported. **Figure S18.** Imputation accuracies ER^2^ (A) and dosage R^2^ (B) for the GGPIND35 array when using the Global Reference Panel and four target sets (i.e., Global, African, Asian and European). The target sets were created by retaining only the WGS genotypes that overlapped with the variants of the GGPIND35 array, from the Global Reference Panel as well as its subsets, generated according to the continent of origin (African (87 individuals), Asian (106 individuals) and European (77 individuals) subsets). These target sets (i.e. Global, African, Asian, and European) were then used to impute to WGS level using the Global Reference Panel. The results for the HD array when using the Global target set are also reported. **Figure S19.** Imputation accuracies ER^2^ (A) and dosage R^2^ (B) for the GGPLDV4 array when using the Global Reference Panel and four target sets (i.e., Global, African, Asian and European). The target sets were created by retaining only the WGS genotypes that overlapped with the variants of the GGPLDV4 array, from the Global Reference Panel as well as its subsets, generated according to the continent of origin (African (87 individuals), Asian (106 individuals) and European (77 individuals) subsets). These target sets (i.e. Global, African, Asian, and European) were then used to impute to WGS level using the Global Reference Panel. The results for the HD array when using the Global target set are also reported. **Figure S20.** Imputation accuracies ER^2^ (A) and dosage R^2^ (B) for the GGPLDV3 array when using the Global Reference Panel and four target sets (i.e., Global, African, Asian and European). The target sets were created by retaining only the WGS genotypes that overlapped with the variants of the GGPLDV3 array, from the Global Reference Panel as well as its subsets, generated according to the continent of origin (African (87 individuals), Asian (106 individuals) and European (77 individuals) subsets). These target sets (i.e. Global, African, Asian, and European) were then used to impute to WGS level using the Global Reference Panel. The results for the HD array when using the Global target set are also reported.

**Additional file 10: Figure S21.** Dosage R^2^ for all imputed variants and for functional variants for the BOS1 array when using the Global Reference Panel and four target sets (i.e., Global, African, Asian and European). Dosage R^2^ for all imputed variants and for functional variants (as annotated by the Ensembl VEP software (LOW, MODERATE and HIGH)) when imputing from the BOS1 array to WGS level. The target sets were created by retaining only the WGS genotypes that overlapped with the variants of the BOS1 array, from the Global Reference Panel as well as its subsets, generated according to the continent of origin (African (87 individuals), Asian (106 individuals) and European (77 individuals) subsets). These target sets (i.e. Global, African, Asian, and European) were then used to impute to WGS level using the Global Reference Panel. **Figure S22.** Dosage R^2^ for all imputed variants and for functional variants for the GGPF250 array when using the Global Reference Panel and four target sets (i.e., Global, African, Asian and European). Dosage R^2^ for all imputed variants and for functional variants (as annotated by the Ensembl VEP software (LOW, MODERATE and HIGH)) when imputing from the GGPF250 array to WGS level. The target sets were created by retaining only the WGS genotypes that overlapped with the variants of the GGPF250 array, from the Global Reference Panel as well as its subsets, generated according to the continent of origin (African (87 individuals), Asian (106 individuals) and European (77 individuals) subsets). These target sets (i.e. Global, African, Asian, and European) were then used to impute to WGS level using the Global Reference Panel. **Figure S23.** Dosage R^2^ for all imputed variants and for functional variants for the GGPHDV3 array when using the Global Reference Panel and four target sets (i.e., Global, African, Asian and European). Dosage R^2^ for all imputed variants and for functional variants (as annotated by the Ensembl VEP software (LOW, MODERATE and HIGH)) when imputing from the GGPHDV3 array to WGS level. The target sets were created by retaining only the WGS genotypes that overlapped with the variants of the GGPHDV3 array, from the Global Reference Panel as well as its subsets, generated according to the continent of origin (African (87 individuals), Asian (106 individuals) and European (77 individuals) subsets). These target sets (i.e. Global, African, Asian, and European) were then used to impute to WGS level using the Global Reference Panel. **Figure S24.** Dosage R^2^ for all imputed variants and for functional variants for the GGP90KT array when using the Global Reference Panel and four target sets (i.e., Global, African, Asian and European). Dosage R^2^ for all imputed variants and for functional variants (as annotated by the Ensembl VEP software (LOW, MODERATE and HIGH)) when imputing from the GGP90KT array to WGS level. The target sets were created by retaining only the WGS genotypes that overlapped with the variants of the GGP90KT array, from the Global Reference Panel as well as its subsets, generated according to the continent of origin (African (87 individuals), Asian (106 individuals) and European (77 individuals) subsets). These target sets (i.e. Global, African, Asian, and European) were then used to impute to WGS level using the Global Reference Panel. **Figure S25.** Dosage R^2^ for all imputed variants and for functional variants for the IND90KH array when using the Global Reference Panel and four target sets (i.e., Global, African, Asian and European). Dosage R^2^ for all imputed variants and for functional variants (as annotated by the Ensembl VEP software (LOW, MODERATE and HIGH)) when imputing from the IND90KH array to WGS level. The target sets were created by retaining only the WGS genotypes that overlapped with the variants of the IND90KH array, from the Global Reference Panel as well as its subsets, generated according to the continent of origin (African (87 individuals), Asian (106 individuals) and European (77 individuals) subsets). These target sets (i.e. Global, African, Asian, and European) were then used to impute to WGS level using the Global Reference Panel. **Figure S26.** Dosage R^2^ for all imputed variants and for functional variants for the ZMD2 array when using the Global Reference Panel and four target sets (i.e., Global, African, Asian and European). Dosage R^2^ for all imputed variants and for functional variants (as annotated by the Ensembl VEP software (LOW, MODERATE and HIGH)) when imputing from the ZMD2 array to WGS level. The target sets were created by retaining only the WGS genotypes that overlapped with the variants of the ZMD2 array, from the Global Reference Panel as well as its subsets, generated according to the continent of origin (African (87 individuals), Asian (106 individuals) and European (77 individuals) subsets). These target sets (i.e. Global, African, Asian, and European) were then used to impute to WGS level using the Global Reference Panel. **Figure S27.** Dosage R^2^ for all imputed variants and for functional variants for the ZOETIS1 array when using the Global Reference Panel and four target sets (i.e., Global, African, Asian and European). Dosage R^2^ for all imputed variants and for functional variants (as annotated by the Ensembl VEP software (LOW, MODERATE and HIGH)) when imputing from the ZOETIS1 array to WGS level. The target sets were created by retaining only the WGS genotypes that overlapped with the variants of the ZOETIS1 array, from the Global Reference Panel as well as its subsets, generated according to the continent of origin (African (87 individuals), Asian (106 individuals) and European (77 individuals) subsets). These target sets (i.e. Global, African, Asian, and European) were then used to impute to WGS level using the Global Reference Panel. **Figure S28.** Dosage R^2^ for all imputed variants and for functional variants for the BOVMD array when using the Global Reference Panel and four target sets (i.e., Global, African, Asian and European). Dosage R^2^ for all imputed variants and for functional variants (as annotated by the Ensembl VEP software (LOW, MODERATE and HIGH)) when imputing from the BOVMD array to WGS level. The target sets were created by retaining only the WGS genotypes that overlapped with the variants of the BOVMD array, from the Global Reference Panel as well as its subsets, generated according to the continent of origin (African (87 individuals), Asian (106 individuals) and European (77 individuals) subsets). These target sets (i.e. Global, African, Asian, and European) were then used to impute to WGS level using the Global Reference Panel. **Figure S29.** Dosage R^2^ for all imputed variants and for functional variants for the IDBV3 array when using the Global Reference Panel and four target sets (i.e., Global, African, Asian and European). Dosage R^2^ for all imputed variants and for functional variants (as annotated by the Ensembl VEP software (LOW, MODERATE and HIGH)) when imputing from the IDBV3 array to WGS level. The target sets were created by retaining only the WGS genotypes that overlapped with the variants of the IDBV3 array, from the Global Reference Panel as well as its subsets, generated according to the continent of origin (African (87 individuals), Asian (106 individuals) and European (77 individuals) subsets). These target sets (i.e. Global, African, Asian, and European) were then used to impute to WGS level using the Global Reference Panel. **Figure S30.** Dosage R^2^ for all imputed variants and for functional variants for the SNP50V3 array when using the Global Reference Panel and four target sets (i.e., Global, African, Asian and European). Dosage R^2^ for all imputed variants and for functional variants (as annotated by the Ensembl VEP software (LOW, MODERATE and HIGH)) when imputing from the SNP50V3 array to WGS level. The target sets were created by retaining only the WGS genotypes that overlapped with the variants of the SNP50V3 array, from the Global Reference Panel as well as its subsets, generated according to the continent of origin (African (87 individuals), Asian (106 individuals) and European (77 individuals) subsets). These target sets (i.e. Global, African, Asian, and European) were then used to impute to WGS level using the Global Reference Panel. **Figure S31.** Dosage R^2^ for all imputed variants and for functional variants for the ANGGS array when using the Global Reference Panel and four target sets (i.e., Global, African, Asian and European). Dosage R^2^ for all imputed variants and for functional variants (as annotated by the Ensembl VEP software (LOW, MODERATE and HIGH)) when imputing from the ANGGS array to WGS level. The target sets were created by retaining only the WGS genotypes that overlapped with the variants of the ANGGS array, from the Global Reference Panel as well as its subsets, generated according to the continent of origin (African (87 individuals), Asian (106 individuals) and European (77 individuals) subsets). These target sets (i.e. Global, African, Asian, and European) were then used to impute to WGS level using the Global Reference Panel. **Figure S32.** Dosage R^2^ for all imputed variants and for functional variants for the BOVG50V1 array when using the Global Reference Panel and four target sets (i.e., Global, African, Asian and European). Dosage R^2^ for all imputed variants and for functional variants (as annotated by the Ensembl VEP software (LOW, MODERATE and HIGH)) when imputing from the BOVG50V1 array to WGS level. The target sets were created by retaining only the WGS genotypes that overlapped with the variants of the BOVG50V1 array, from the Global Reference Panel as well as its subsets, generated according to the continent of origin (African (87 individuals), Asian (106 individuals) and European (77 individuals) subsets). These target sets (i.e. Global, African, Asian, and European) were then used to impute to WGS level using the Global Reference Panel. **Figure S33.** Dosage R^2^ for all imputed variants and for functional variants for the GGPIND35 array when using the Global Reference Panel and four target sets (i.e., Global, African, Asian and European). Dosage R^2^ for all imputed variants and for functional variants (as annotated by the Ensembl VEP software (LOW, MODERATE and HIGH)) when imputing from the GGPIND35 array to WGS level. The target sets were created by retaining only the WGS genotypes that overlapped with the variants of the GGPIND35 array, from the Global Reference Panel as well as its subsets, generated according to the continent of origin (African (87 individuals), Asian (106 individuals) and European (77 individuals) subsets). These target sets (i.e. Global, African, Asian, and European) were then used to impute to WGS level using the Global Reference Panel. **Figure S34.** Dosage R^2^ for all imputed variants and for functional variants for the GGPLDV4 array when using the Global Reference Panel and four target sets (i.e., Global, African, Asian and European). Dosage R^2^ for all imputed variants and for functional variants (as annotated by the Ensembl VEP software (LOW, MODERATE and HIGH)) when imputing from the GGPLDV4 array to WGS level. The target sets were created by retaining only the WGS genotypes that overlapped with the variants of the GGPLDV4 array, from the Global Reference Panel as well as its subsets, generated according to the continent of origin (African (87 individuals), Asian (106 individuals) and European (77 individuals) subsets). These target sets (i.e. Global, African, Asian, and European) were then used to impute to WGS level using the Global Reference Panel. **Figure S35.** Dosage R^2^ for all imputed variants and for functional variants for the GGPLDV3 array when using the Global Reference Panel and four target sets (i.e., Global, African, Asian and European). Dosage R^2^ for all imputed variants and for functional variants (as annotated by the Ensembl VEP software (LOW, MODERATE and HIGH)) when imputing from the GGPLDV3 array to WGS level. The target sets were created by retaining only the WGS genotypes that overlapped with the variants of the GGPLDV3 array, from the Global Reference Panel as well as its subsets, generated according to the continent of origin (African (87 individuals), Asian (106 individuals) and European (77 individuals) subsets). These target sets (i.e. Global, African, Asian, and European) were then used to impute to WGS level using the Global Reference Panel.

**Additional file 11: Figure S36.** Dosage R^2^ for all imputed indels and for functional indels for the HD array when using the Global Reference Panel and four target sets (i.e., Global, African, Asian and European). Dosage R^2^ for all imputed variants and for functional indels (as annotated by the Ensembl VEP software (LOW, MODERATE and HIGH)) when imputing from the HD array to WGS level. The target sets were created by retaining only the WGS genotypes that overlapped with the variants of the HD array, from the Global Reference Panel as well as its subsets, generated according to the continent of origin (African (87 individuals), Asian (106 individuals) and European (77 individuals) subsets). These target sets (i.e. Global, African, Asian, and European) were then used to impute to WGS level using the Global Reference Panel. **Figure S37.** Dosage R^2^ for all imputed indels and for functional indels for the BOS1 array when using the Global Reference Panel and four target sets (i.e., Global, African, Asian and European). Dosage R^2^ for all imputed variants and for functional indels (as annotated by the Ensembl VEP software (LOW, MODERATE and HIGH)) when imputing from the BOS1 array to WGS level. The target sets were created by retaining only the WGS genotypes that overlapped with the variants of the BOS1 array, from the Global Reference Panel as well as its subsets, generated according to the continent of origin (African (87 individuals), Asian (106 individuals) and European (77 individuals) subsets). These target sets (i.e. Global, African, Asian, and European) were then used to impute to WGS level using the Global Reference Panel. **Figure S38.** Dosage R^2^ for all imputed indels and for functional indels for the GGPF250 array when using the Global Reference Panel and four target sets (i.e., Global, African, Asian and European). Dosage R^2^ for all imputed variants and for functional indels (as annotated by the Ensembl VEP software (LOW, MODERATE and HIGH)) when imputing from theGGPF250 array to WGS level. The target sets were created by retaining only the WGS genotypes that overlapped with the variants of the GGPF250 array, from the Global Reference Panel as well as its subsets, generated according to the continent of origin (African (87 individuals), Asian (106 individuals) and European (77 individuals) subsets). These target sets (i.e. Global, African, Asian, and European) were then used to impute to WGS level using the Global Reference Panel. **Figure S39.** Dosage R^2^ for all imputed indels and for functional indels for the GGPHDV3 array when using the Global Reference Panel and four target sets (i.e., Global, African, Asian and European). Dosage R^2^ for all imputed variants and for functional indels (as annotated by the Ensembl VEP software (LOW, MODERATE and HIGH)) when imputing from theGGPHDV3 array to WGS level. The target sets were created by retaining only the WGS genotypes that overlapped with the variants of the GGPHDV3 array, from the Global Reference Panel as well as its subsets, generated according to the continent of origin (African (87 individuals), Asian (106 individuals) and European (77 individuals) subsets). These target sets (i.e. Global, African, Asian, and European) were then used to impute to WGS level using the Global Reference Panel. **Figure S40.** Dosage R^2^ for all imputed indels and for functional indels for the GGP90KT array when using the Global Reference Panel and four target sets (i.e., Global, African, Asian and European). Dosage R^2^ for all imputed variants and for functional indels (as annotated by the Ensembl VEP software (LOW, MODERATE and HIGH)) when imputing from theGGP90KT array to WGS level. The target sets were created by retaining only the WGS genotypes that overlapped with the variants of the GGP90KT array, from the Global Reference Panel as well as its subsets, generated according to the continent of origin (African (87 individuals), Asian (106 individuals) and European (77 individuals) subsets). These target sets (i.e. Global, African, Asian, and European) were then used to impute to WGS level using the Global Reference Panel. **Figure S41.** Dosage R^2^ for all imputed indels and for functional indels for the IND90KH array when using the Global Reference Panel and four target sets (i.e., Global, African, Asian and European). Dosage R^2^ for all imputed variants and for functional indels (as annotated by the Ensembl VEP software (LOW, MODERATE and HIGH)) when imputing from theIND90KH array to WGS level. The target sets were created by retaining only the WGS genotypes that overlapped with the variants of the IND90KH array, from the Global Reference Panel as well as its subsets, generated according to the continent of origin (African (87 individuals), Asian (106 individuals) and European (77 individuals) subsets). These target sets (i.e. Global, African, Asian, and European) were then used to impute to WGS level using the Global Reference Panel. **Figure S42.** Dosage R^2^ for all imputed indels and for functional indels for the ZMD2 array when using the Global Reference Panel and four target sets (i.e., Global, African, Asian and European). Dosage R^2^ for all imputed variants and for functional indels (as annotated by the Ensembl VEP software (LOW, MODERATE and HIGH)) when imputing from the ZMD2 array to WGS level. The target sets were created by retaining only the WGS genotypes that overlapped with the variants of the ZMD2 array, from the Global Reference Panel as well as its subsets, generated according to the continent of origin (African (87 individuals), Asian (106 individuals) and European (77 individuals) subsets). These target sets (i.e. Global, African, Asian, and European) were then used to impute to WGS level using the Global Reference Panel. **Figure S43.** Dosage R^2^ for all imputed indels and for functional indels for the ZOETIS1 array when using the Global Reference Panel and four target sets (i.e., Global, African, Asian and European). Dosage R^2^ for all imputed variants and for functional indels (as annotated by the Ensembl VEP software (LOW, MODERATE and HIGH)) when imputing from theZOETIS1 array to WGS level. The target sets were created by retaining only the WGS genotypes that overlapped with the variants of the ZOETIS1 array, from the Global Reference Panel as well as its subsets, generated according to the continent of origin (African (87 individuals), Asian (106 individuals) and European (77 individuals) subsets). These target sets (i.e. Global, African, Asian, and European) were then used to impute to WGS level using the Global Reference Panel. **Figure S44.** Dosage R^2^ for all imputed indels and for functional indels for the BOVMD array when using the Global Reference Panel and four target sets (i.e., Global, African, Asian and European). Dosage R^2^ for all imputed variants and for functional indels (as annotated by the Ensembl VEP software (LOW, MODERATE and HIGH)) when imputing from the BOVMD array to WGS level. The target sets were created by retaining only the WGS genotypes that overlapped with the variants of the BOVMD array, from the Global Reference Panel as well as its subsets, generated according to the continent of origin (African (87 individuals), Asian (106 individuals) and European (77 individuals) subsets). These target sets (i.e. Global, African, Asian, and European) were then used to impute to WGS level using the Global Reference Panel. **Figure S45.** Dosage R^2^ for all imputed indels and for functional indels for the IDBV3 array when using the Global Reference Panel and four target sets (i.e., Global, African, Asian and European). Dosage R^2^ for all imputed variants and for functional indels (as annotated by the Ensembl VEP software (LOW, MODERATE and HIGH)) when imputing from the IDBV3 array to WGS level. The target sets were created by retaining only the WGS genotypes that overlapped with the variants of the IDBV3 array, from the Global Reference Panel as well as its subsets, generated according to the continent of origin (African (87 individuals), Asian (106 individuals) and European (77 individuals) subsets). These target sets (i.e. Global, African, Asian, and European) were then used to impute to WGS level using the Global Reference Panel. **Figure S46.** Dosage R^2^ for all imputed indels and for functional indels for the SNP50V3 array when using the Global Reference Panel and four target sets (i.e., Global, African, Asian and European). Dosage R^2^ for all imputed variants and for functional indels (as annotated by the Ensembl VEP software (LOW, MODERATE and HIGH)) when imputing from the SNP50V3 array to WGS level. The target sets were created by retaining only the WGS genotypes that overlapped with the variants of the SNP50V3 array, from the Global Reference Panel as well as its subsets, generated according to the continent of origin (African (87 individuals), Asian (106 individuals) and European (77 individuals) subsets). These target sets (i.e. Global, African, Asian, and European) were then used to impute to WGS level using the Global Reference Panel. **Figure S47.** Dosage R^2^ for all imputed indels and for functional indels for the ANGGS array when using the Global Reference Panel and four target sets (i.e., Global, African, Asian and European). Dosage R^2^ for all imputed variants and for functional indels (as annotated by the Ensembl VEP software (LOW, MODERATE and HIGH)) when imputing from theANGGS array to WGS level. The target sets were created by retaining only the WGS genotypes that overlapped with the variants of the ANGGS array, from the Global Reference Panel as well as its subsets, generated according to the continent of origin (African (87 individuals), Asian (106 individuals) and European (77 individuals) subsets). These target sets (i.e. Global, African, Asian, and European) were then used to impute to WGS level using the Global Reference Panel. **Figure S48.** Dosage R^2^ for all imputed indels and for functional indels for the BOVG50V1 array when using the Global Reference Panel and four target sets (i.e., Global, African, Asian and European). Dosage R^2^ for all imputed variants and for functional indels (as annotated by the Ensembl VEP software (LOW, MODERATE and HIGH)) when imputing from the BOVG50V1 array to WGS level. The target sets were created by retaining only the WGS genotypes that overlapped with the variants of the BOVG50V1 array, from the Global Reference Panel as well as its subsets, generated according to the continent of origin (African (87 individuals), Asian (106 individuals) and European (77 individuals) subsets). These target sets (i.e. Global, African, Asian, and European) were then used to impute to WGS level using the Global Reference Panel. **Figure S49.** Dosage R^2^ for all imputed indels and for functional indels for the GGPIND35 array when using the Global Reference Panel and four target sets (i.e., Global, African, Asian and European). Dosage R^2^ for all imputed variants and for functional indels (as annotated by the Ensembl VEP software (LOW, MODERATE and HIGH)) when imputing from the GGPIND35 array to WGS level. The target sets were created by retaining only the WGS genotypes that overlapped with the variants of the GGPIND35 array, from the Global Reference Panel as well as its subsets, generated according to the continent of origin (African (87 individuals), Asian (106 individuals) and European (77 individuals) subsets). These target sets (i.e. Global, African, Asian, and European) were then used to impute to WGS level using the Global Reference Panel. **Figure S50.** Dosage R^2^ for all imputed indels and for functional indels for the GGPLDV4 array when using the Global Reference Panel and four target sets (i.e., Global, African, Asian and European). Dosage R^2^ for all imputed variants and for functional indels (as annotated by the Ensembl VEP software (LOW, MODERATE and HIGH)) when imputing from the GGPLDV4 array to WGS level. The target sets were created by retaining only the WGS genotypes that overlapped with the variants of the GGPLDV4 array, from the Global Reference Panel as well as its subsets, generated according to the continent of origin (African (87 individuals), Asian (106 individuals) and European (77 individuals) subsets). These target sets (i.e. Global, African, Asian, and European) were then used to impute to WGS level using the Global Reference Panel. **Figure S51.** Dosage R^2^ for all imputed indels and for functional indels for the GGPLDV3 array when using the Global Reference Panel and four target sets (i.e., Global, African, Asian and European). Dosage R^2^ for all imputed variants and for functional indels (as annotated by the Ensembl VEP software (LOW, MODERATE and HIGH)) when imputing from the GGPLDV3 array to WGS level. The target sets were created by retaining only the WGS genotypes that overlapped with the variants of the GGPLDV3 array, from the Global Reference Panel as well as its subsets, generated according to the continent of origin (African (87 individuals), Asian (106 individuals) and European (77 individuals) subsets). These target sets (i.e. Global, African, Asian, and European) were then used to impute to WGS level using the Global Reference Panel.

**Additional file 12: Figure S52.** Plot for principal component (PC) 1 and PC2 for the individuals collected across four African countries, genotyped with the Geneseek 50 k array. Plot for principal component (PC) 1 and PC2 for the individuals collected across four African countries (namely Tanzania, Ghana, Nigeria, and Burkina Faso), genotyped with the Geneseek 50 k array. Only individuals unrelated were used (relatedness value from vcftools -relatedness2 > 0.0625). Coloured by country. **Figure S53.** Plot for principal component (PC) 1 and PC2 for the individuals collected across four African countries, genotyped with the Illumina HD array. Plot for principal component (PC) 1 and PC2 for the individuals collected across four African countries (namely Tanzania, Ghana, Nigeria, and Burkina Faso), genotyped with the Illumina HD array. Only individuals unrelated were used (relatedness value from vcftools -relatedness2 > 0.0625). Coloured by country. **Figure S54.** Plot for principal component (PC) 1 and PC2 for the combined data of 2,481 individuals genotyped with the Illumina HD array. Plot for principal component (PC) 1 and PC2 for combined data of 2,481 individuals, genotyped with the Illumina HD array. Only individuals unrelated were used (relatedness value from vcftools –relatedness2 > 0.0625). Coloured by population, as reported in Additional file 4: Table S3.

## Data Availability

Some of the sequence data used in this study are from public databases, as detailed in Additional file 1: Tables S1 and S2. Whole-genome sequence variants (i.e., 35,842,537 SNPs) from the 120 samples of Boran, N'Dama and Holstein cattle (i.e., 40 samples per breed) and raw Illumina HD genotypes (i.e., 777,962 SNPs) mapped to the bovine UMD3.1 genome assembly for 3092 cattle from the four African countries (namely Burkina Faso, Ghana, Nigeria, and Tanzania) have been uploaded on Zenodo with https://doi.org/10.5281/zenodo.6855979 and https://doi.org/10.5281/zenodo.6791394, respectively.
