# Supplementary material for: Assessment of genotyping array performance for genome-wide association studies and imputation in African cattle
Source: Genet Sel Evol. 2022 Sep 4;54:58. doi: 10.1186/s12711-022-00751-5 (PMC9441065; doi:10.1186/s12711-022-00751-5)
Supplement: Supplementary file 1 — Additional file 1: Table S1. Populations with whole-genome sequence data. Number of animals, with whole-genome sequence data per breed/population used in this study, indicating sources and/or project accession numbers. Table S2. Accession numbers of the Boran samples used in this study, which are publicly available. [file 12711_2022_751_MOESM1_ESM.docx]

**Additional file 1 Table S1**

| **Populations (number of samples)** | **Source/Bio project ID** |
| --- | --- |
| Ethiopian Boran (24) and Kenya Boran (16) | Unpublished data, https://grrfac.ilri.org, Kim et al. (2017), Kim et al. (2020) and Dutta et al. (2020) |
| NDama (40) | Unpublished data, https://grrfac.ilri.org |
| Holstein-Friesian (40) | PRJNA431934, PRJEB27379, PRJNA477833, PRJNA176557 and PRJNA478565 |
| Angus (4), Ankole (8), Baoule (7), Bohai Black Cattle (5), Boran (15), Brahman (5), Brown Swiss (4), Chaidamu Yellow Cattle (2), Charolais (5), Dabieshan Cattle (2), Dianzhong Cattle (6), Djakkore (7), Gir (4), Gourounsi (3), Guangfeng Cattle (4), Hanwoo (4), Hariana (1), Hereford (5), Holstein-Friesian (33), IRBT (5), Jersey (4), Jian Cattle (4), Jiaxian Red Cattle (5), Jinjiang Cattle (4), Kazakh Cattle (8), Kenana (9), Leiqiong Cattle (3), Limousine (4), Lingnan Cattle (8), Luxi Cattle (4), Mongolian Cattle (6), Muturu (7), Nanyang Cattle (4), NDama (10), Nelore (2), Norwegian Red (1), Ogaden (9), Piedmontese (5), Romagnola (4), Sahiwal (1), Scottish Highland (2), Simmenthal (5), Swedish Red (1), Tharparkar (1), Tibetan Yellow Cattle (8), AnkolexZebu (2), Wandong Cattle (2), Wannan Cattle (5), Weining Cattle (5), Wenshan Cattle (7), Xuanhan Cattle (5), Yanbian Cattle (1), Zaobei Cattle (4), Zebu Gobra (6), Zebu Maure (4) | Dutta et al. (2020) |

**Additional file 1 Table S2**

| **Samples** | **Accession** | **BioProject** |
| --- | --- | --- |
| BORETHF000000000001 | SRS7201966 | PRJNA574857 |
| BORETHF000000000002 | SRX8946649 | PRJNA574857 |
| BORETHF000000000003 | SRX8946650 | PRJNA574857 |
| BORETHF000000000004 | SRX8946651 | PRJNA574857 |
| BORETHF000000000005 | SRX8946652 | PRJNA574857 |
| BORETHF000000000007 | SRX8946653 | PRJNA574857 |
| BORETHF000000000010 | SRX8946654 | PRJNA574857 |
| BORETHM000000000012 | SRX8946655 | PRJNA574857 |
| BORETHM000000000013 | SRX8946656 | PRJNA574857 |
| BORETHM000000000015 | SRX8946658 | PRJNA574857 |
| BORKENX000000002684 | ERS4802949 | PRJEB39210 |
| BORKENX000000002754 | ERS4802932 | PRJEB39210 |
| BORKENX000000002794 | ERS4802957 | PRJEB39210 |
| BORKENX000000003265 | ERS4802953 | PRJEB39210 |
| BORKENX000000003321 | ERS4802922 | PRJEB39210 |
| BORKENX000000003324 | ERS4802956 | PRJEB39210 |
| BORKENX000000000375 | SRR3546727 | PRJNA312138 |
| BORKENX000000000392 | SRR3546728 | PRJNA312138 |
| BORKENX000000000563 | SRR3546787 | PRJNA312138 |
| BORKENX000000000672 | SRR3508267 | PRJNA312138 |
