# Supplementary figures and images for: Assessment of genotyping array performance for genome-wide association studies and imputation in African cattle
Source: Genet Sel Evol. 2022 Sep 4;54:58. doi: 10.1186/s12711-022-00751-5 (PMC9441065; doi:10.1186/s12711-022-00751-5)

## Additional file 2 Figure S1

**a**

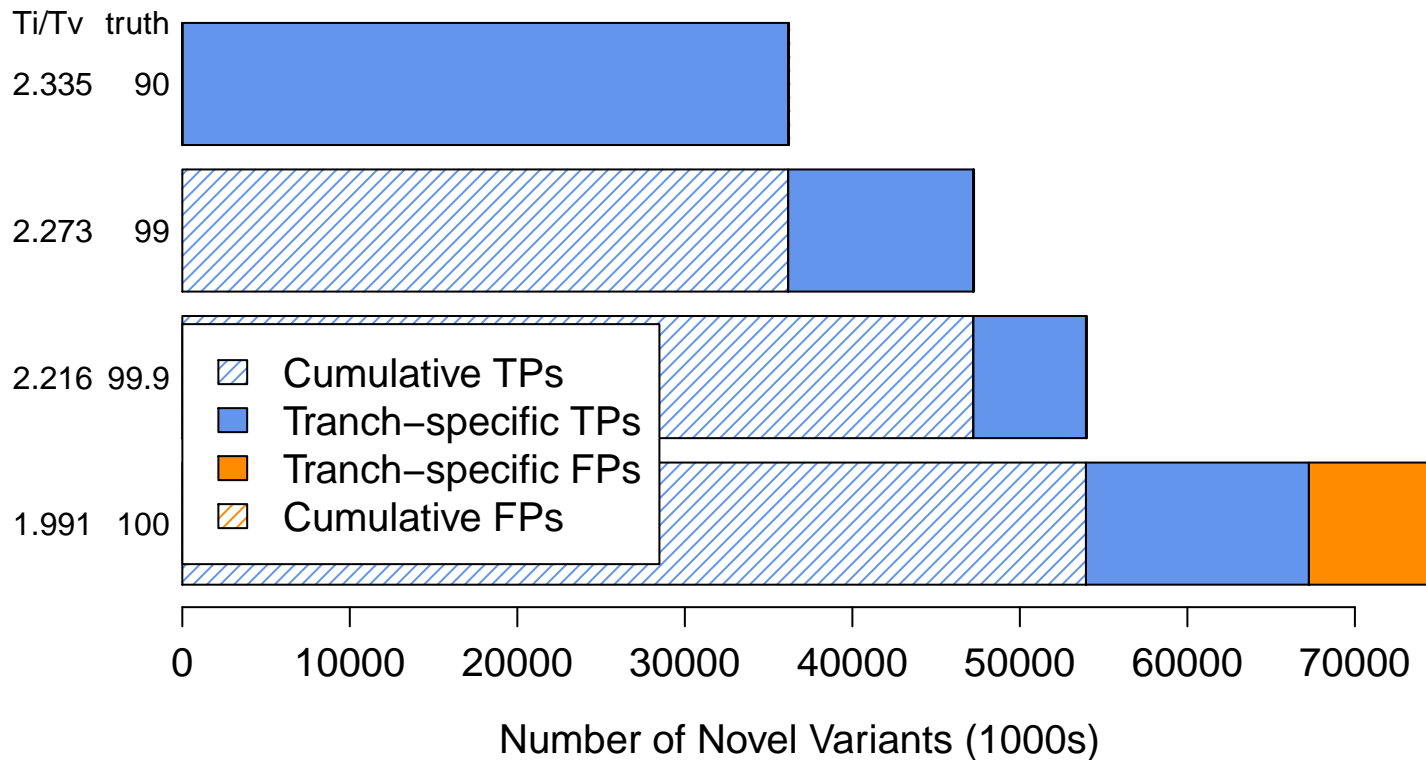

**b**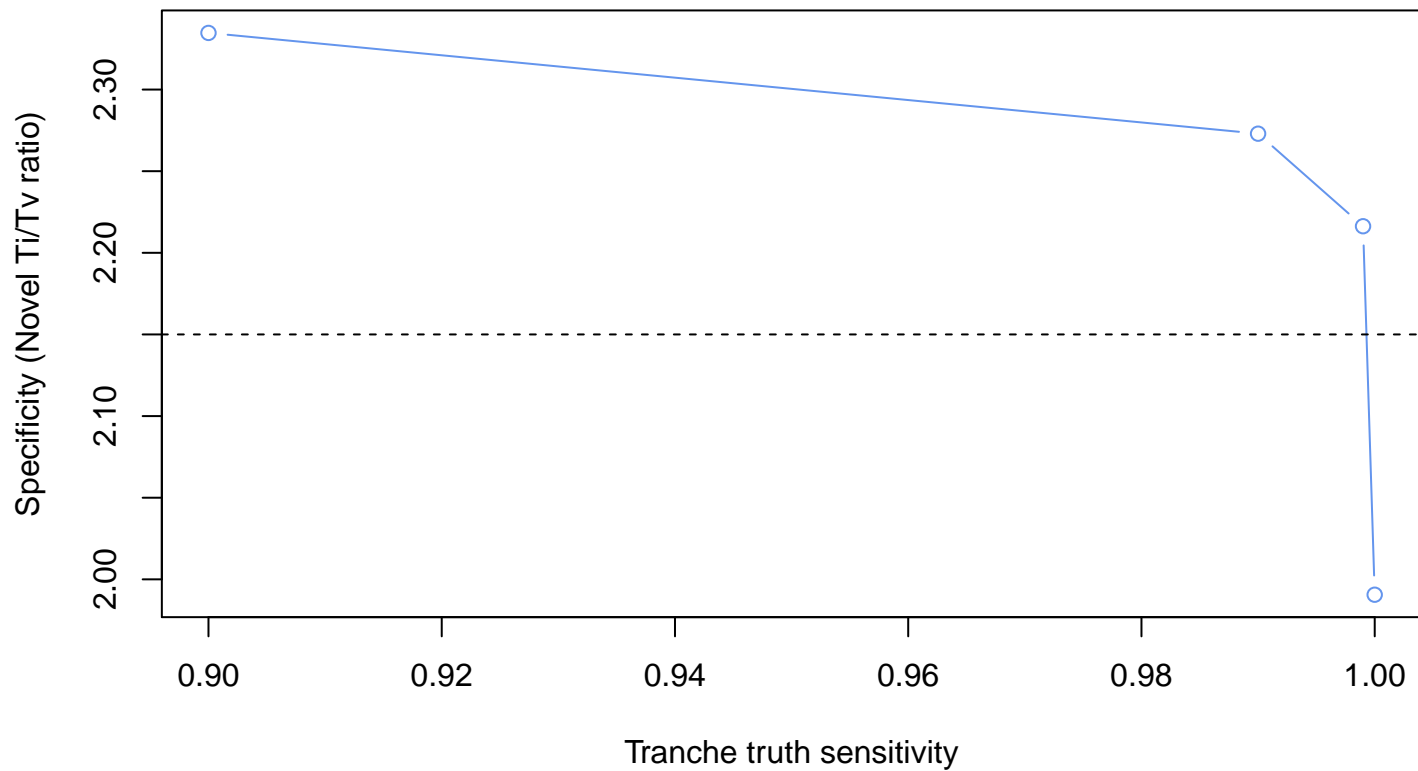

Supplement: Supplementary file 2 — Additional file 2: Figure S1. Variant Quality Score Recalibration for WGS variants using GATK: tranches plot (a) and specificity versus tranche truth sensitivity (b). Quality metrics of the WGS data. Tranche-specific TP are true-positive calls gained when adding a slice to the plate. Cumulative TP are true-positive calls contained in all the slices already added. Thus, this differentiation allows to evaluate how many more TP are gained vs. the additional false positives (FP) that have to be taken on, when going to the next tranche up. The ratio of transition (Ti) to transversion (Tv) SNPs (i.e., Ti/Tv ratio) is a useful diagnostic tool to measure the quality of the WGS data generated. A high Ti/Tv ratio (> 2.0) often indicates a high-accuracy SNP set, whereas a low value (~ 0.5) implies low-quality SNP calling. [file 12711_2022_751_MOESM2_ESM.pdf]

Additional file 3 Figure S2

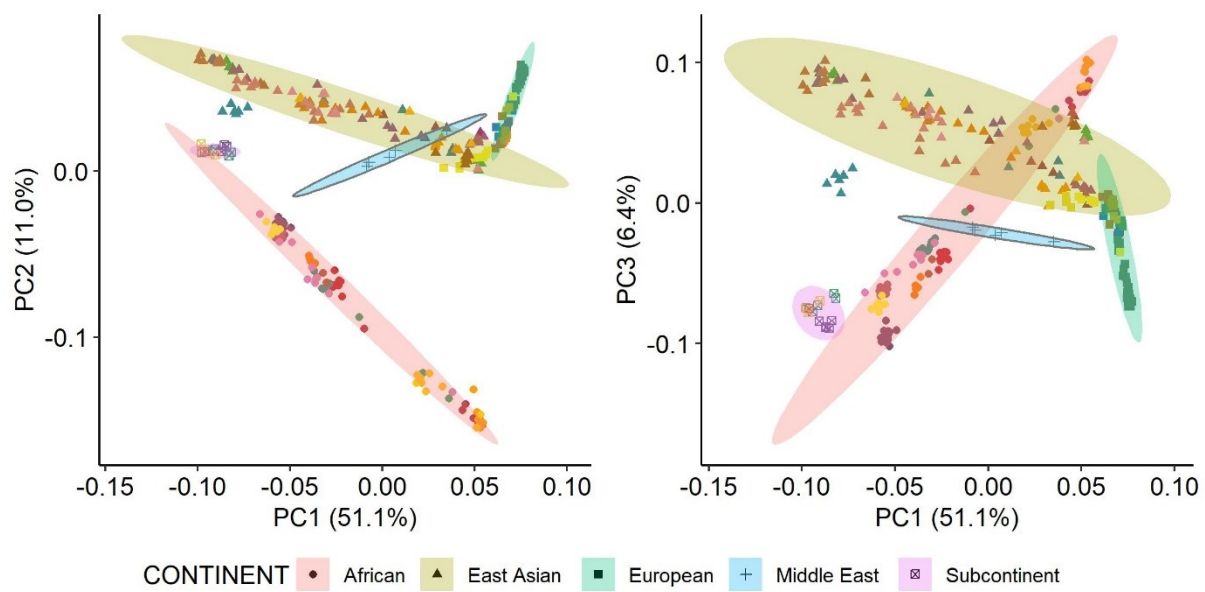

Supplement: Supplementary file 3 — Additional file 3: Figure S2. Principal component plot of the individuals included in the WGS data. Plot for principal component (PC) 1 and PC2 as well as PC1 and PC3 for the 289 distinct individuals included in the WGS data. The data spanned a diverse range of breeds and geographic locations (55 populations, among which 13 European, 12 African, 28 Asian, and 2 Middle Eastern). Coloured by population and location [34]. [file 12711_2022_751_MOESM3_ESM.pdf]

Additional file 6 Figure S3

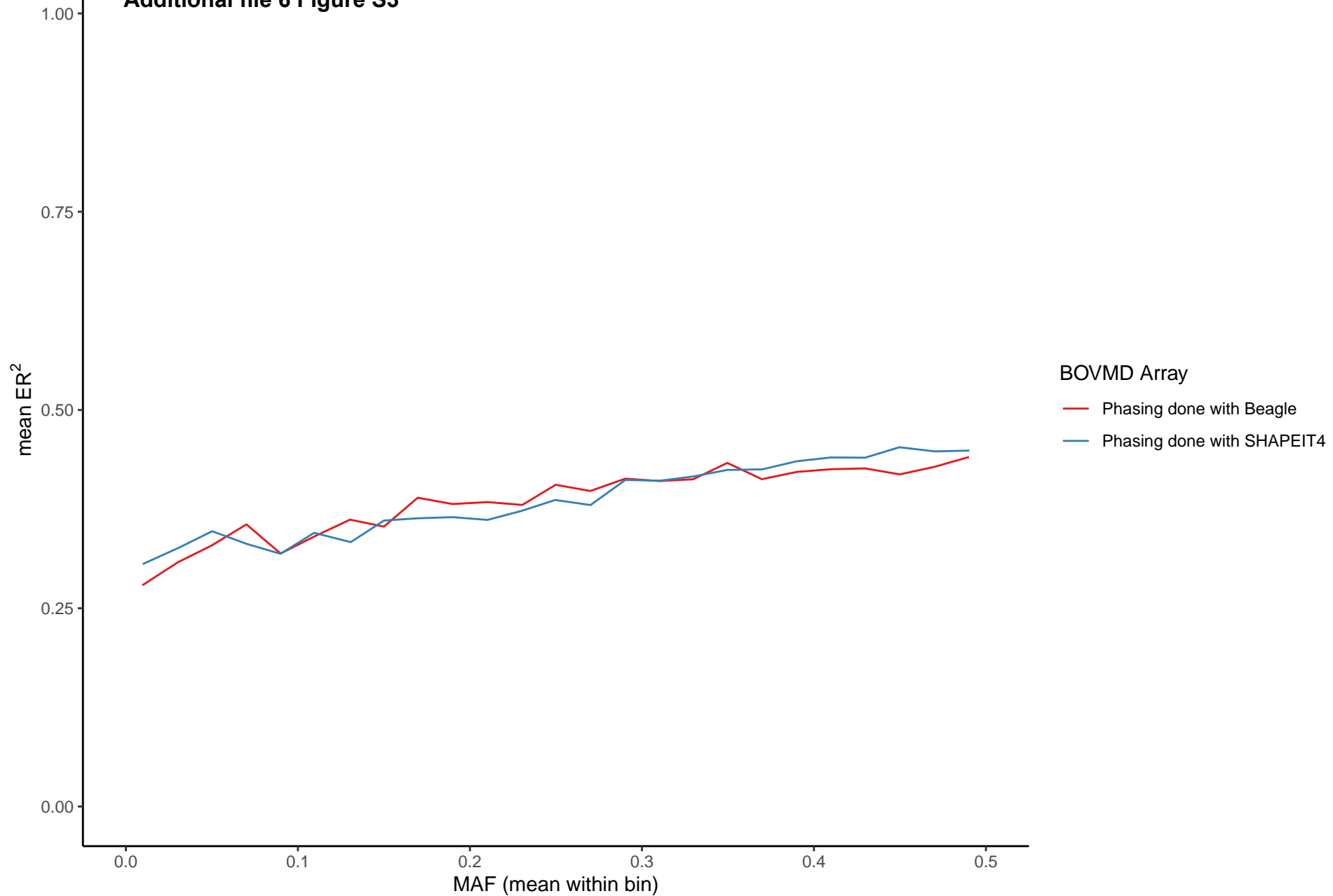

Supplement: Supplementary file 6 — Additional file 6: Figure S3. Comparison between imputation accuracies (ER2) when phasing was done with either BEAGLE or SHAPEIT4. Comparison between imputation accuracies (ER2, as estimated in Minimac4) when phasing was done with either BEAGLE or SHAPEIT4. Since the imputation accuracies were similar, BEAGLE phased data were used for all subsequent analyses. [file 12711_2022_751_MOESM6_ESM.pdf]

Additional file 7 Figure S4

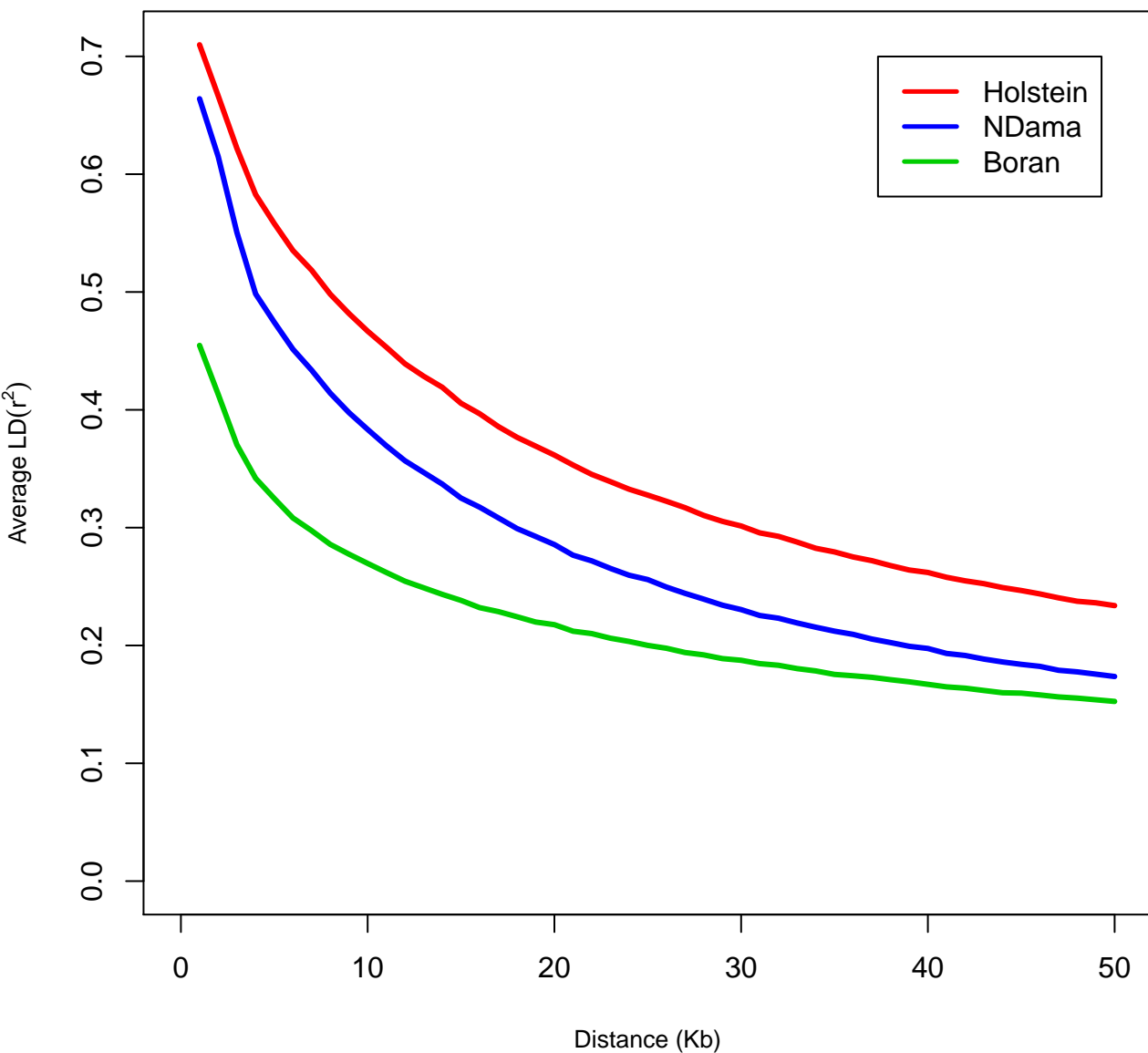

Supplement: Supplementary file 7 — Additional file 7: Figure S4. Linkage disequilibrium (r2) decay in European and African cattle breeds. Comparison of linkage disequilibrium decay in taurine (both European and African) and African indicine breeds. [file 12711_2022_751_MOESM7_ESM.pdf]

**Additional file 8 Figure S5**

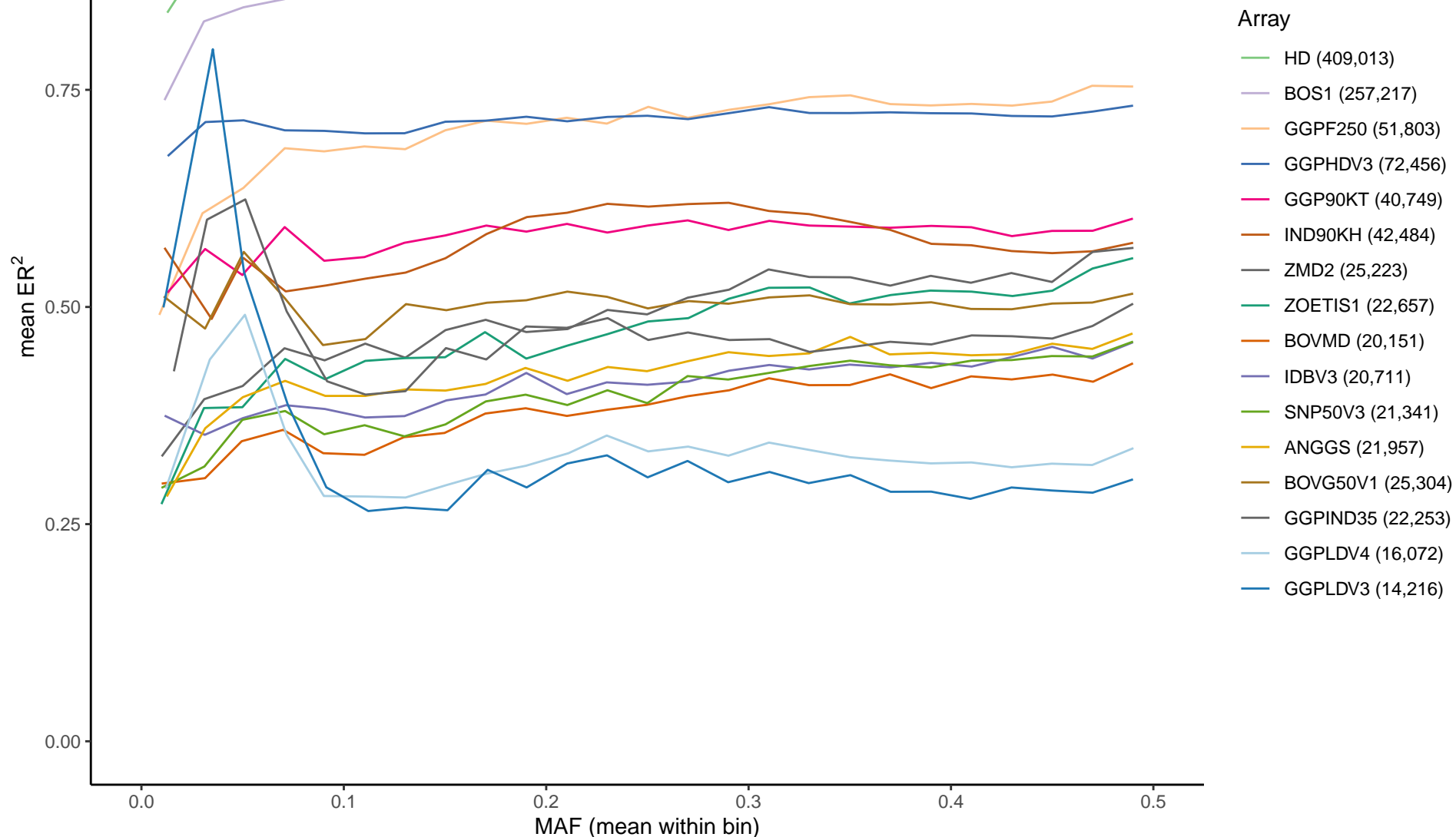

Supplement: Supplementary file 8 — Additional file 8: Figure S5. Imputation accuracy (ER2) with the leave-one-out cross-validation, using 100 of the 289 animals from the WGS data, for all bovine genotyping arrays considered. In this procedure, one individual was removed from the reference panel and its genotypes imputed using the remaining animals as the reference panel. This was repeated for each of the 100 animals, randomly selected from the WGS data and for each array. The results are presented only for 16 arrays (i.e., those retaining more than 10,000 variants after QC), for which imputation was successful. The number of variants retained from the WGS data for each array is between brackets. [file 12711_2022_751_MOESM8_ESM.pdf]

Additional file 12 Figure S52

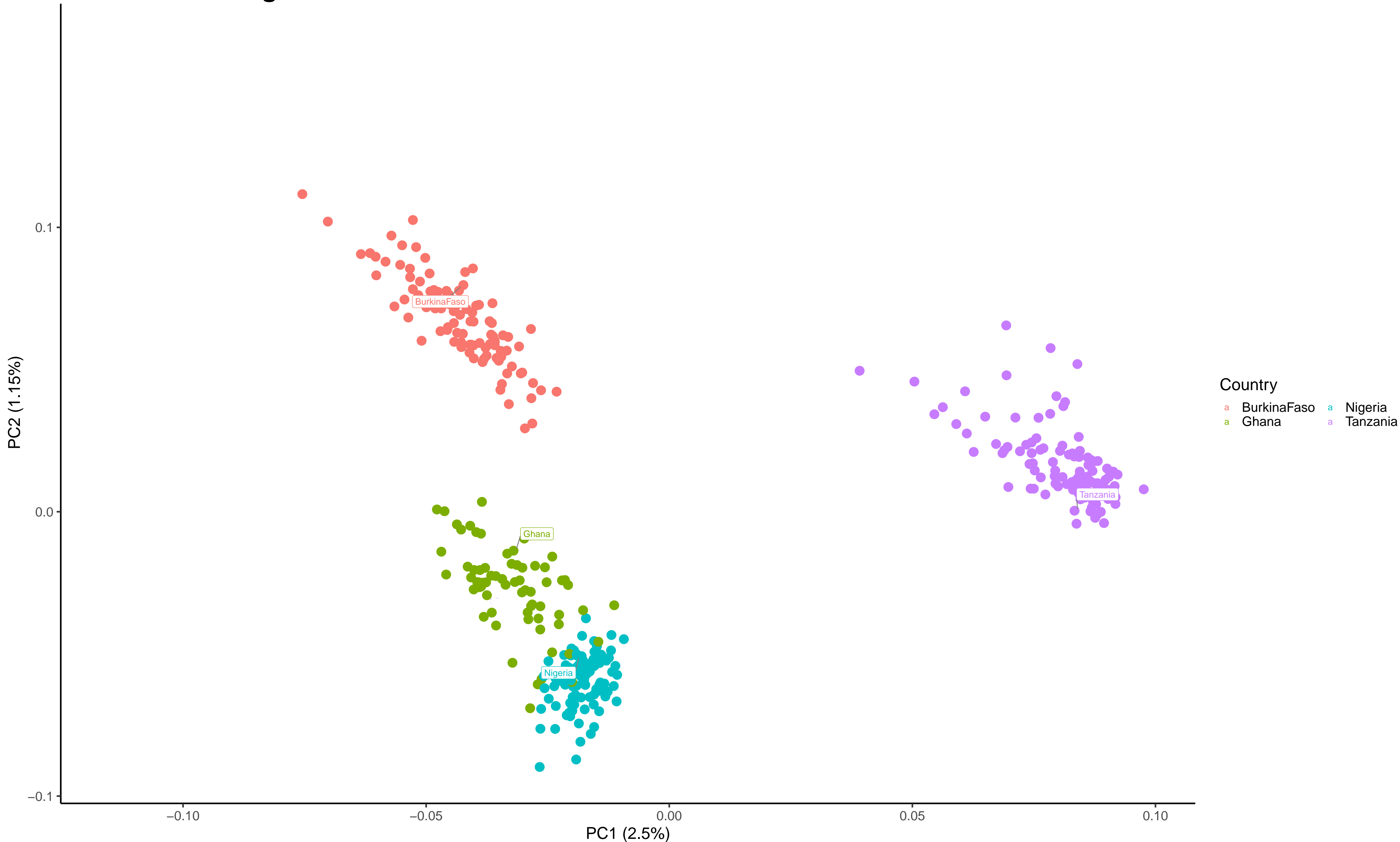

Additional file 12 Figure S53

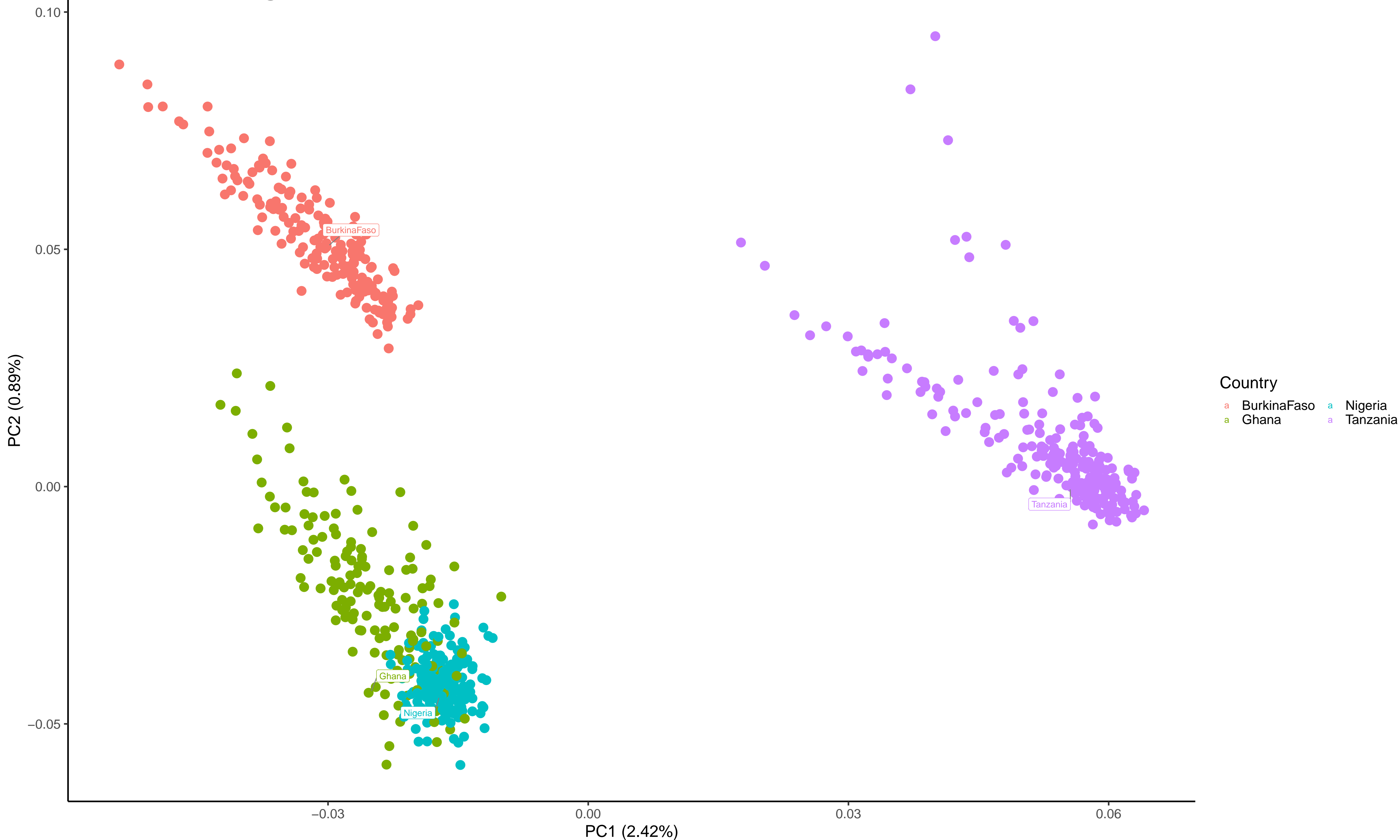

Additional file 12 Figure S54

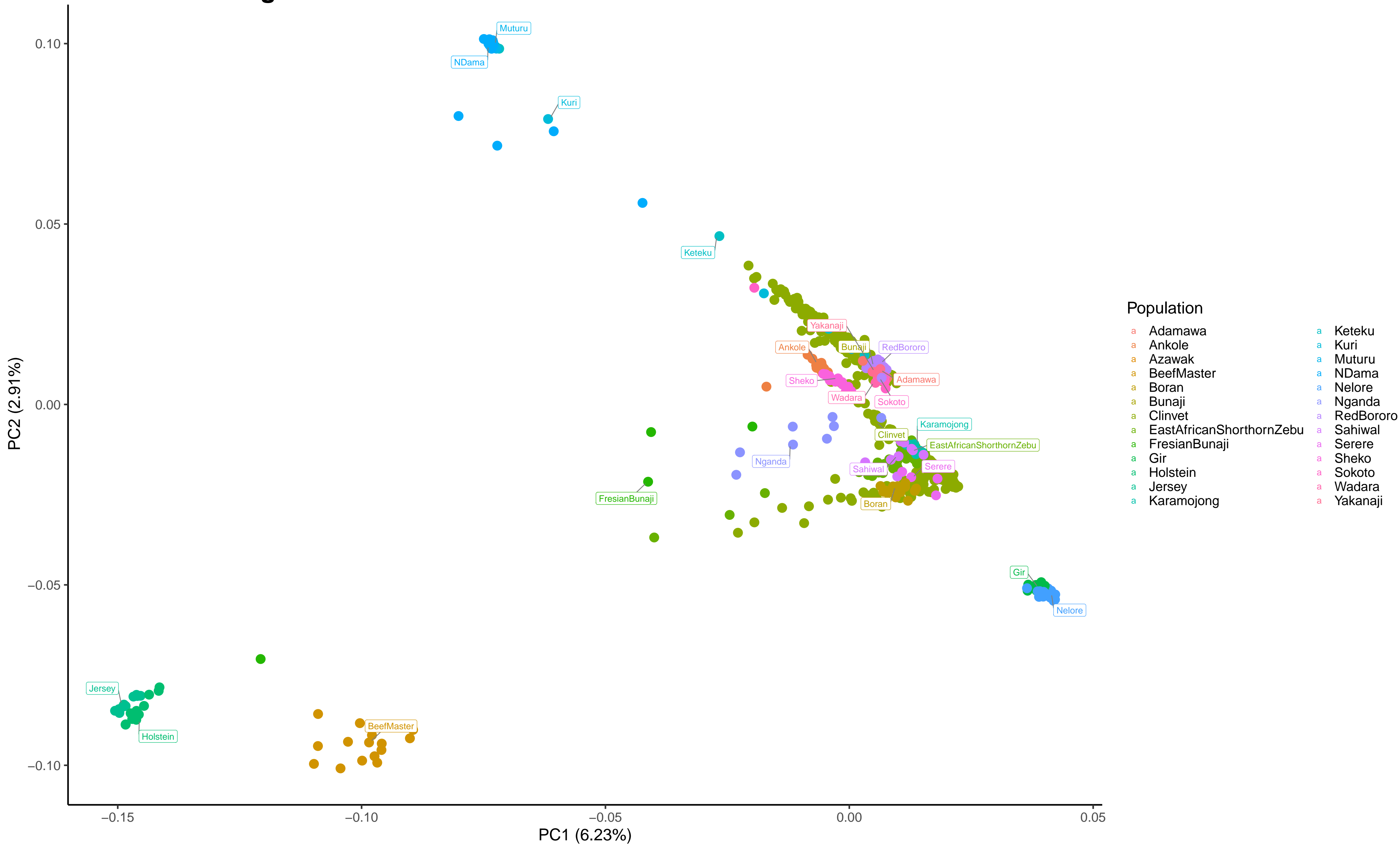

Supplement: Supplementary file 12 — Additional file 12: Figure S52. Plot for principal component (PC) 1 and PC2 for the individuals collected across four African countries, genotyped with the Geneseek 50 k array. Plot for principal component (PC) 1 and PC2 for the individuals collected across four African countries (namely Tanzania, Ghana, Nigeria, and Burkina Faso), genotyped with the Geneseek 50 k array. Only individuals unrelated were used (relatedness value from vcftools -relatedness2 > 0.0625). Coloured by country. Figure S53. Plot for principal component (PC) 1 and PC2 for the individuals collected across four African countries, genotyped with the Illumina HD array. Plot for principal component (PC) 1 and PC2 for the individuals collected across four African countries (namely Tanzania, Ghana, Nigeria, and Burkina Faso), genotyped with the Illumina HD array. Only individuals unrelated were used (relatedness value from vcftools -relatedness2 > 0.0625). Coloured by country. Figure S54. Plot for principal component (PC) 1 and PC2 for the combined data of 2,481 individuals genotyped with the Illumina HD array. Plot for principal component (PC) 1 and PC2 for combined data of 2,481 individuals, genotyped with the Illumina HD array. Only individuals unrelated were used (relatedness value from vcftools –relatedness2 > 0.0625). Coloured by population, as reported in Additional file 4: Table S3. [file 12711_2022_751_MOESM12_ESM.pdf]
