# Supplementary material for: Assessment of genotyping array performance for genome-wide association studies and imputation in African cattle
Source: Genet Sel Evol. 2022 Sep 4;54:58. doi: 10.1186/s12711-022-00751-5 (PMC9441065; doi:10.1186/s12711-022-00751-5)
Supplement: Supplementary file 4 — Additional file 4: Table S3. Populations represented in the combined HD data. Number of animals genotyped with the Illumina HD array per breed/population used in this study, and data source [file 12711_2022_751_MOESM4_ESM.docx]

**Additional file 4 Table S3**

| **Population/s (number of samples)** | **Source** |
| --- | --- |
| Samples collected across four African countries (3092) | Current study |
| Adamawa Gudali (25), Ankole (25), Azawak (2), Bunaji (23), East African Shorthorn Zebu (114), Karamojong Zebu (16), Muturu (12), Ndama (24), Nelore (35), Nganda (23), Red Bororo (22), Serere Zebu (13), Sokoto Gudali (21), Wadara (3), Yakanaji (12) | Bahbahani et al. (2017) |
| BeefMaster (24), Gir (30), Holstein (63), Jersey (36), Sheko (18) | Bovine HapMap et al. (2009) |
| Keteku (13), Kuri (2), Fresian_Bunaji (24), Ndama (23), Sahiwal Zebu (13) | Tijjani (2013) |
| Boran (144) | Wragg et al. (2021) |
