# Supplementary material for: Assessment of genotyping array performance for genome-wide association studies and imputation in African cattle
Source: Genet Sel Evol. 2022 Sep 4;54:58. doi: 10.1186/s12711-022-00751-5 (PMC9441065; doi:10.1186/s12711-022-00751-5)

Additional file 9 Figure S6

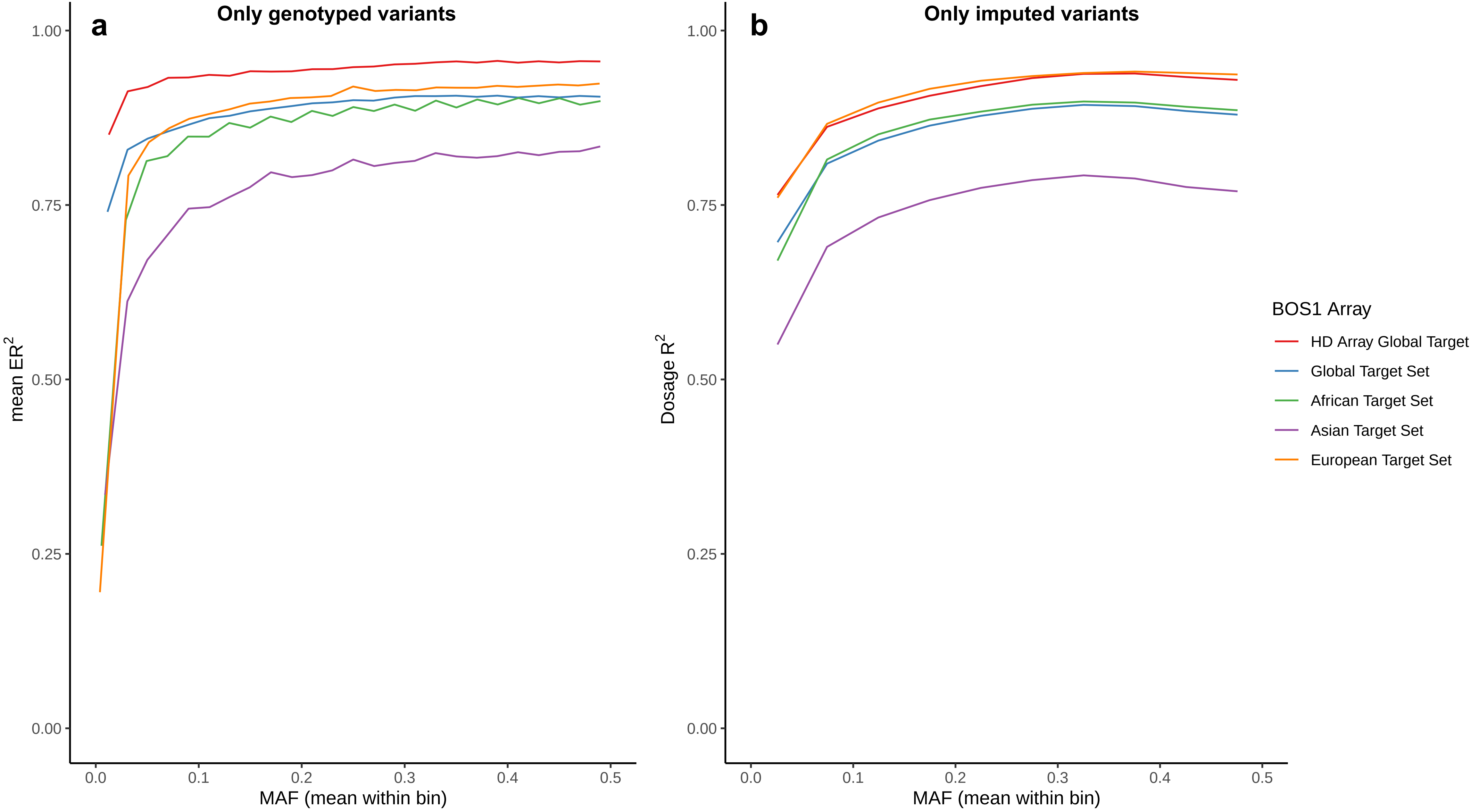

Additional file 9 Figure S7

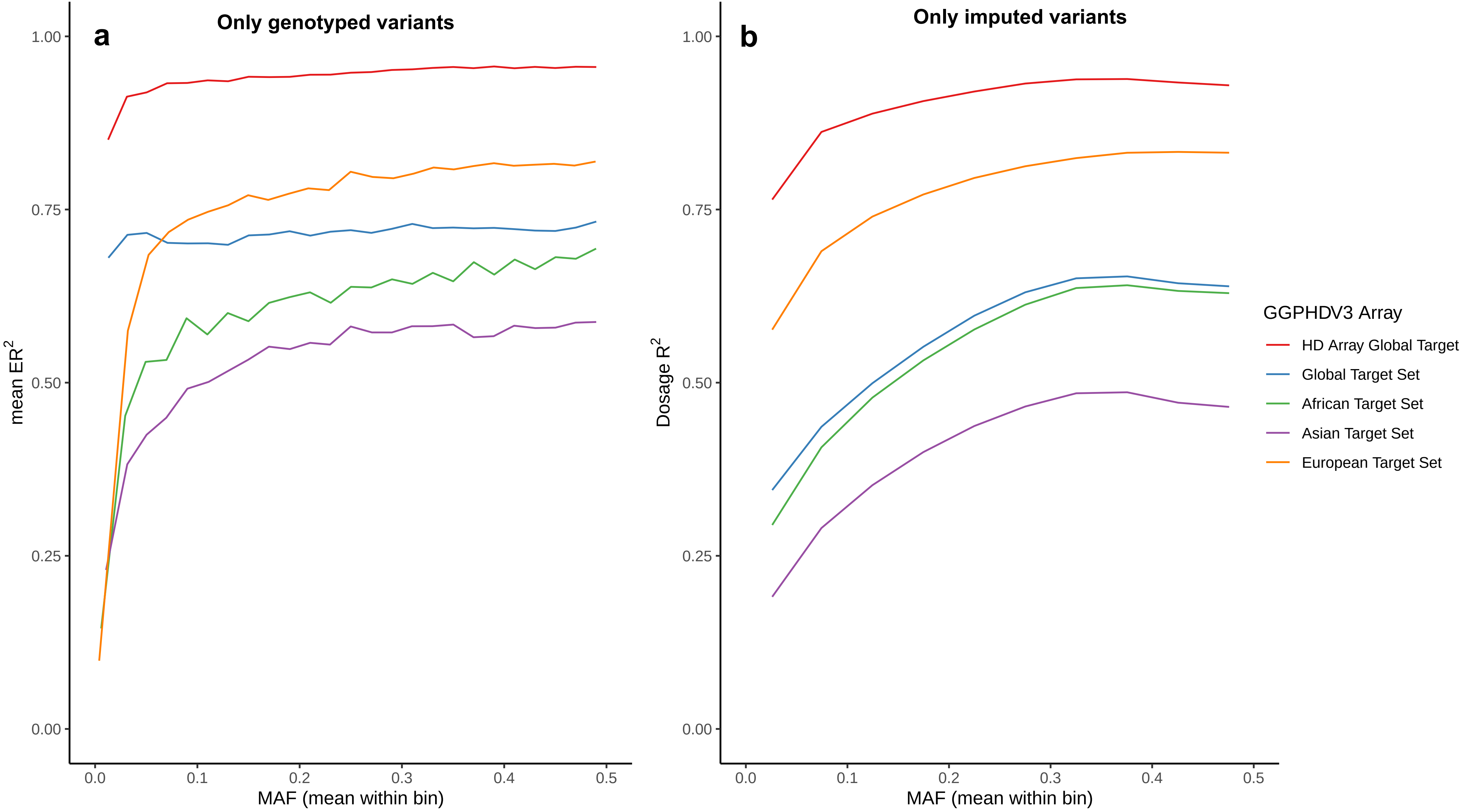

Additional file 9 Figure S8

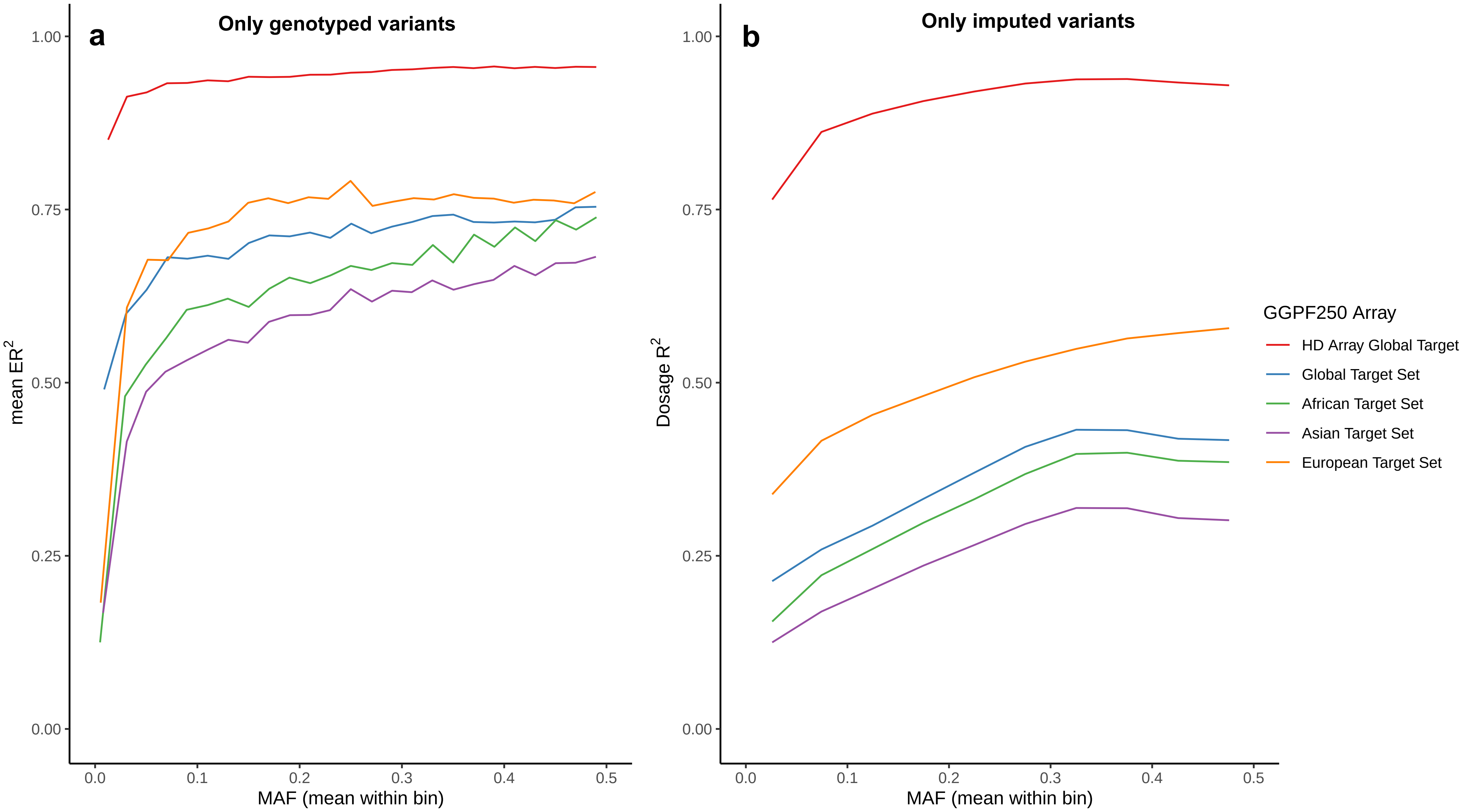

Additional file 9 Figure S9

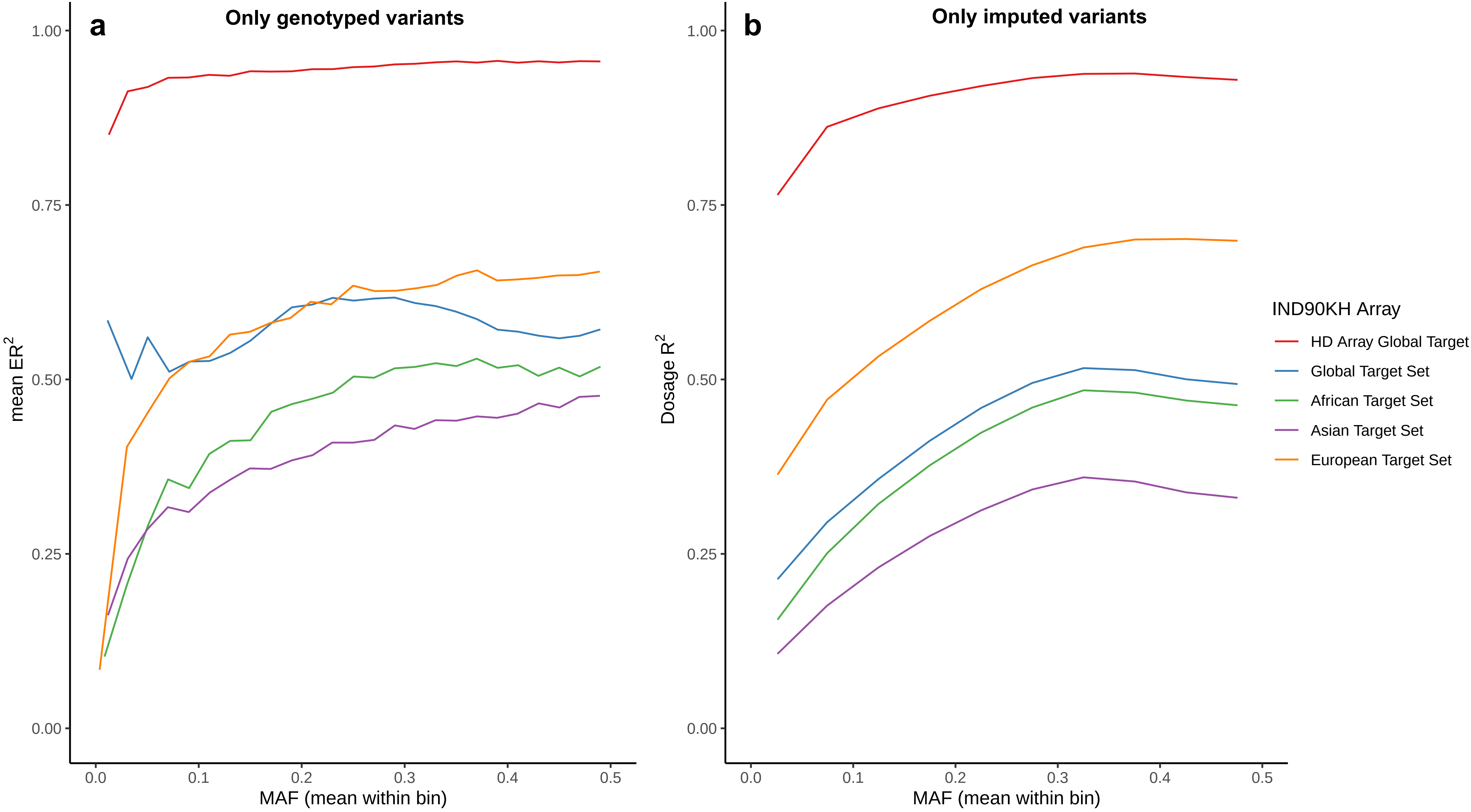

Additional file 9 Figure S10

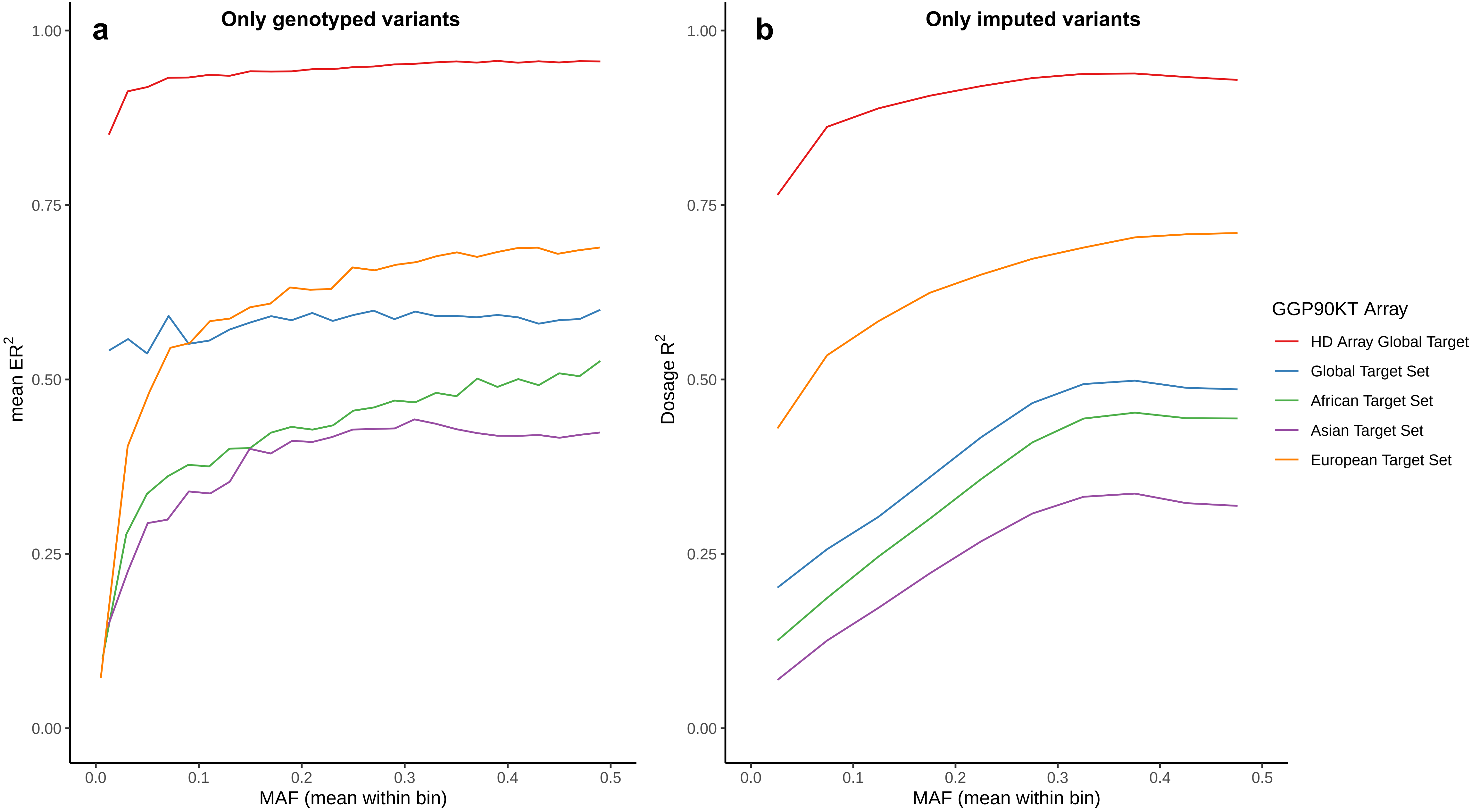

Additional file 9 Figure S11

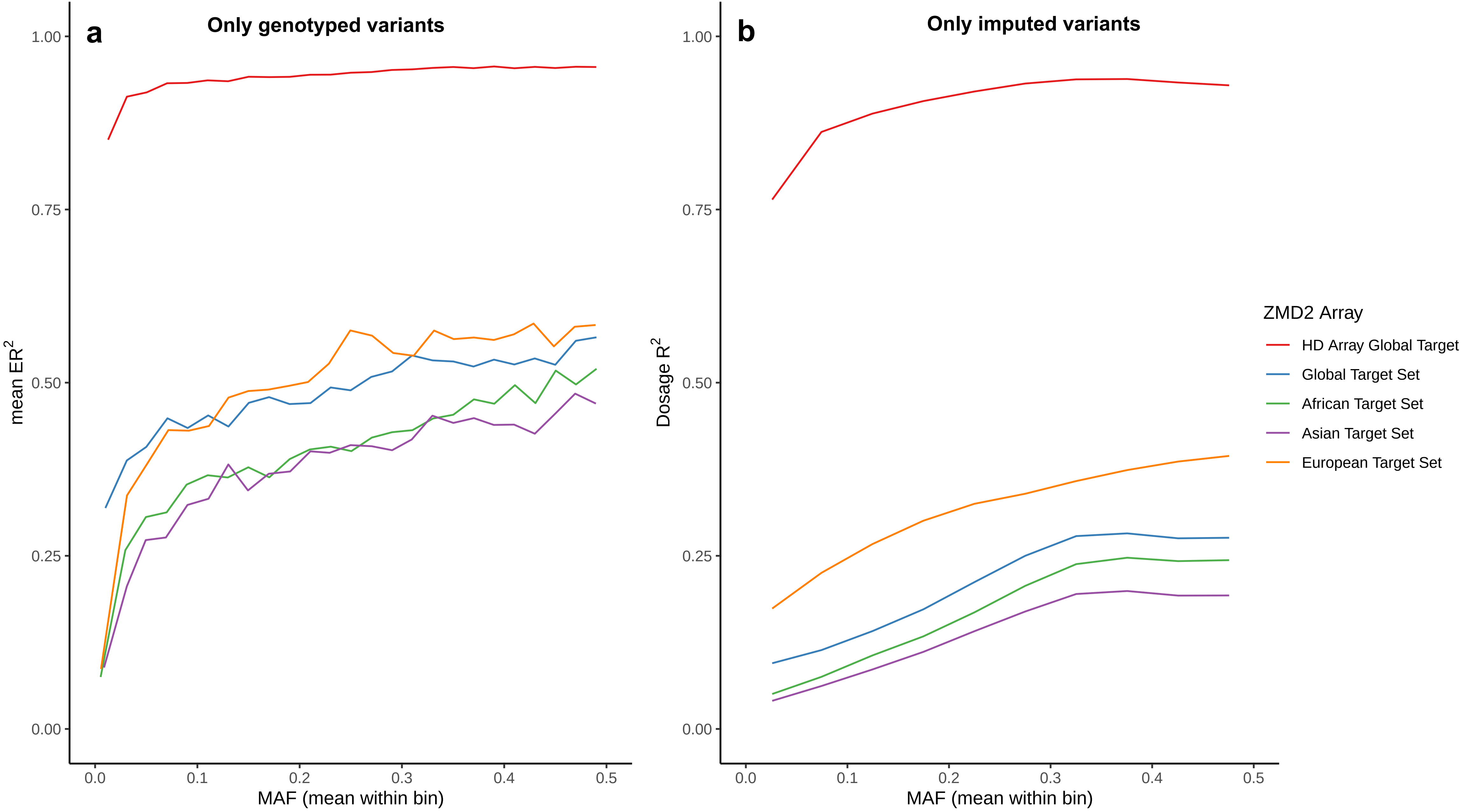

Additional file 9 Figure S12

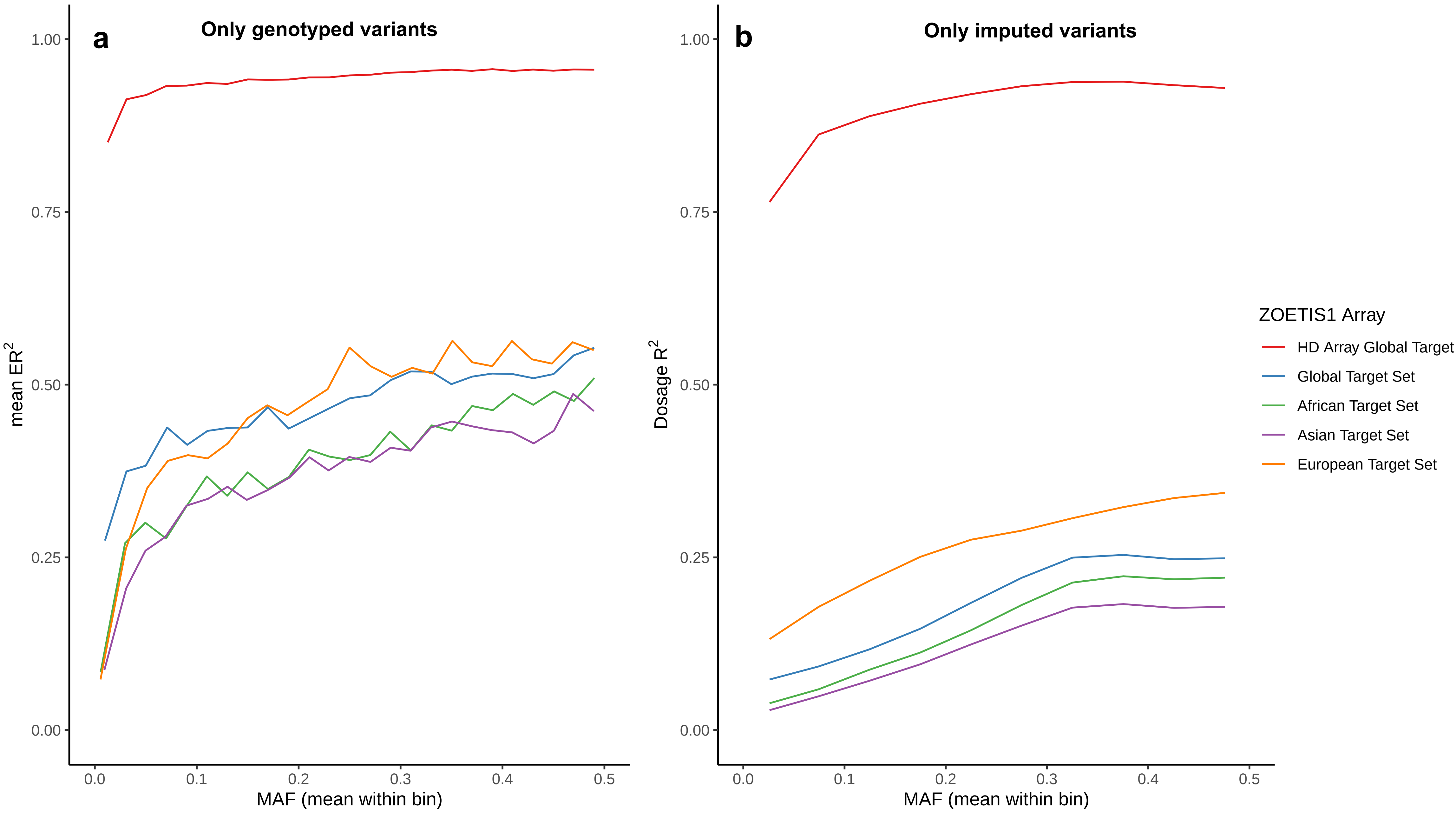

Additional file 9 Figure S13

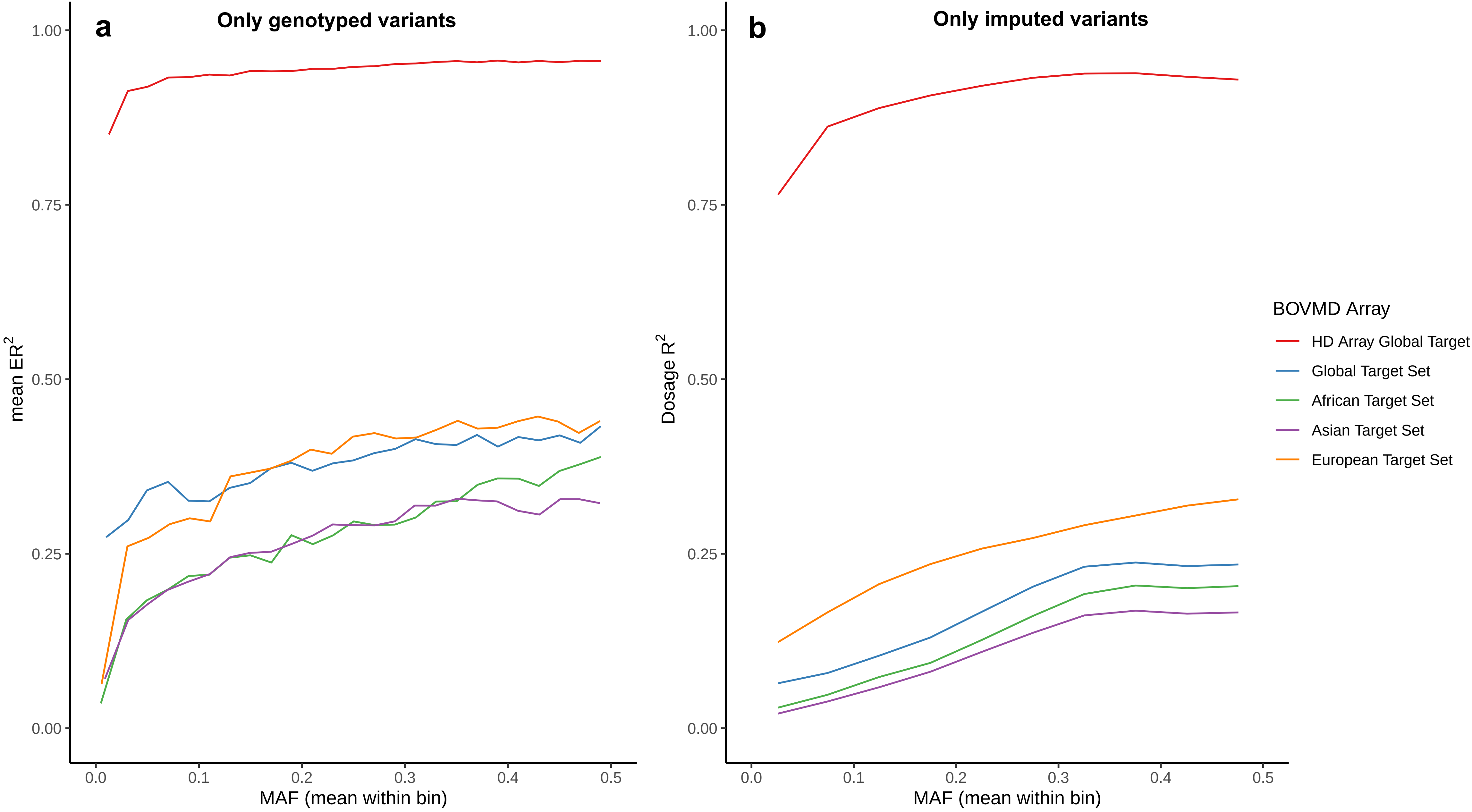

Additional file 9 Figure S14

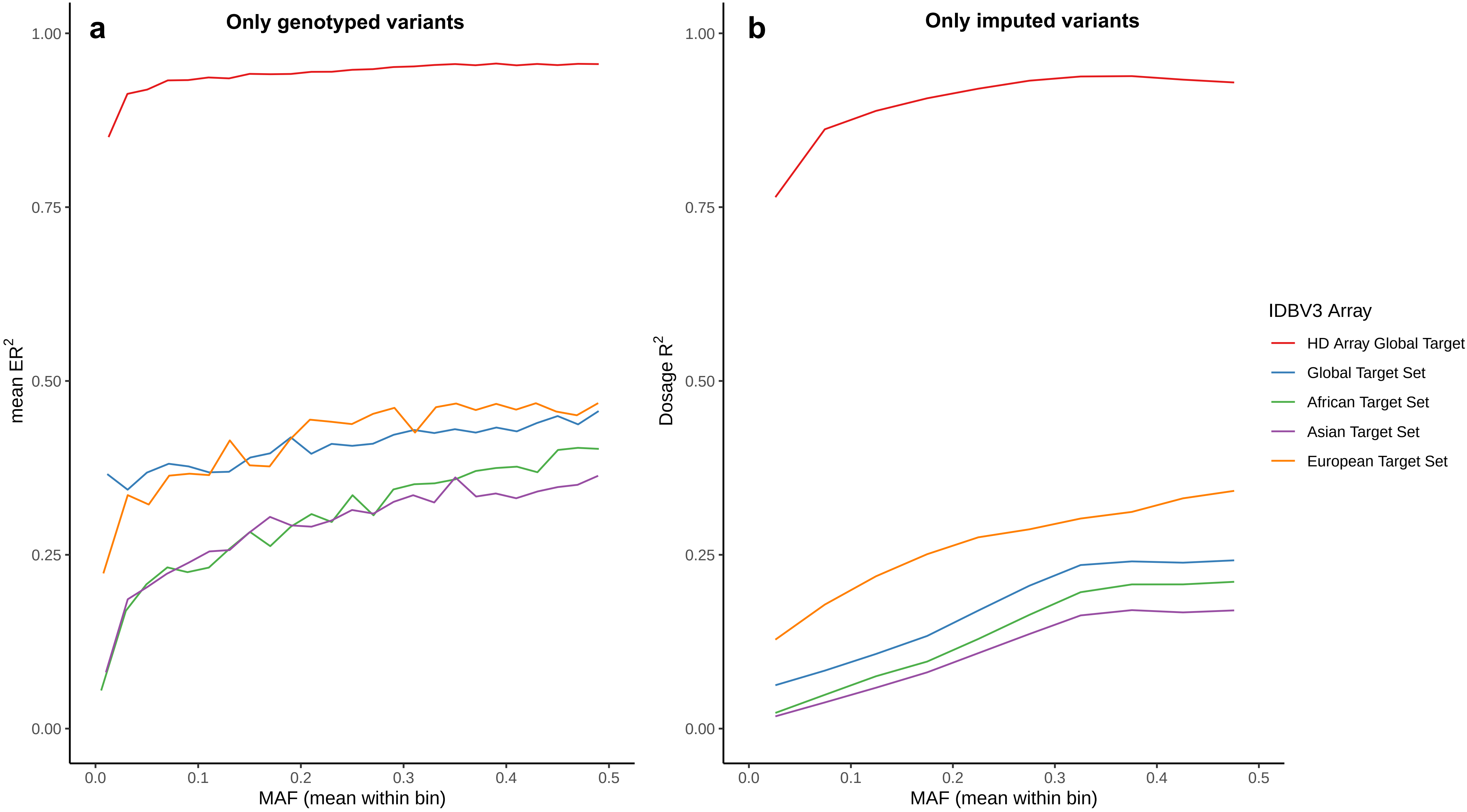

Additional file 9 Figure S15

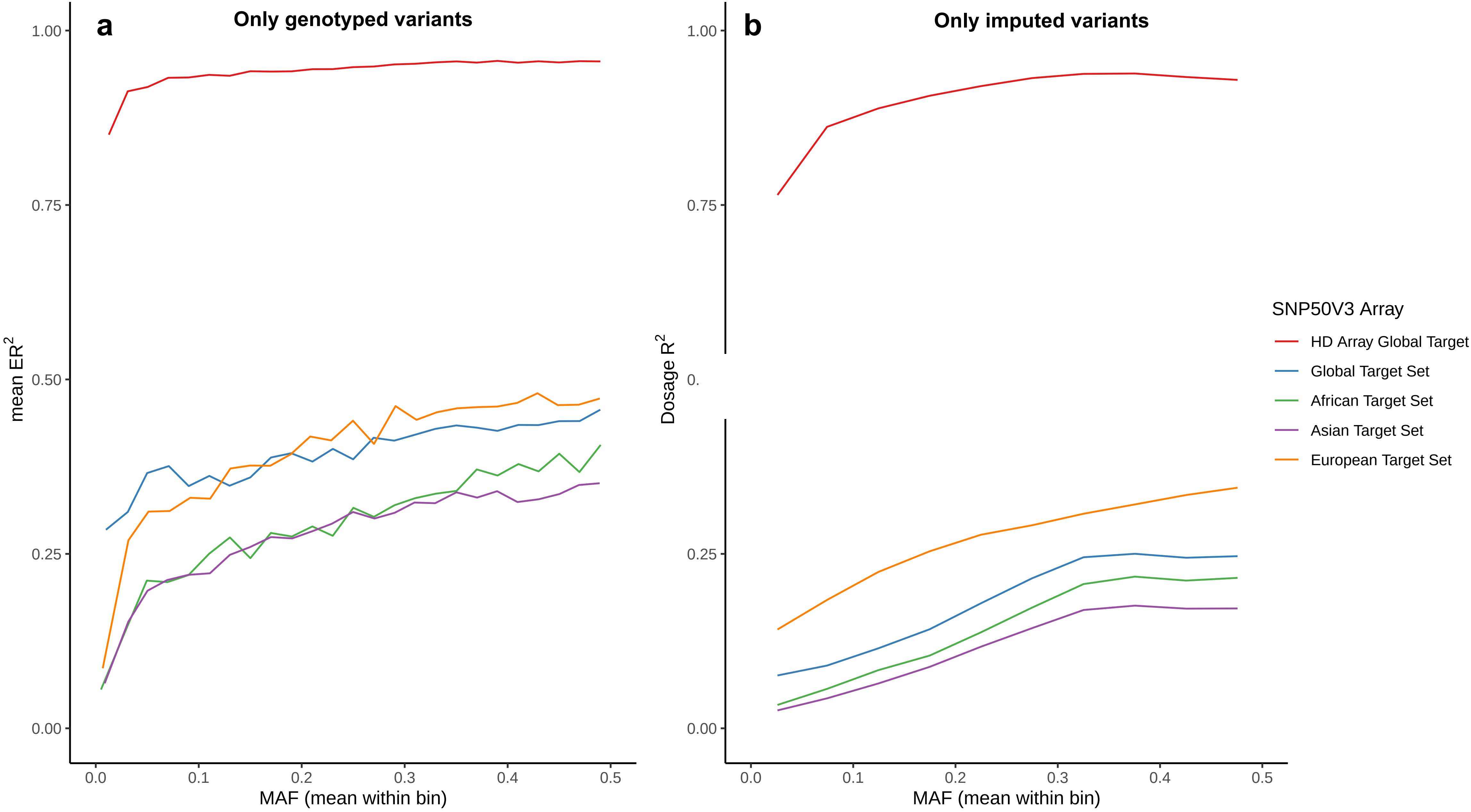

Additional file 9 Figure S16

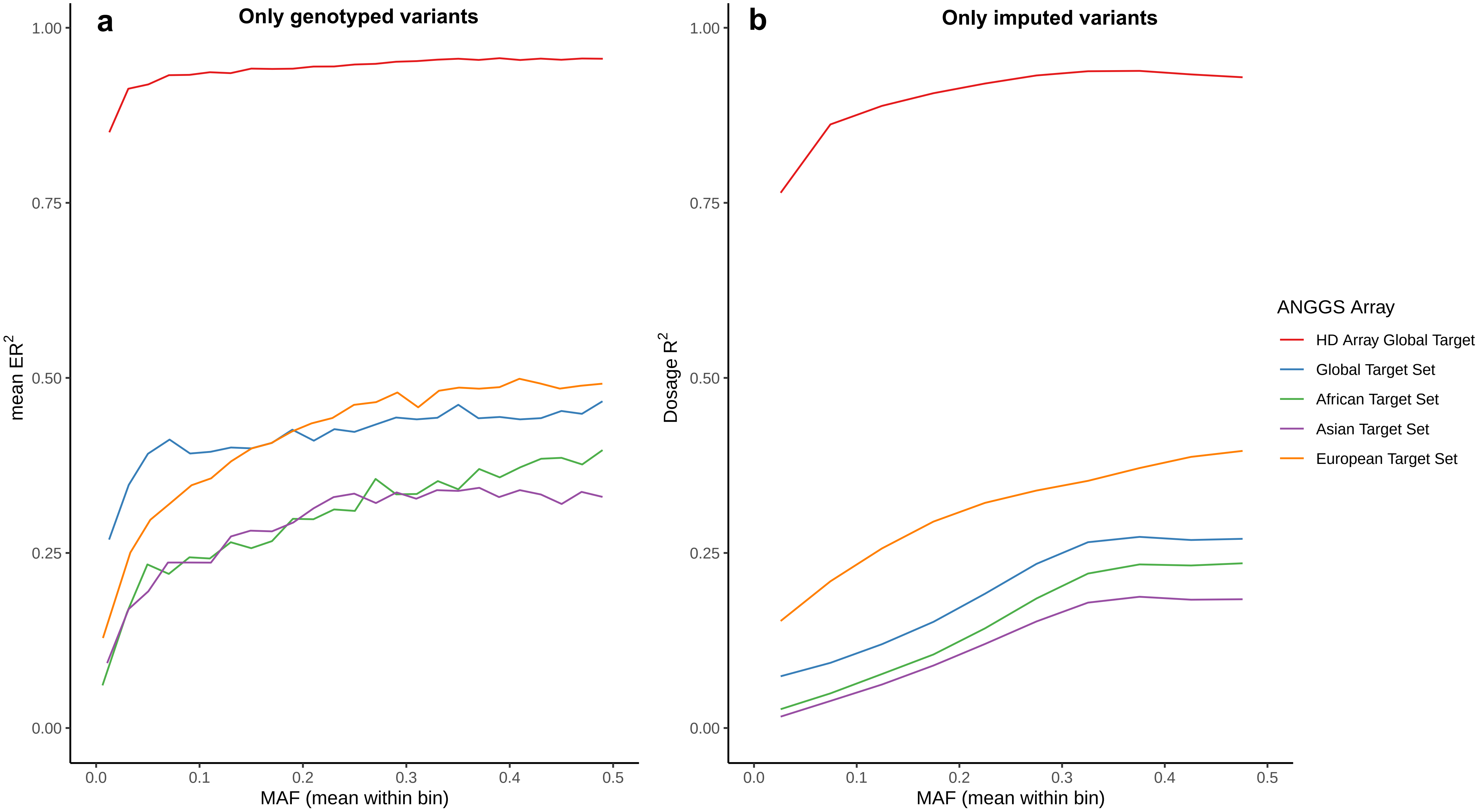

Additional file 9 Figure S17

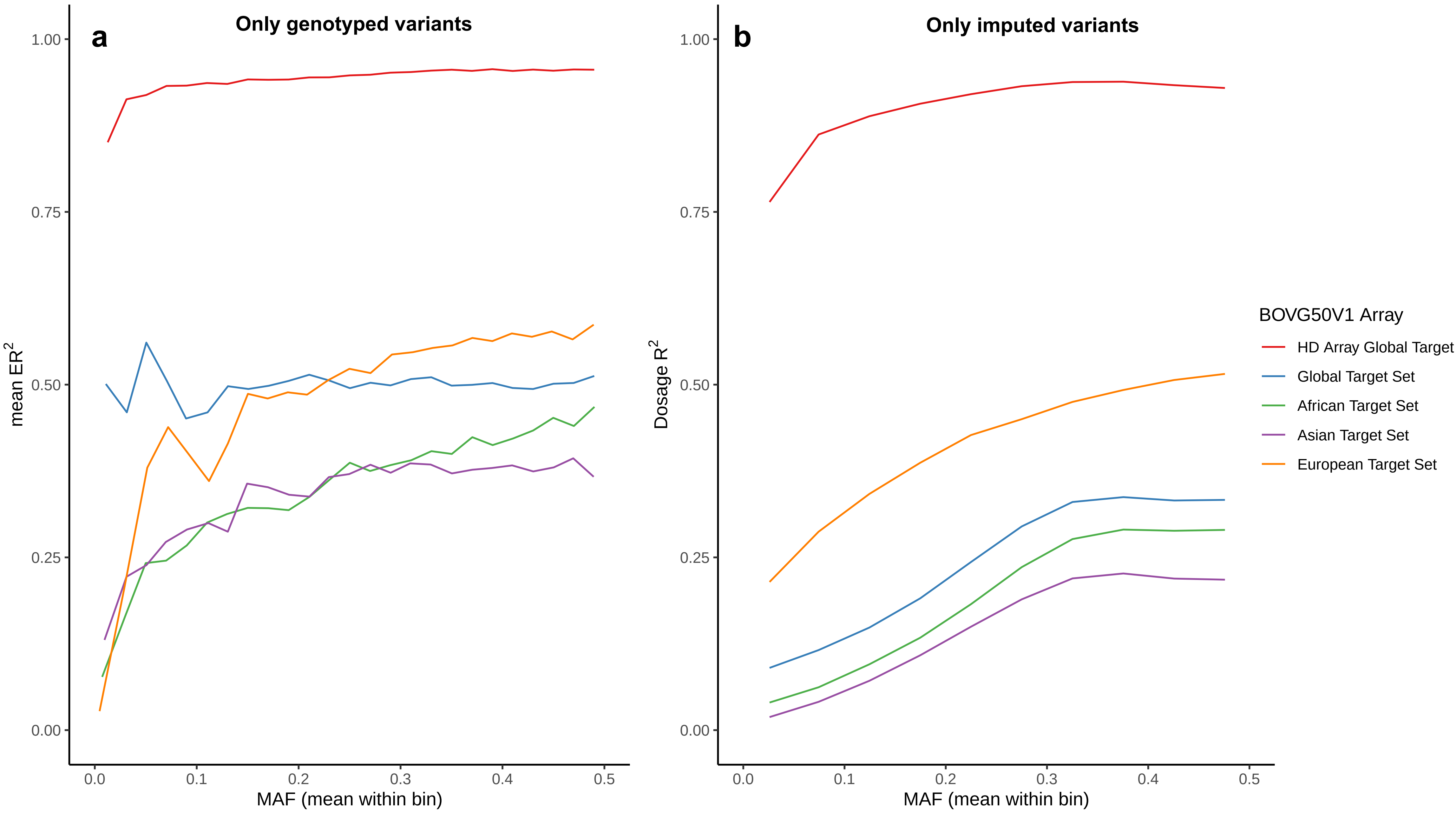

Additional file 9 Figure S18

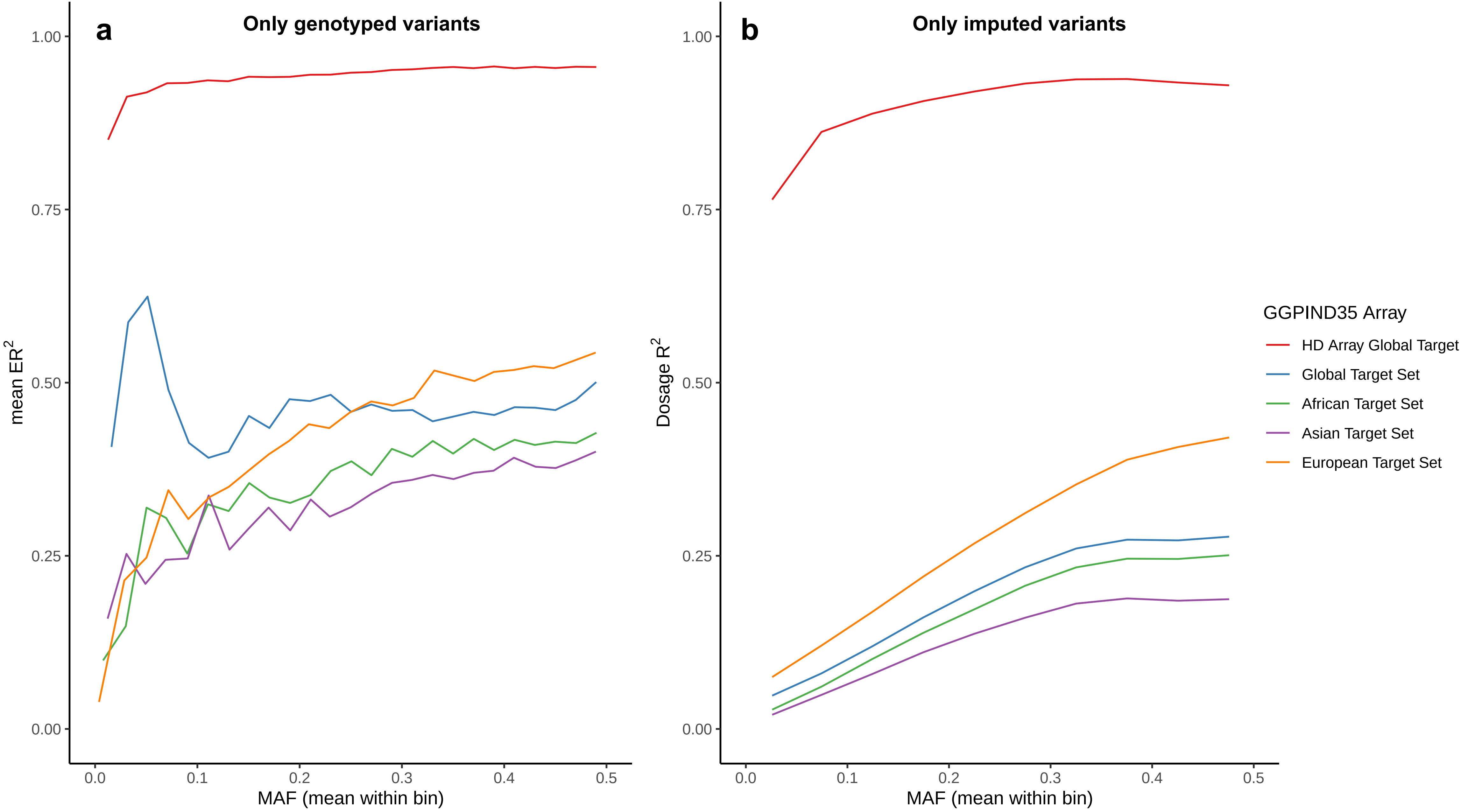

Additional file 9 Figure S19

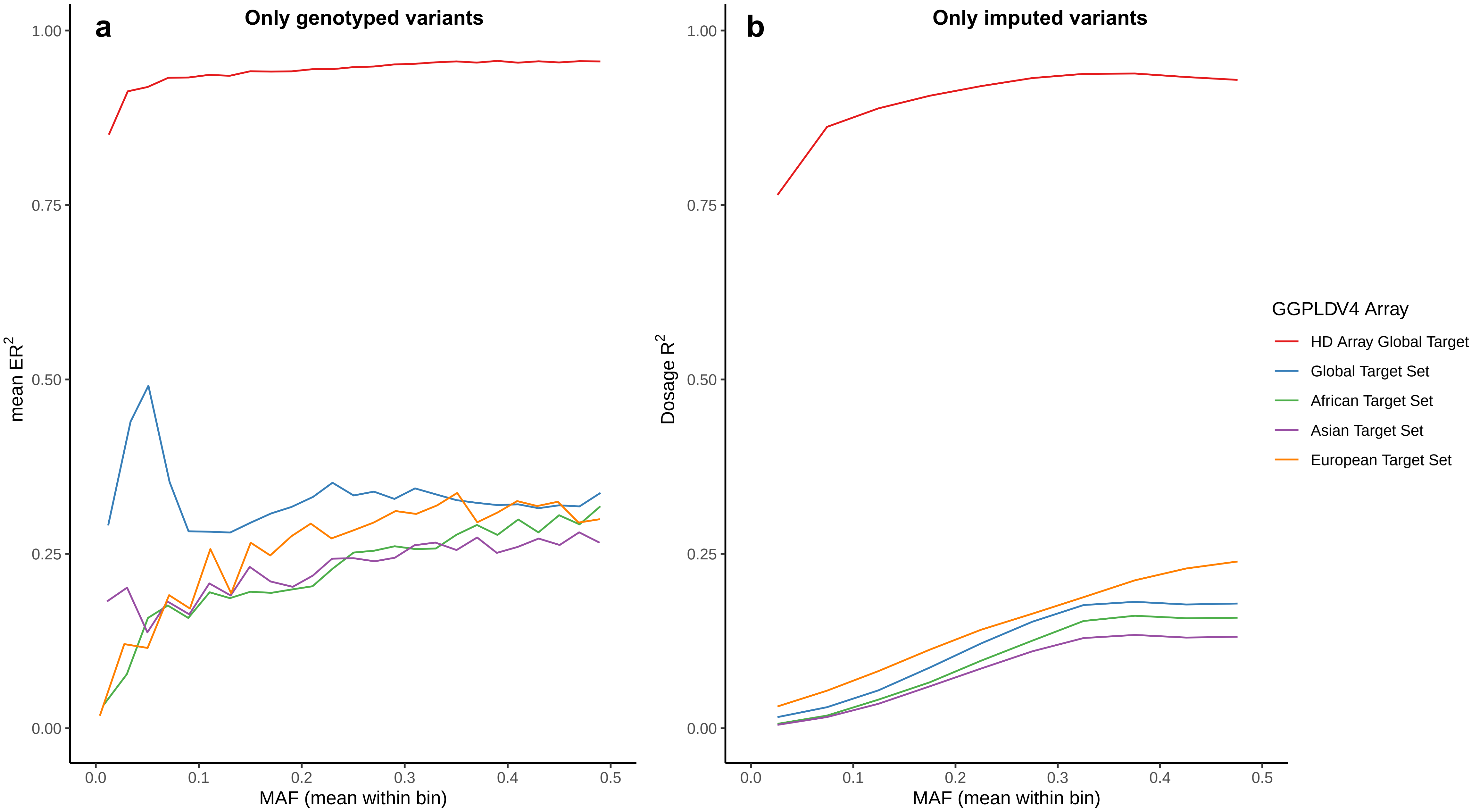

Additional file 9 Figure S20

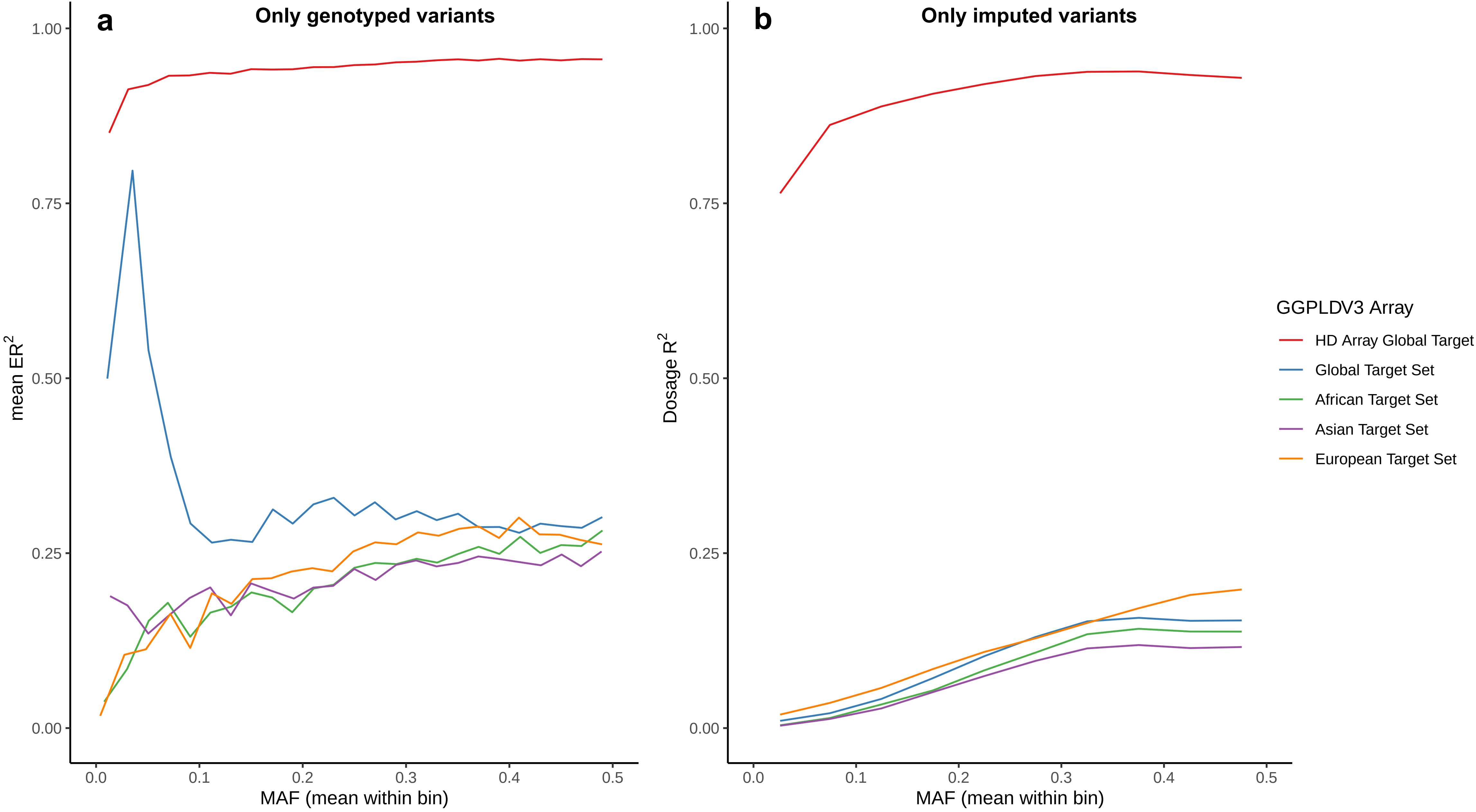

Supplement: Supplementary file 9 — Additional file 9: Figure S6. Imputation accuracies ER2 (A) and dosage R2 (B) for the BOS1 array when using the Global Reference Panel and four target sets (i.e., Global, African, Asian and European). The target sets were created by retaining only the WGS genotypes that overlapped with the variants of the BOS1 array, from the Global Reference Panel as well as its subsets, generated according to the continent of origin (African (87 individuals), Asian (106 individuals) and European (77 individuals) subsets). These target sets (i.e. Global, African, Asian, and European) were then used to impute to WGS level using the Global Reference Panel. The results for the HD array when using the Global target set are also reported. Figure S7. Imputation accuracies ER2 (A) and dosage R2 (B) for the GGPHDV3 array when using the Global Reference Panel and four target sets (i.e., Global, African, Asian and European). The target sets were created by retaining only the WGS genotypes that overlapped with the variants of the GGPHDV3 array, from the Global Reference Panel as well as its subsets, generated according to the continent of origin (African (87 individuals), Asian (106 individuals) and European (77 individuals) subsets). These target sets (i.e. Global, African, Asian, and European) were then used to impute to WGS level using the Global Reference Panel. The results for the HD array when using the Global target set are also reported. Figure S8. Imputation accuracies ER2 (A) and dosage R2 (B) for the GGPF250 array when using the Global Reference Panel and four target sets (i.e., Global, African, Asian and European). The target sets were created by retaining only the WGS genotypes that overlapped with the variants of the GGPF250 array, from the Global Reference Panel as well as its subsets, generated according to the continent of origin (African (87 individuals), Asian (106 individuals) and European (77 individuals) subsets). These target sets (i.e. Global, African, Asian, and Europ [file 12711_2022_751_MOESM9_ESM.pdf]
