# Supplementary material for: Assessment of genotyping array performance for genome-wide association studies and imputation in African cattle
Source: Genet Sel Evol. 2022 Sep 4;54:58. doi: 10.1186/s12711-022-00751-5 (PMC9441065; doi:10.1186/s12711-022-00751-5)

Additional file 10 Figure S21

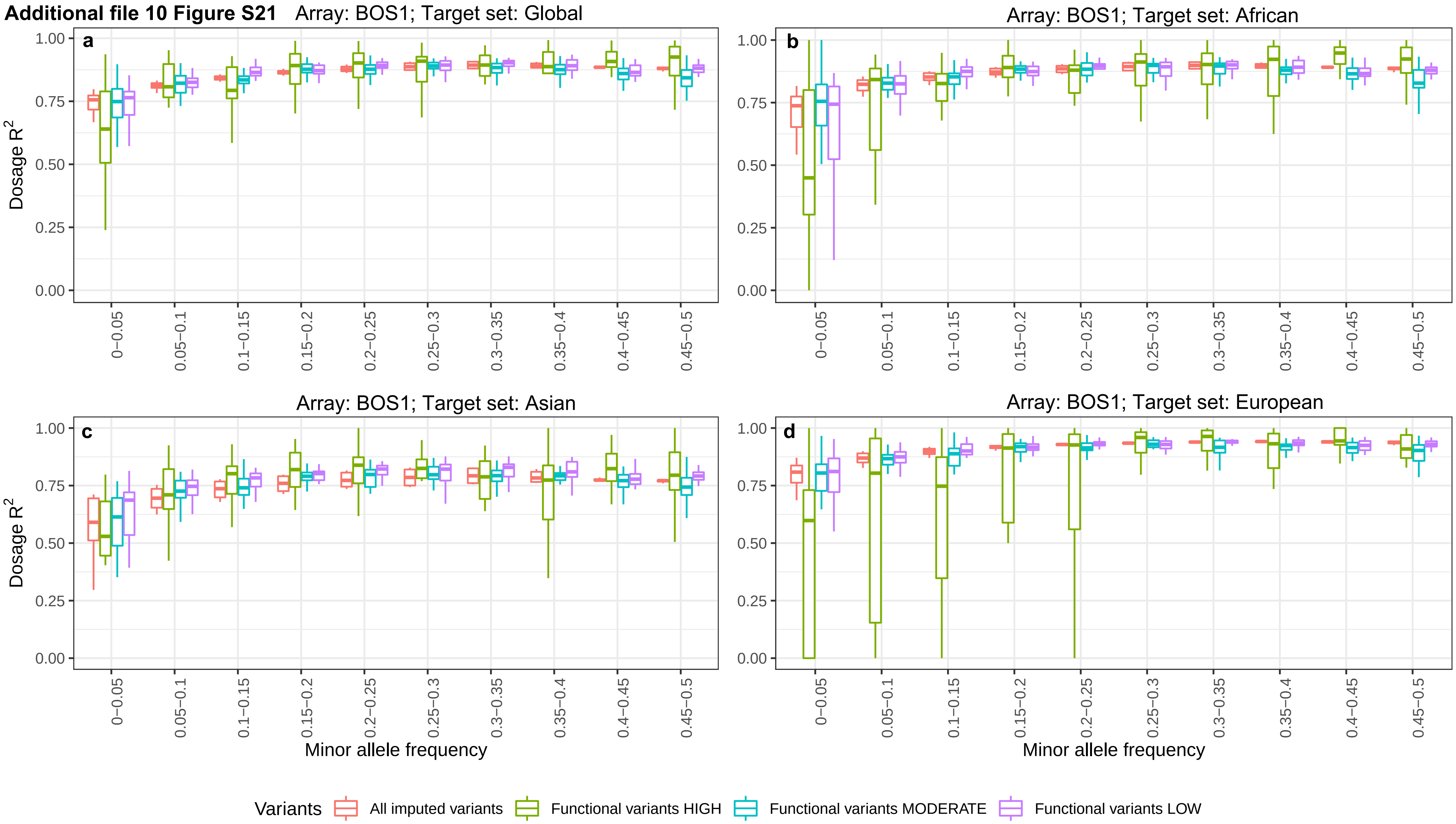

Additional file 10 Figure S22

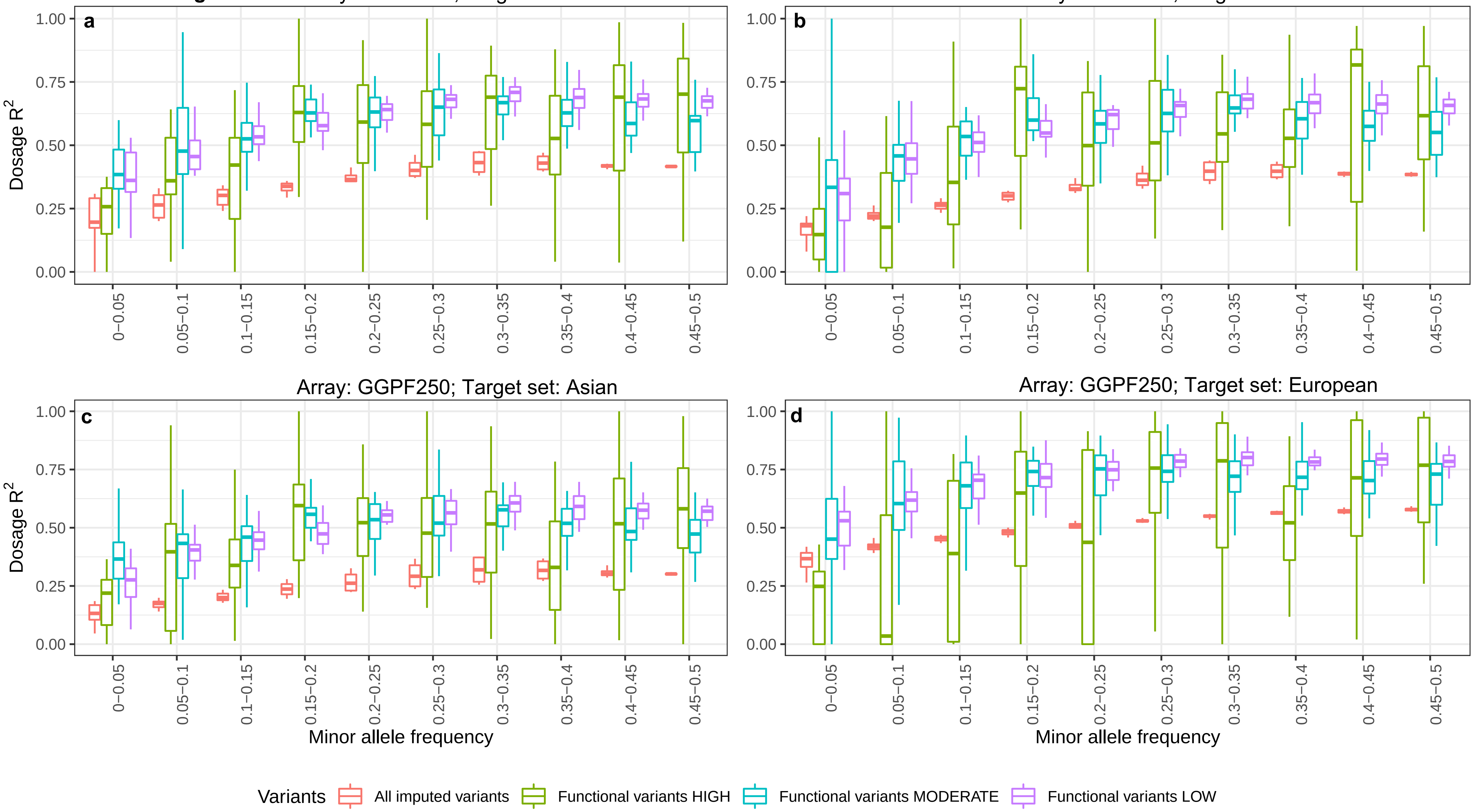

Additional file 10 Figure S23

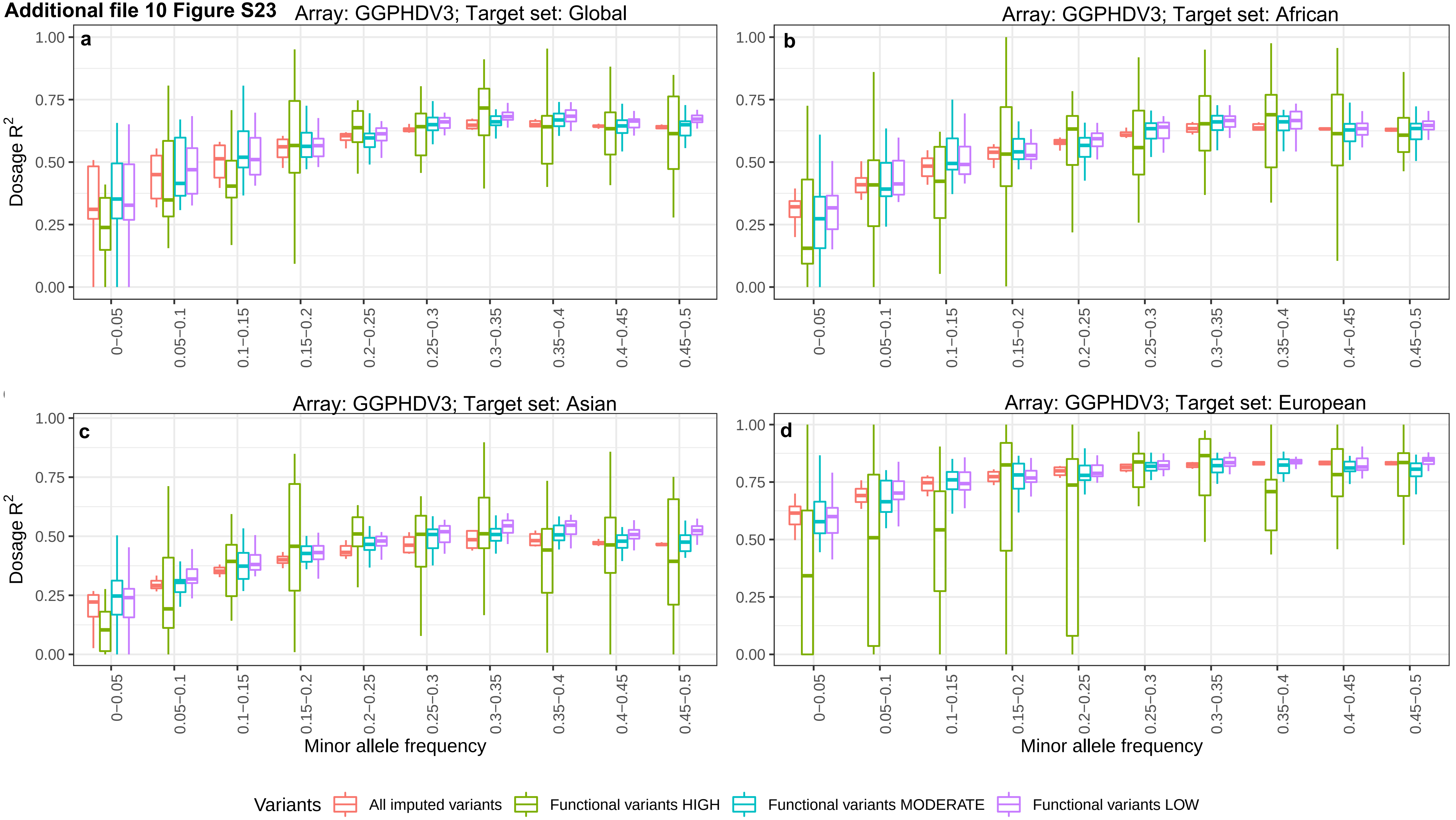

Additional file 10 Figure S24

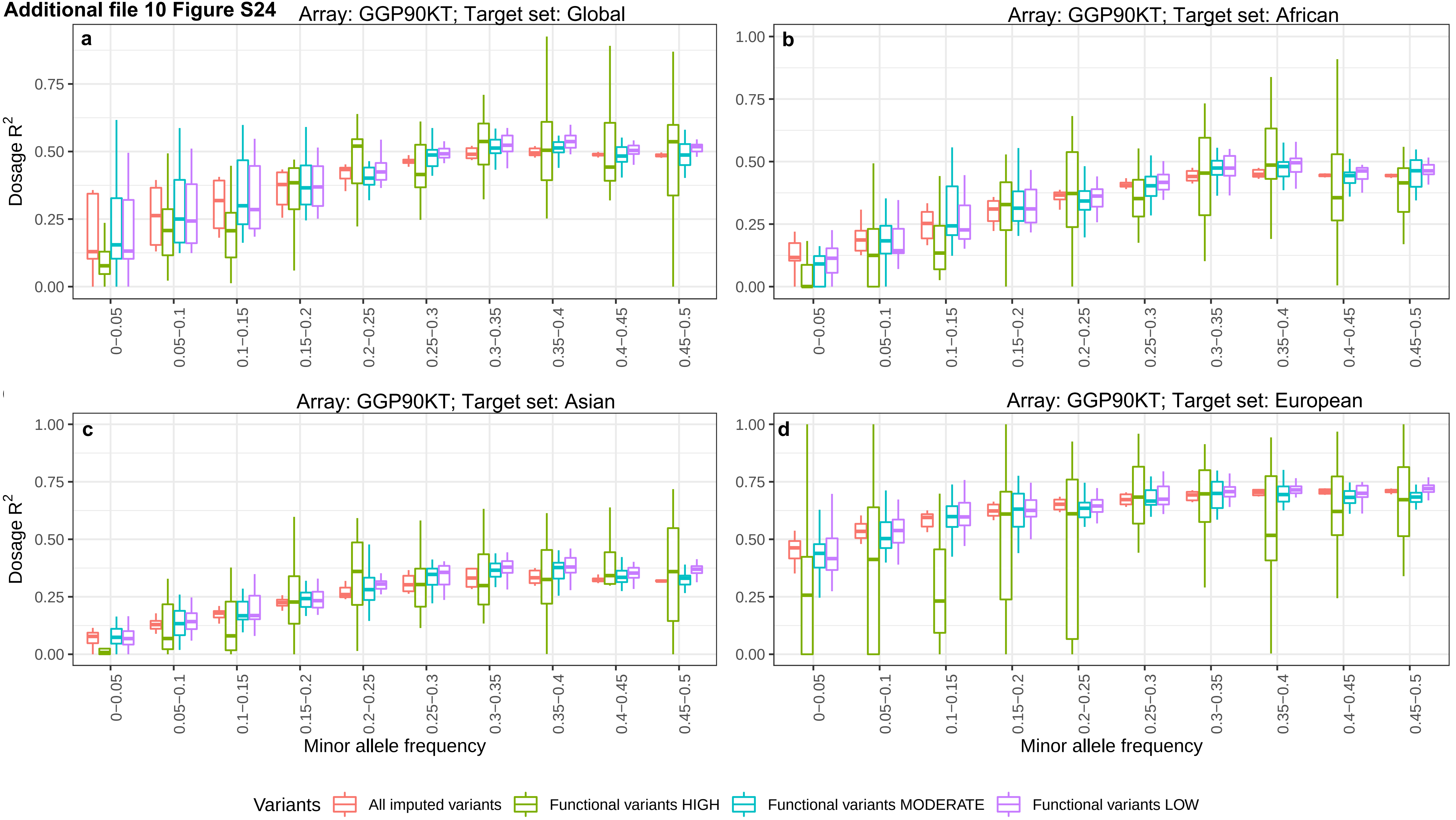

Additional file 10 Figure S25

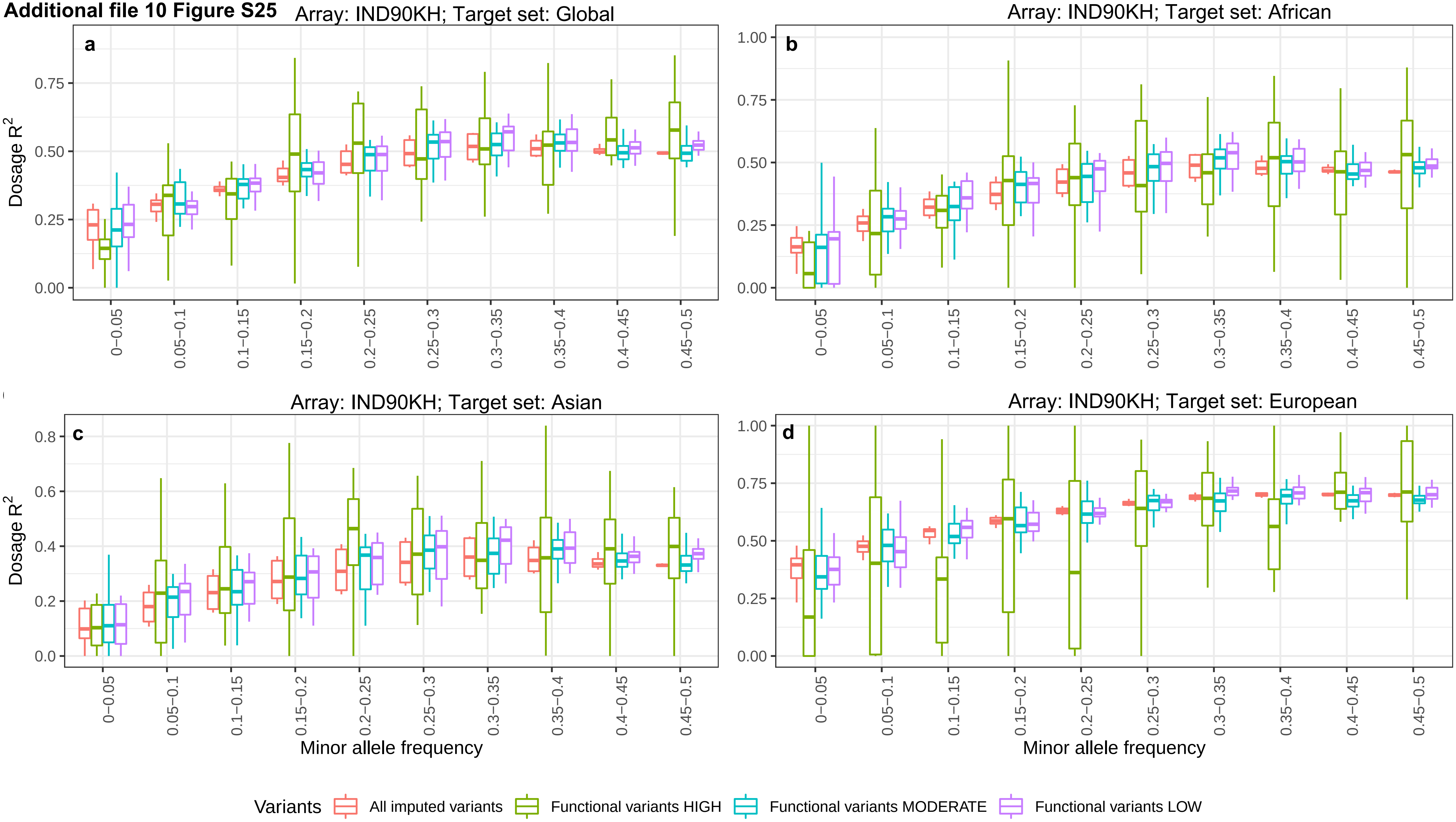

Additional file 10 Figure S26

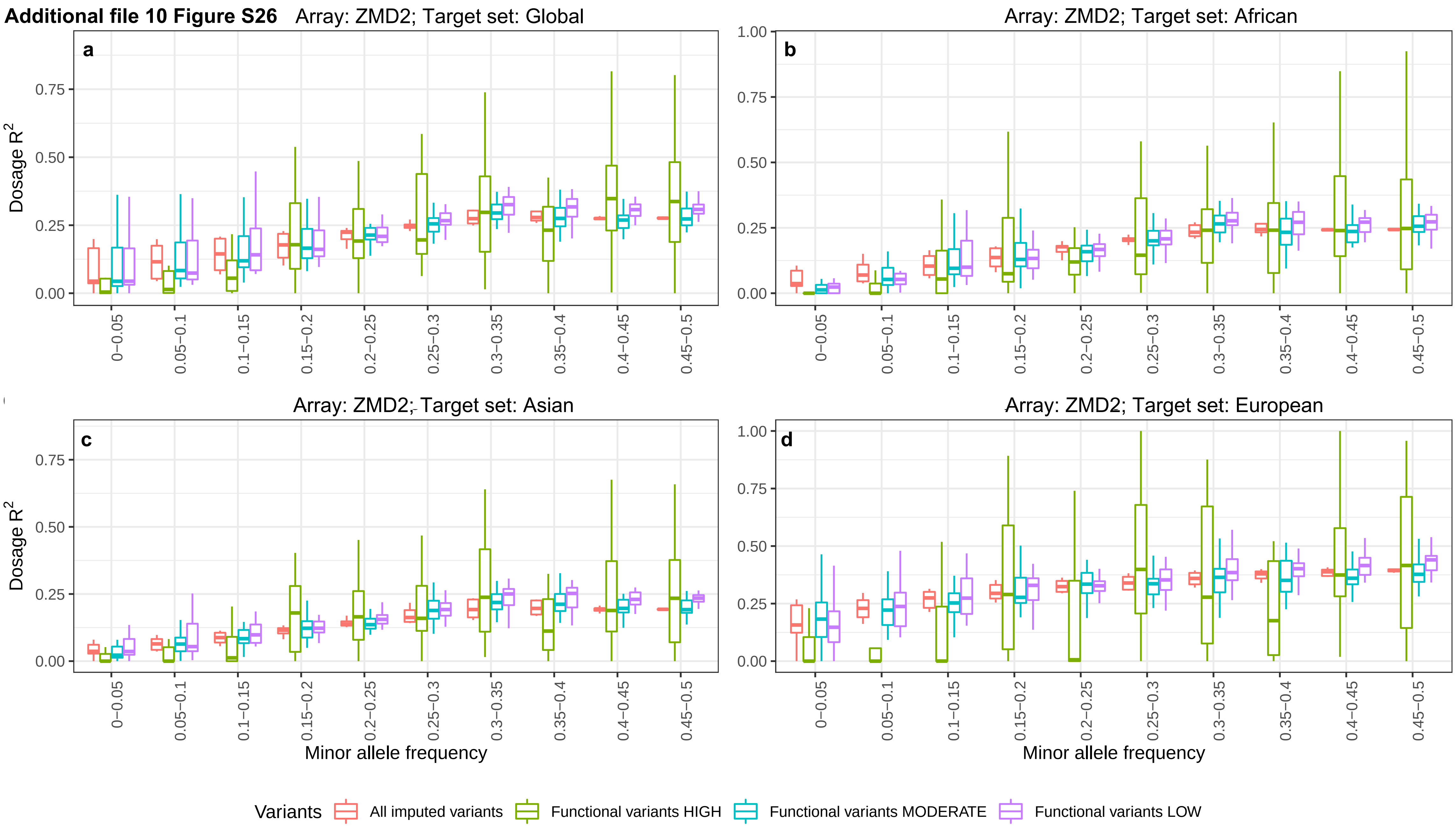

Additional file 10 Figure S27

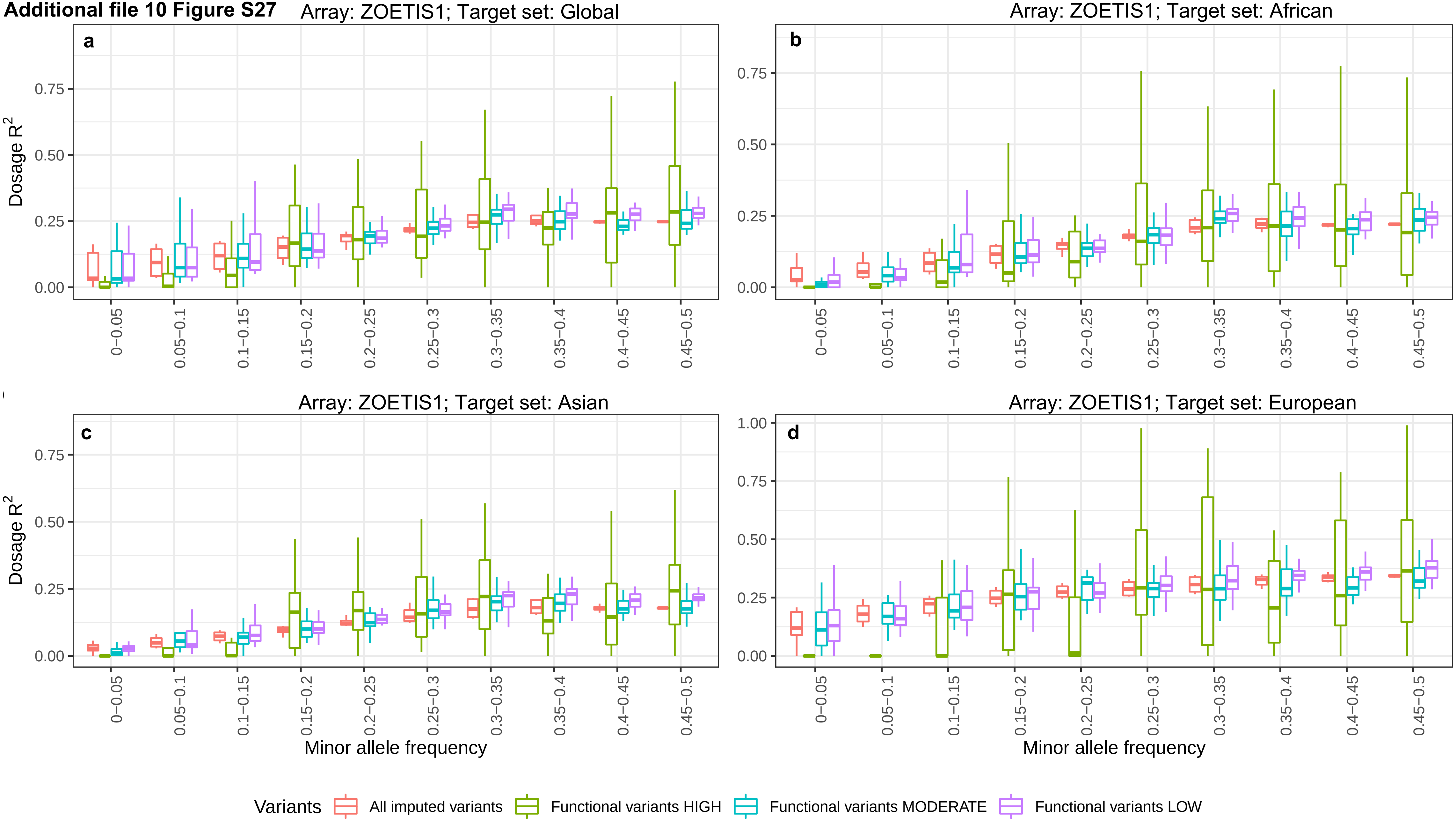

Additional file 10 Figure S28

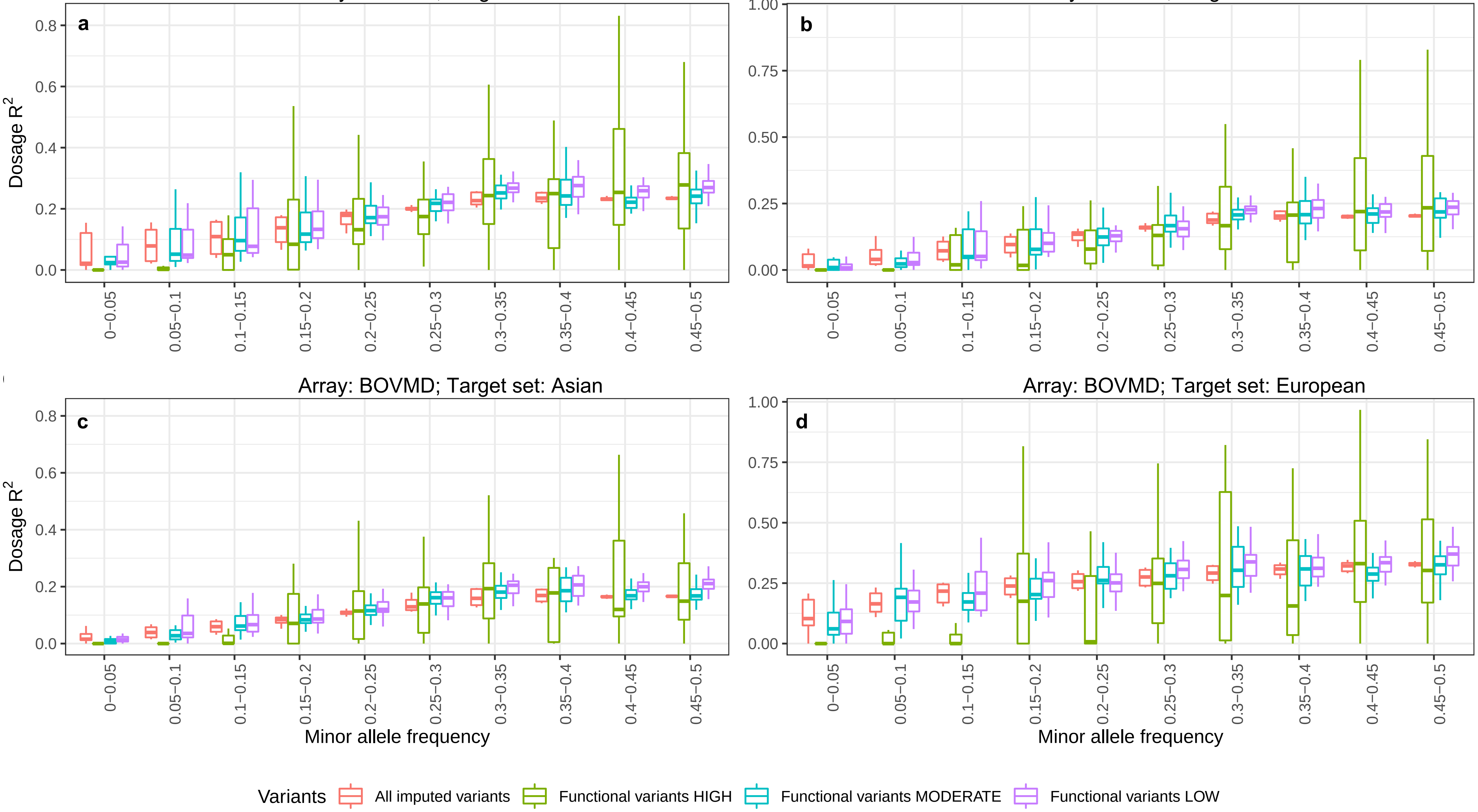

Additional file 10 Figure S29

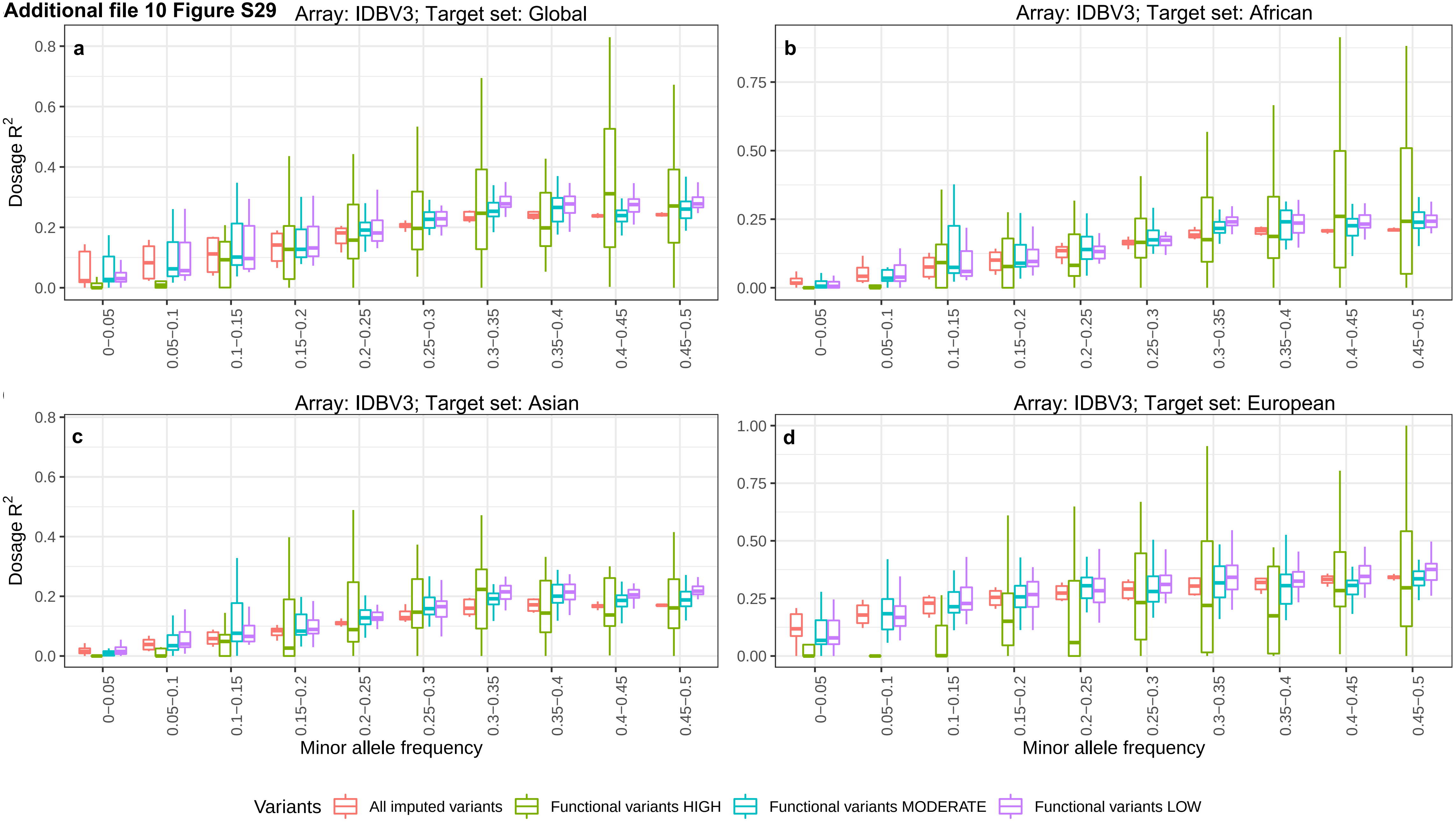

Additional file 10 Figure S30

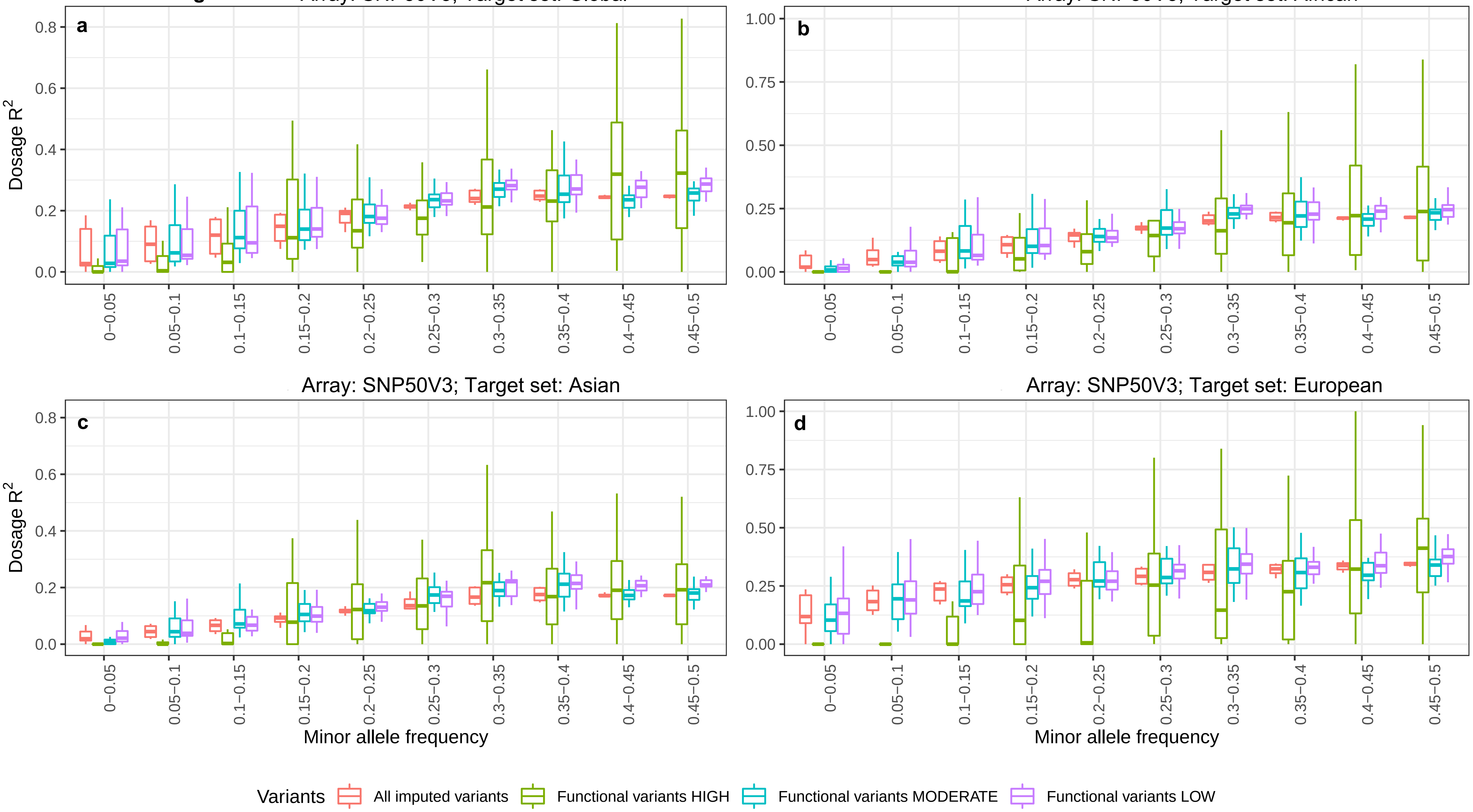

Additional file 10 Figure S31

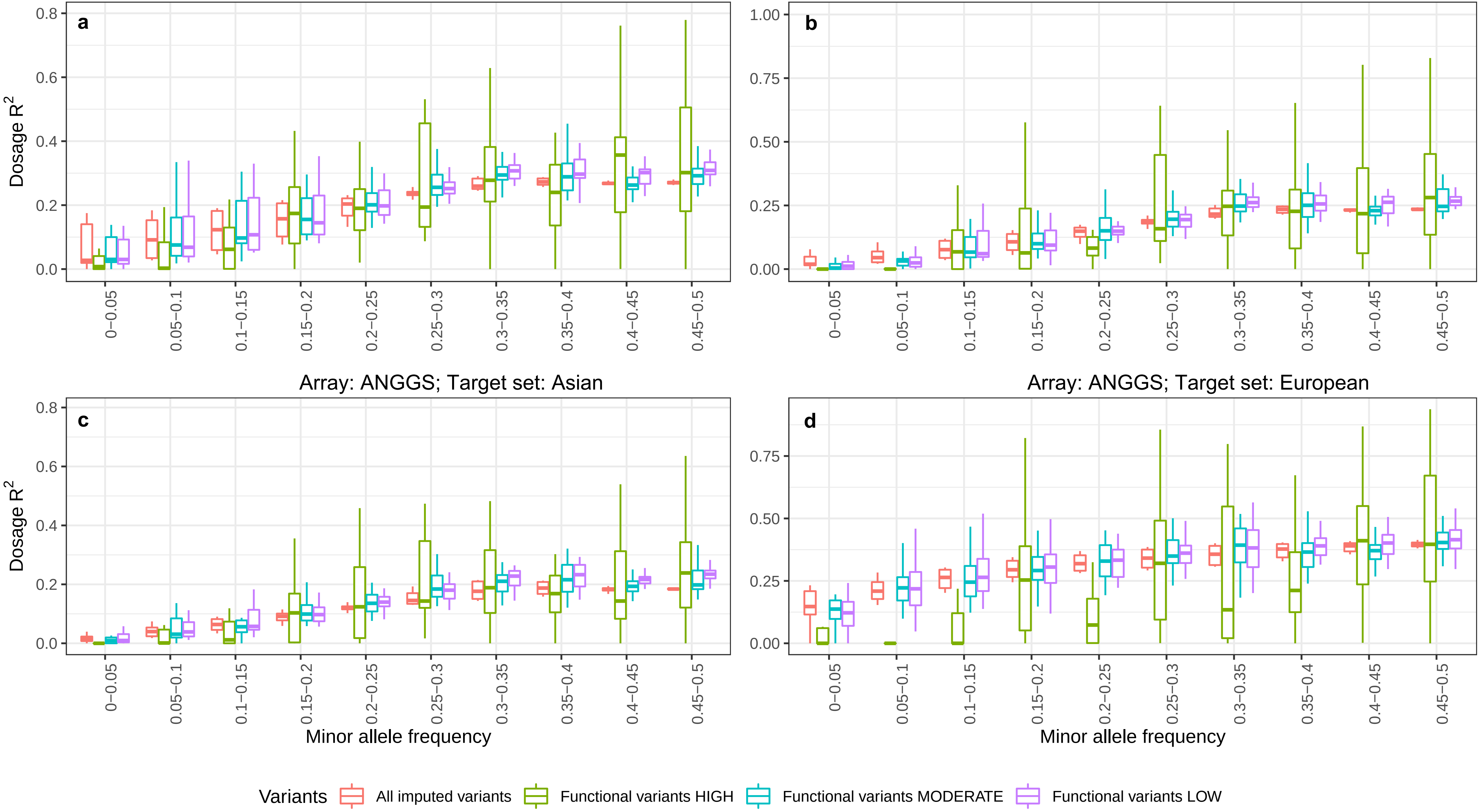

Additional file 10 Figure S32

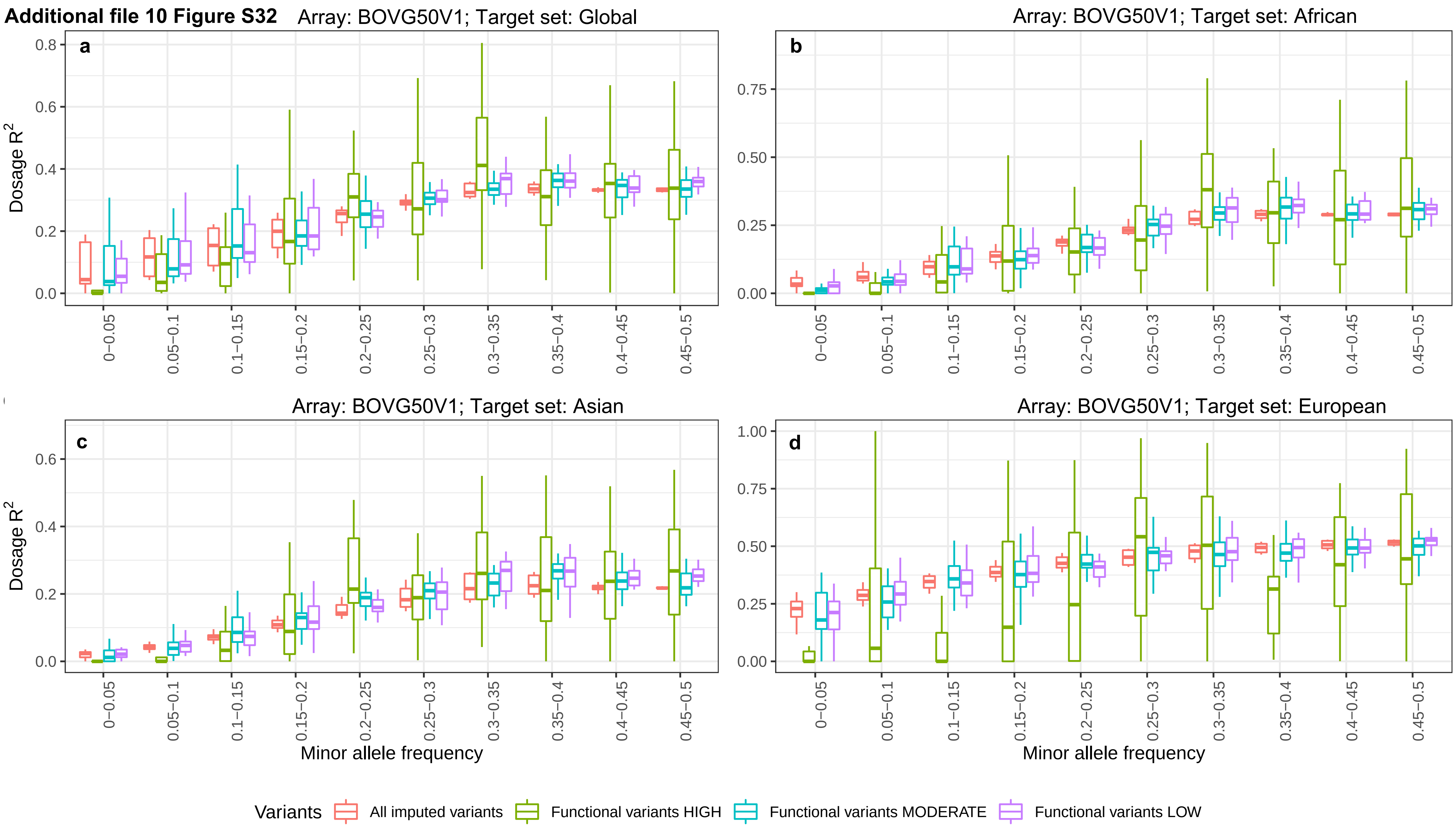

Additional file 10 Figure S33

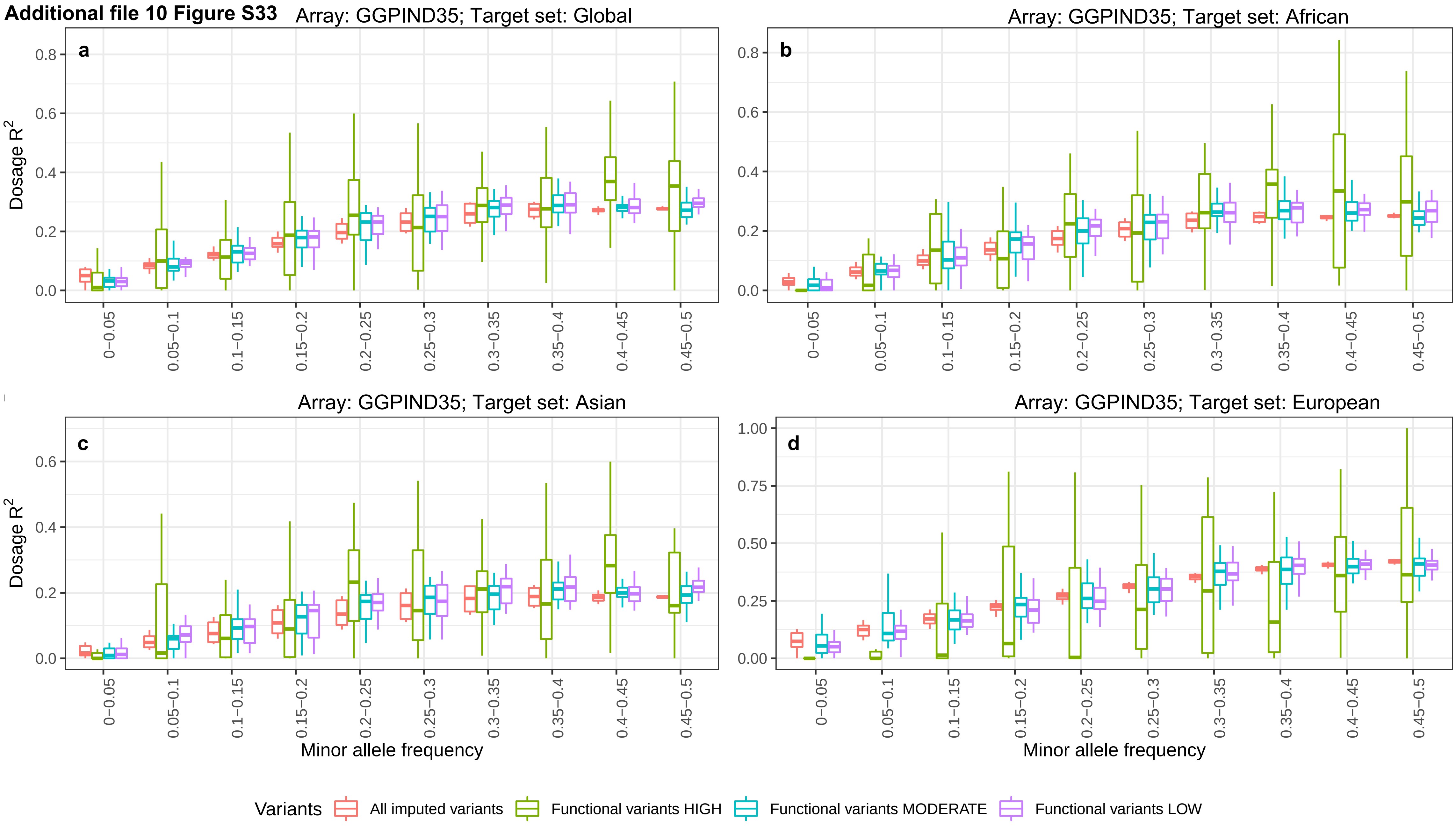

Additional file 10 Figure S34

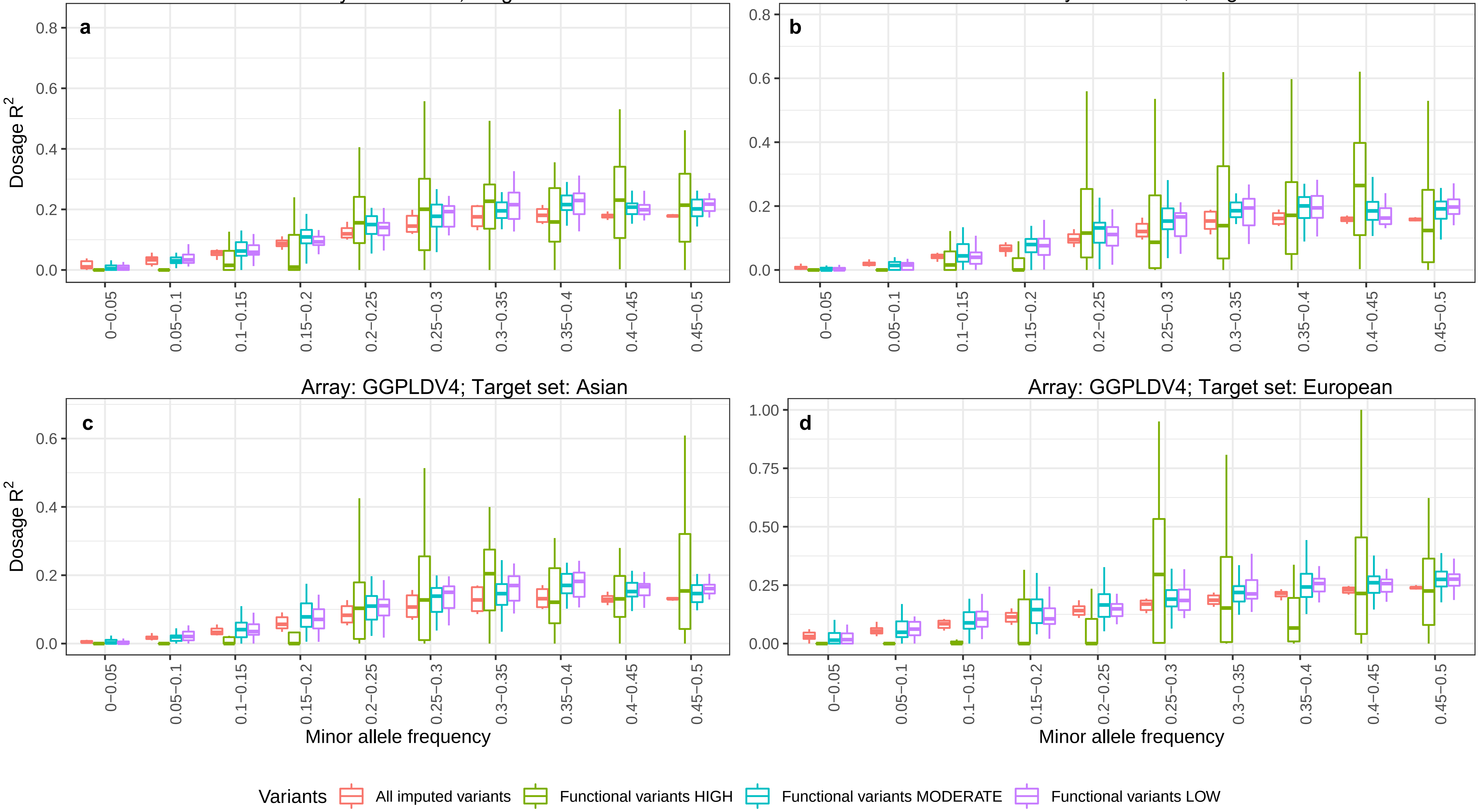

Additional file 10 Figure S35

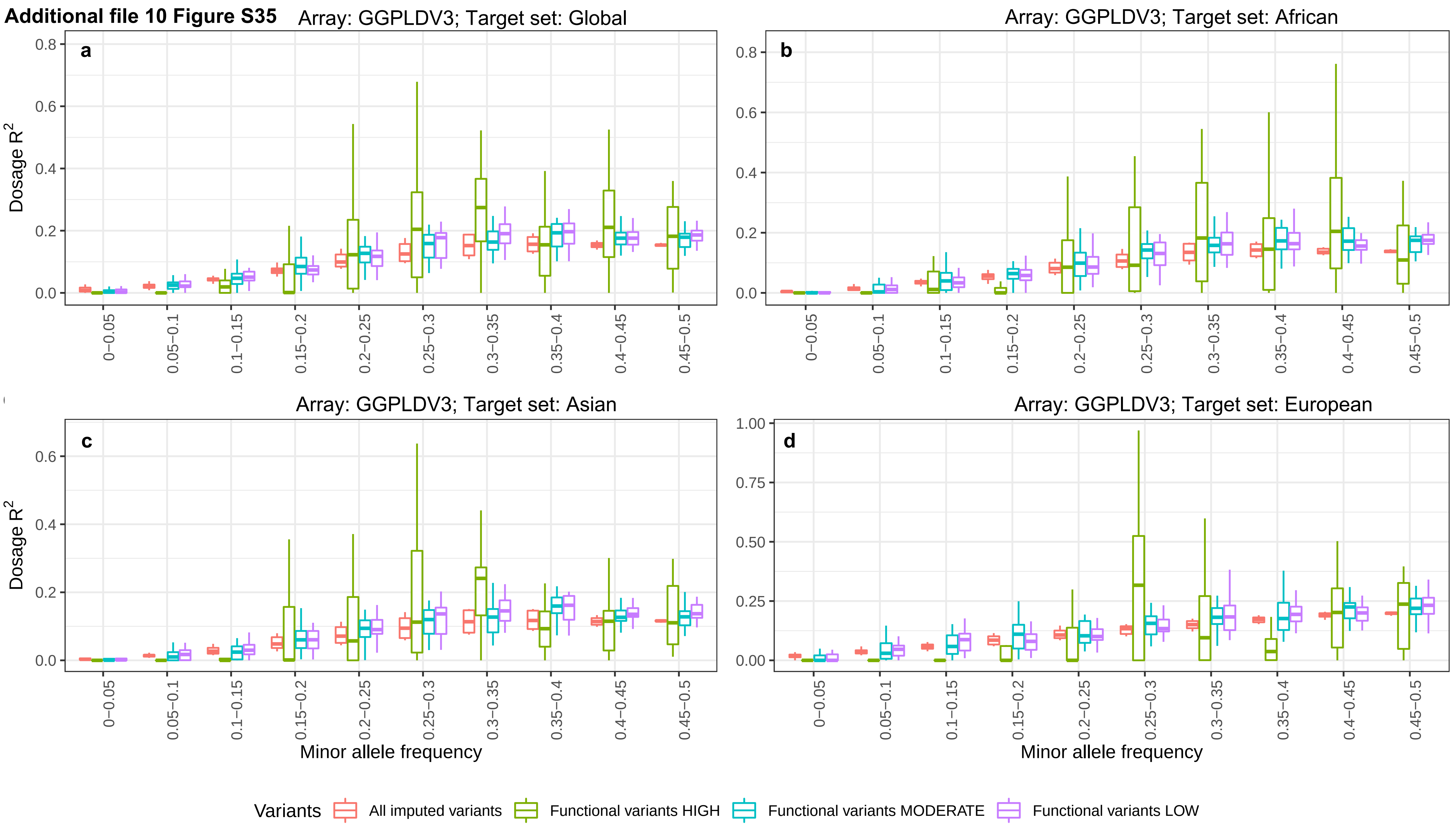

Supplement: Supplementary file 10 — Additional file 10: Figure S21. Dosage R2 for all imputed variants and for functional variants for the BOS1 array when using the Global Reference Panel and four target sets (i.e., Global, African, Asian and European). Dosage R2 for all imputed variants and for functional variants (as annotated by the Ensembl VEP software (LOW, MODERATE and HIGH)) when imputing from the BOS1 array to WGS level. The target sets were created by retaining only the WGS genotypes that overlapped with the variants of the BOS1 array, from the Global Reference Panel as well as its subsets, generated according to the continent of origin (African (87 individuals), Asian (106 individuals) and European (77 individuals) subsets). These target sets (i.e. Global, African, Asian, and European) were then used to impute to WGS level using the Global Reference Panel. Figure S22. Dosage R2 for all imputed variants and for functional variants for the GGPF250 array when using the Global Reference Panel and four target sets (i.e., Global, African, Asian and European). Dosage R2 for all imputed variants and for functional variants (as annotated by the Ensembl VEP software (LOW, MODERATE and HIGH)) when imputing from the GGPF250 array to WGS level. The target sets were created by retaining only the WGS genotypes that overlapped with the variants of the GGPF250 array, from the Global Reference Panel as well as its subsets, generated according to the continent of origin (African (87 individuals), Asian (106 individuals) and European (77 individuals) subsets). These target sets (i.e. Global, African, Asian, and European) were then used to impute to WGS level using the Global Reference Panel. Figure S23. Dosage R2 for all imputed variants and for functional variants for the GGPHDV3 array when using the Global Reference Panel and four target sets (i.e., Global, African, Asian and European). Dosage R2 for all imputed variants and for functional variants (as annotated by the Ensembl VEP software (LOW, MODERATE and [file 12711_2022_751_MOESM10_ESM.pdf]
