# Supplementary material for: Assessment of genotyping array performance for genome-wide association studies and imputation in African cattle
Source: Genet Sel Evol. 2022 Sep 4;54:58. doi: 10.1186/s12711-022-00751-5 (PMC9441065; doi:10.1186/s12711-022-00751-5)

Additional file 11 Figure S36

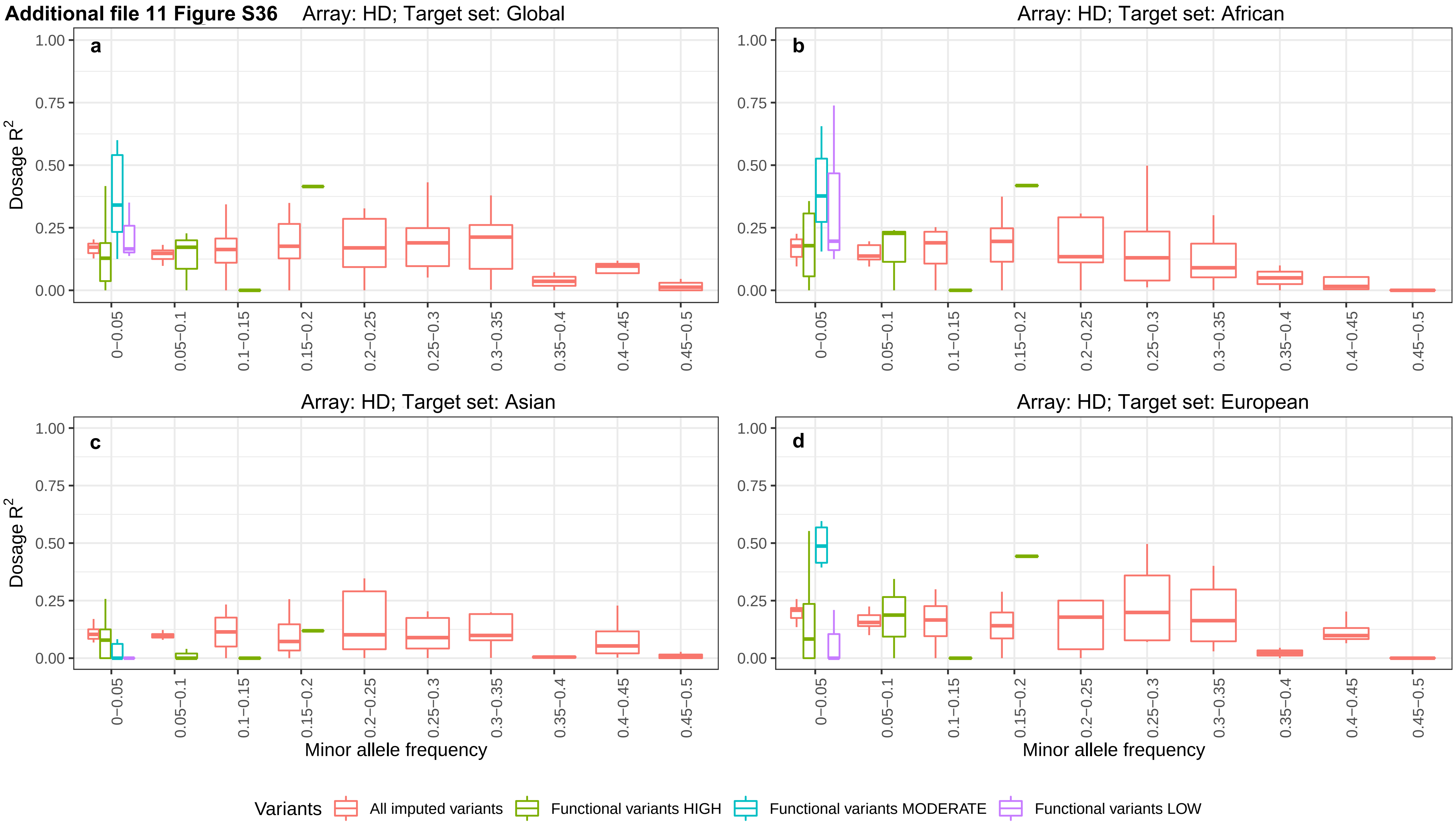

Additional file 11 Figure S37

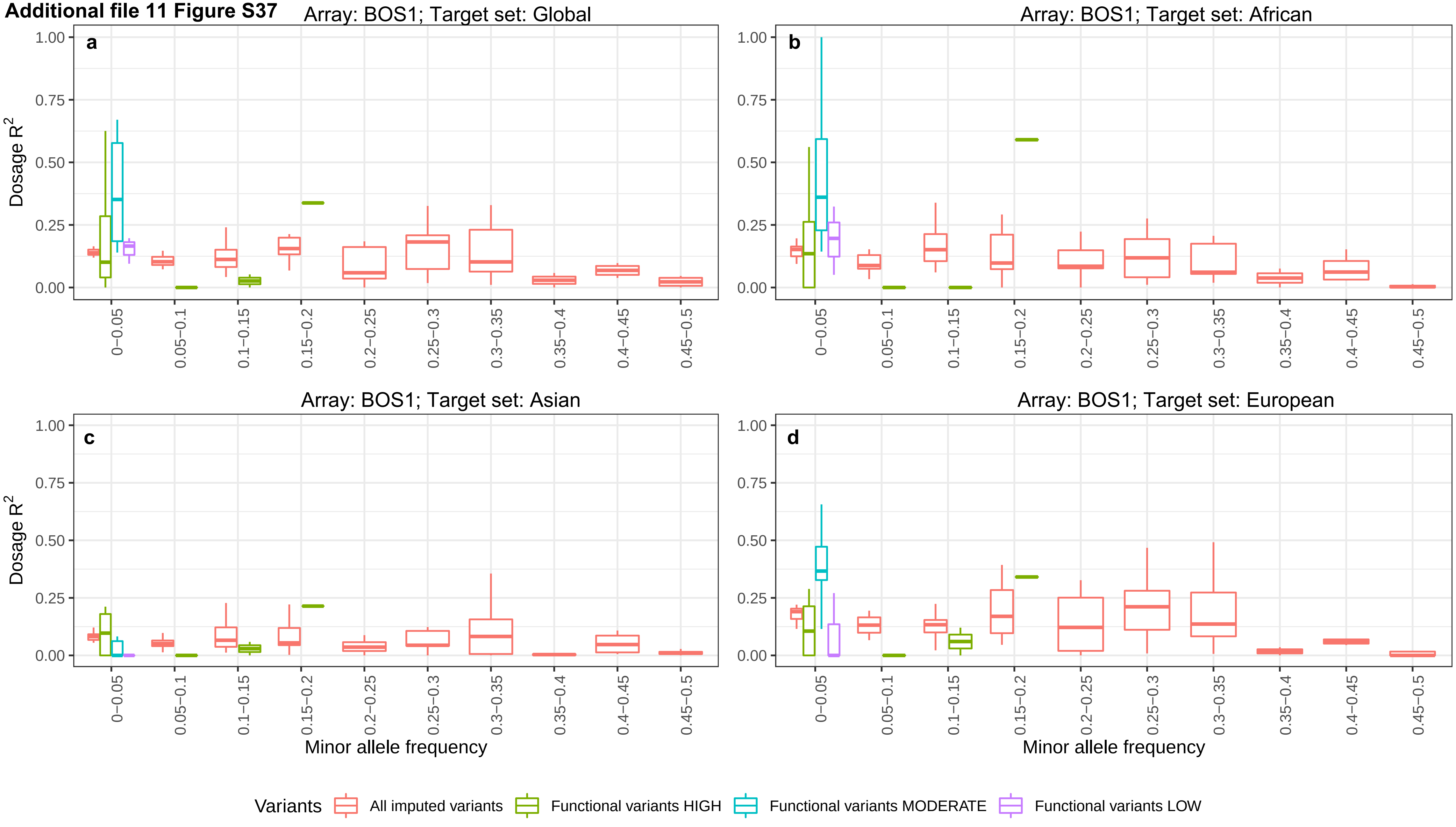

Additional file 11 Figure S38

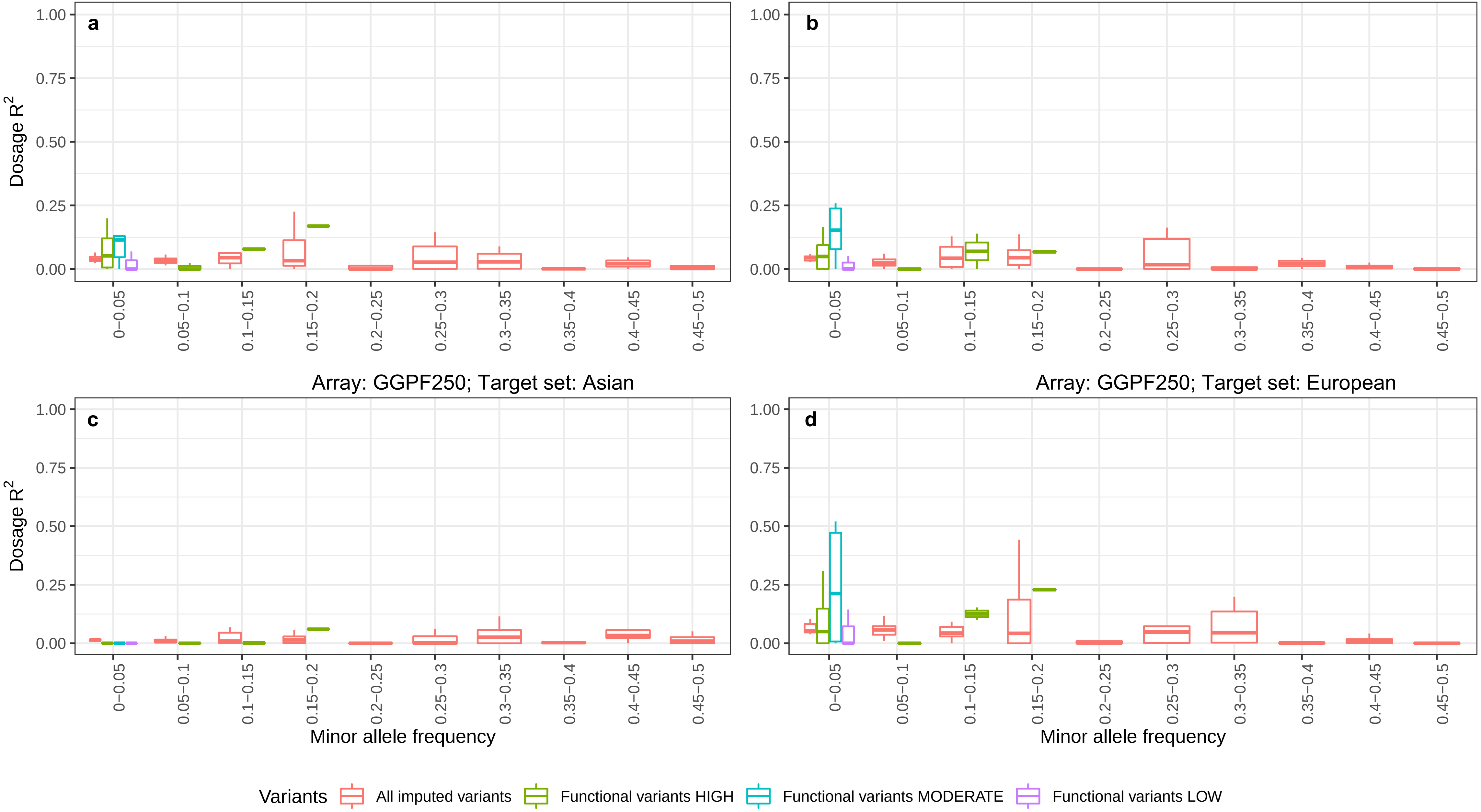

Additional file 11 Figure S39

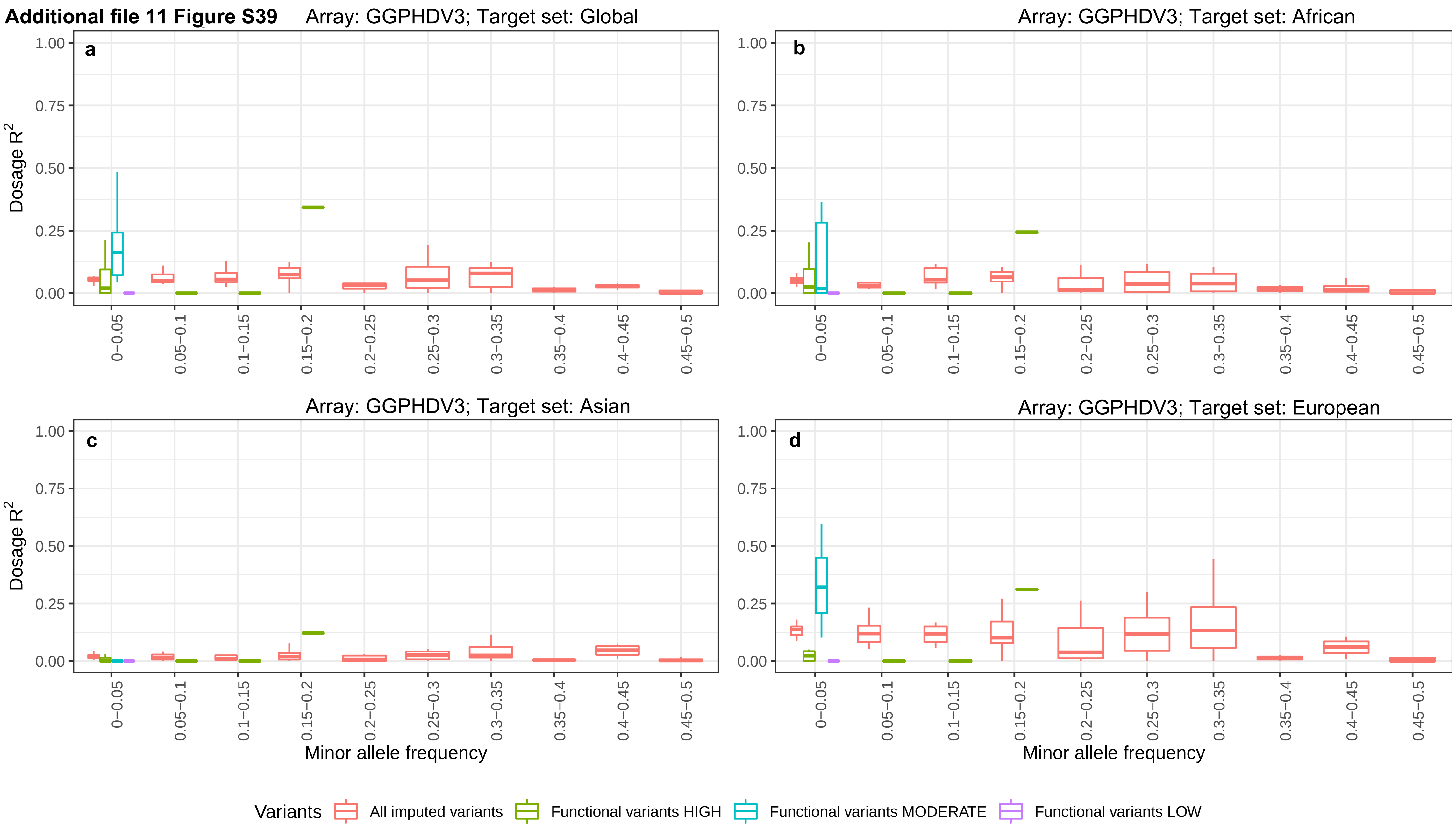

Additional file 11 Figure S40

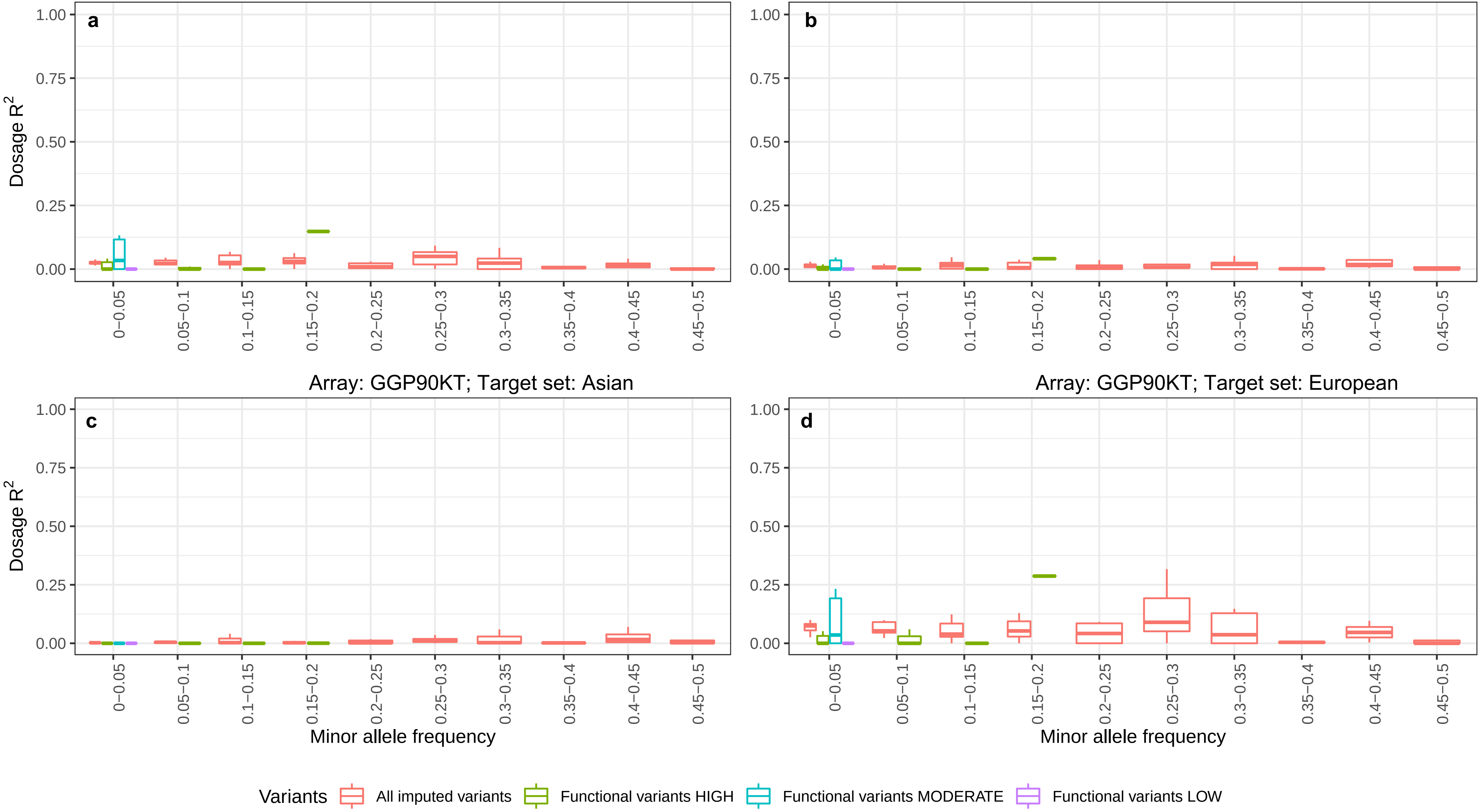

Additional file 11 Figure S41

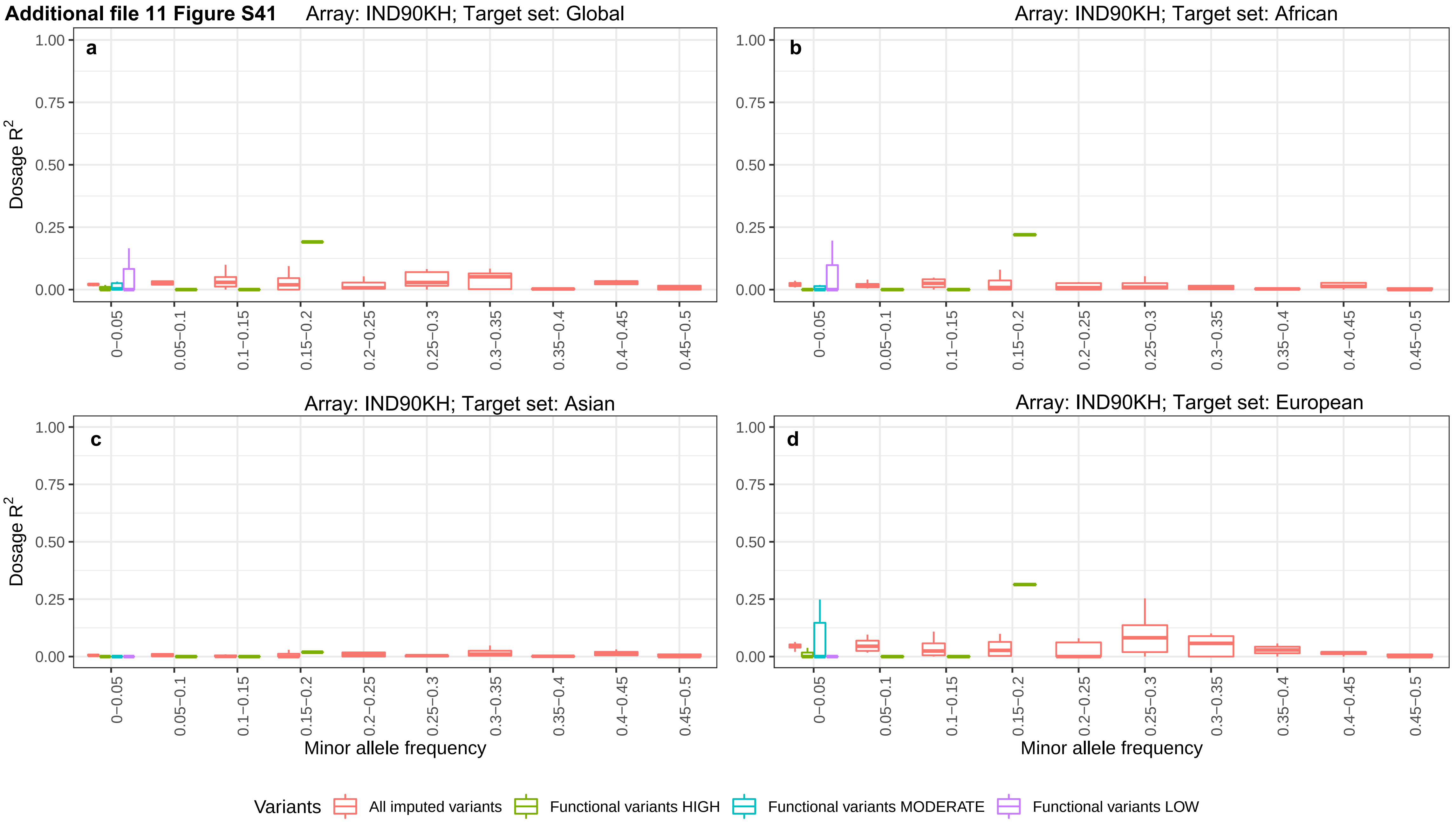

Additional file 11 Figure S42

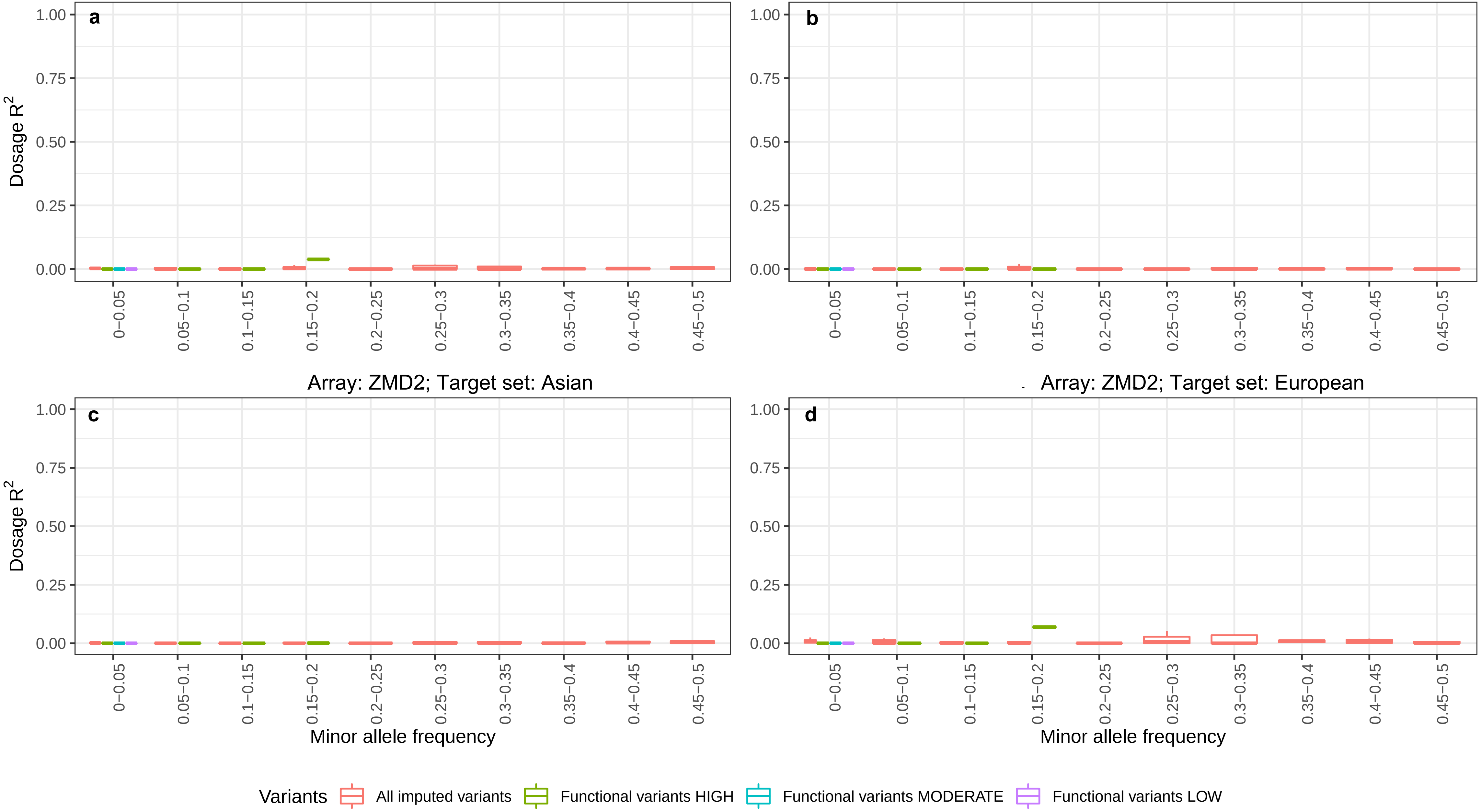

Additional file 11 Figure S43

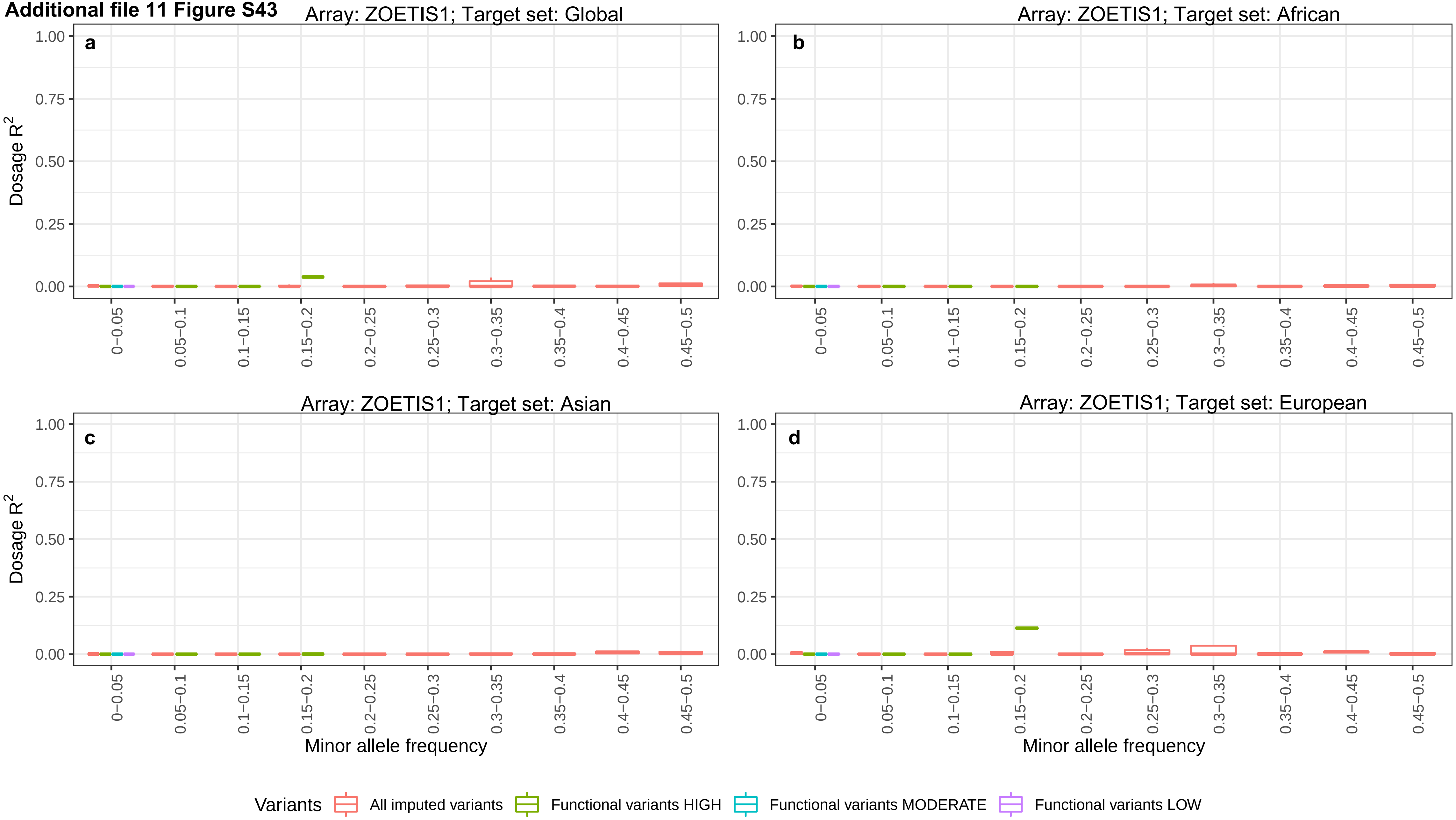

Additional file 11 Figure S44

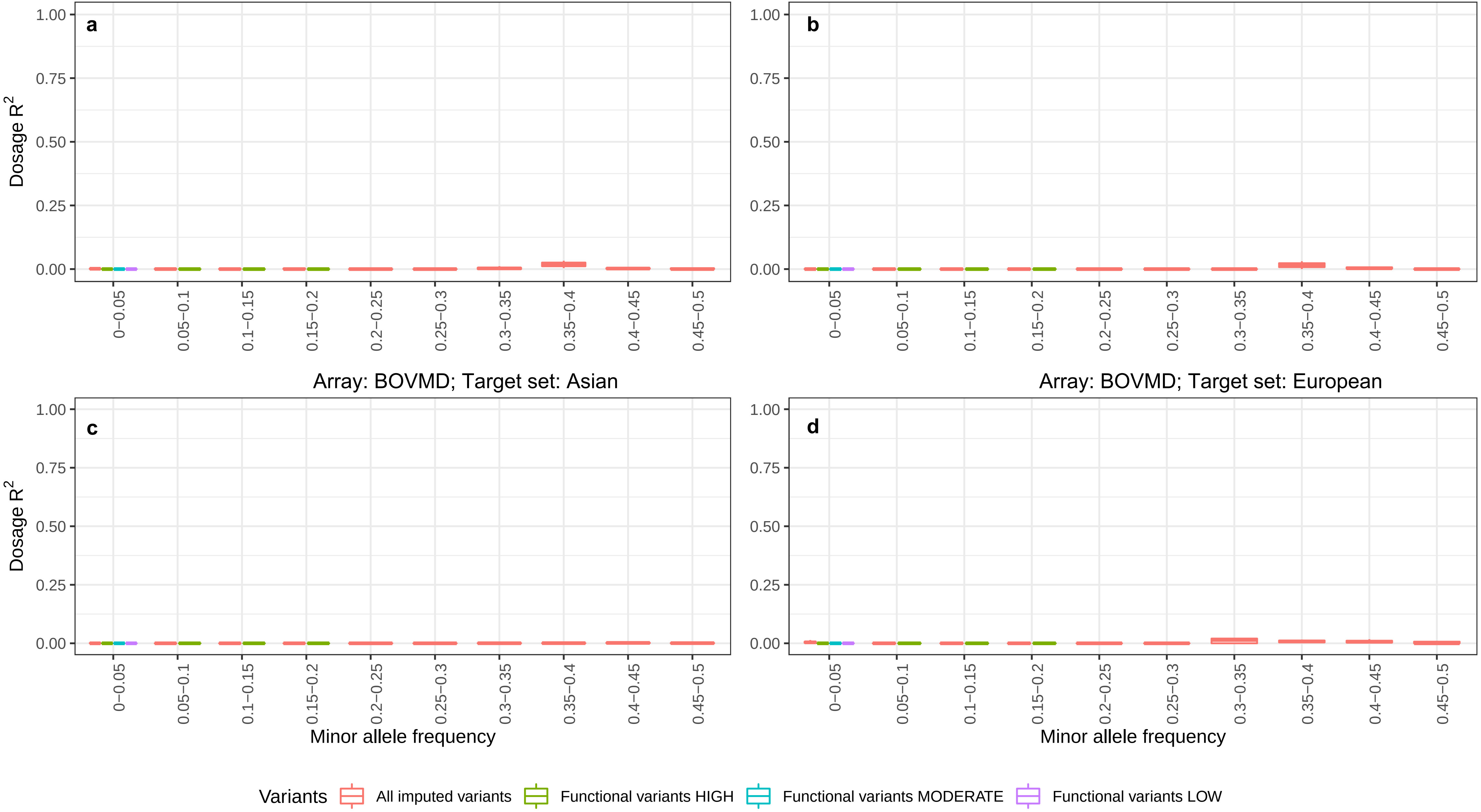

Additional file 11 Figure S45

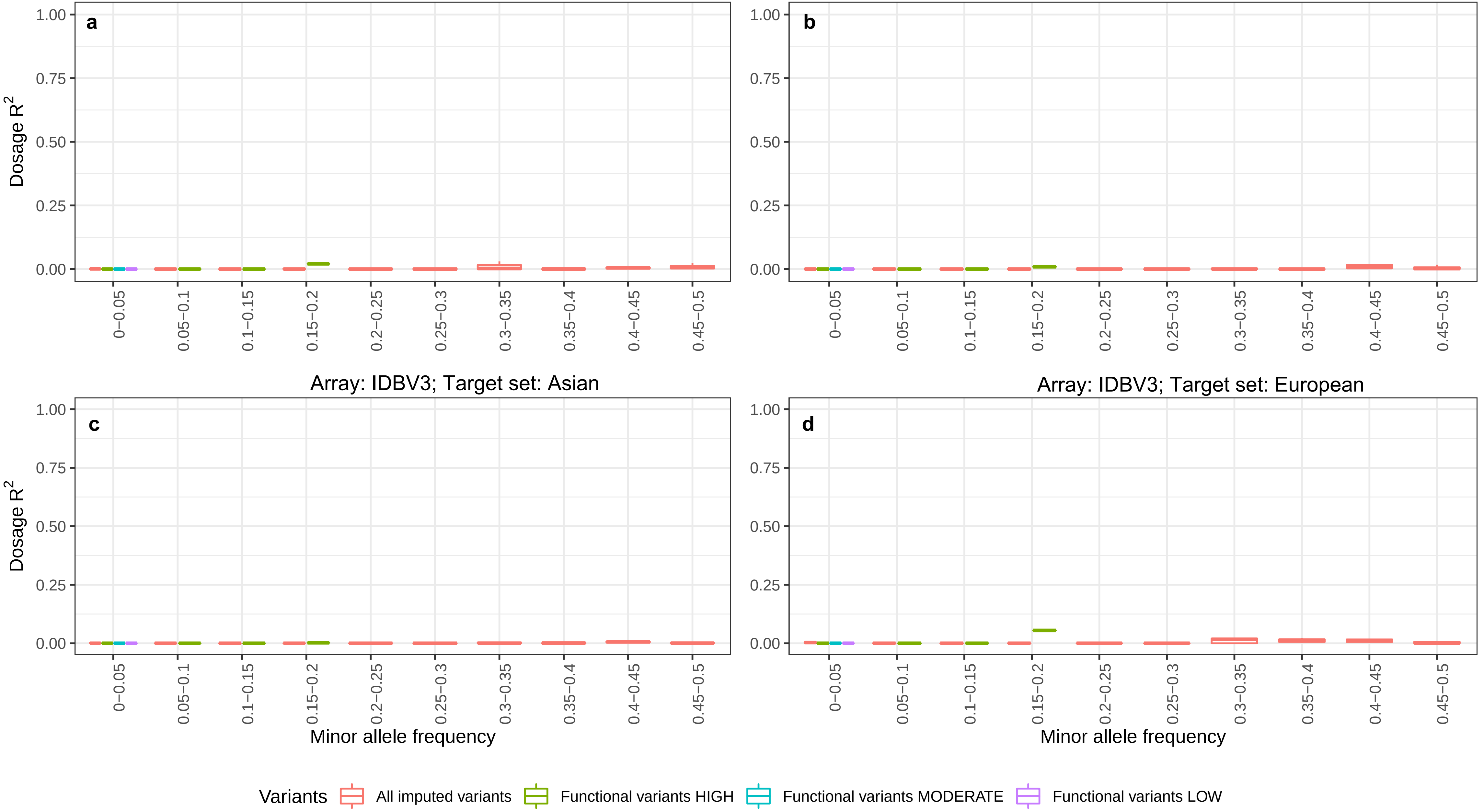

Additional file 11 Figure S46

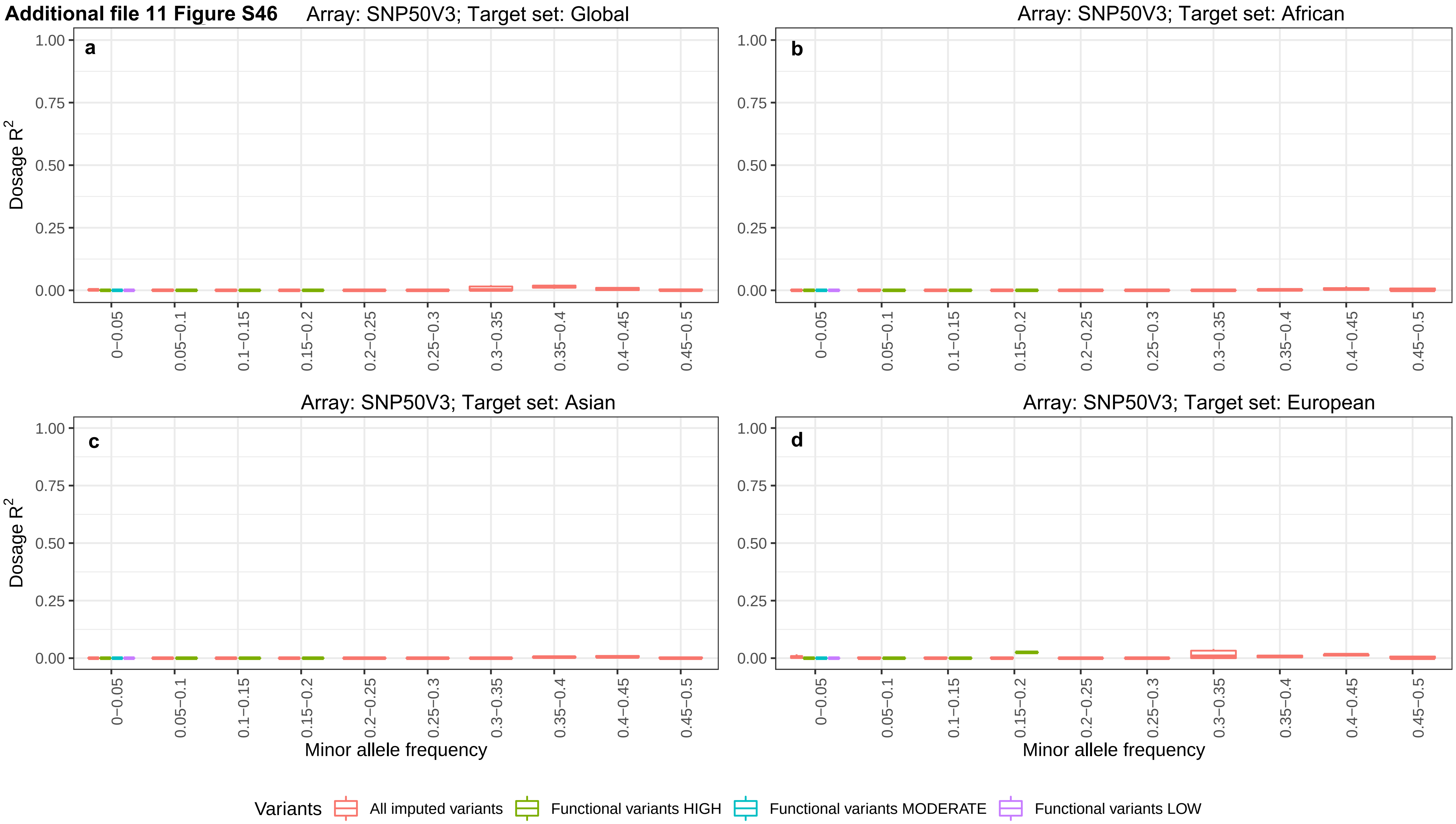

Additional file 11 Figure S47

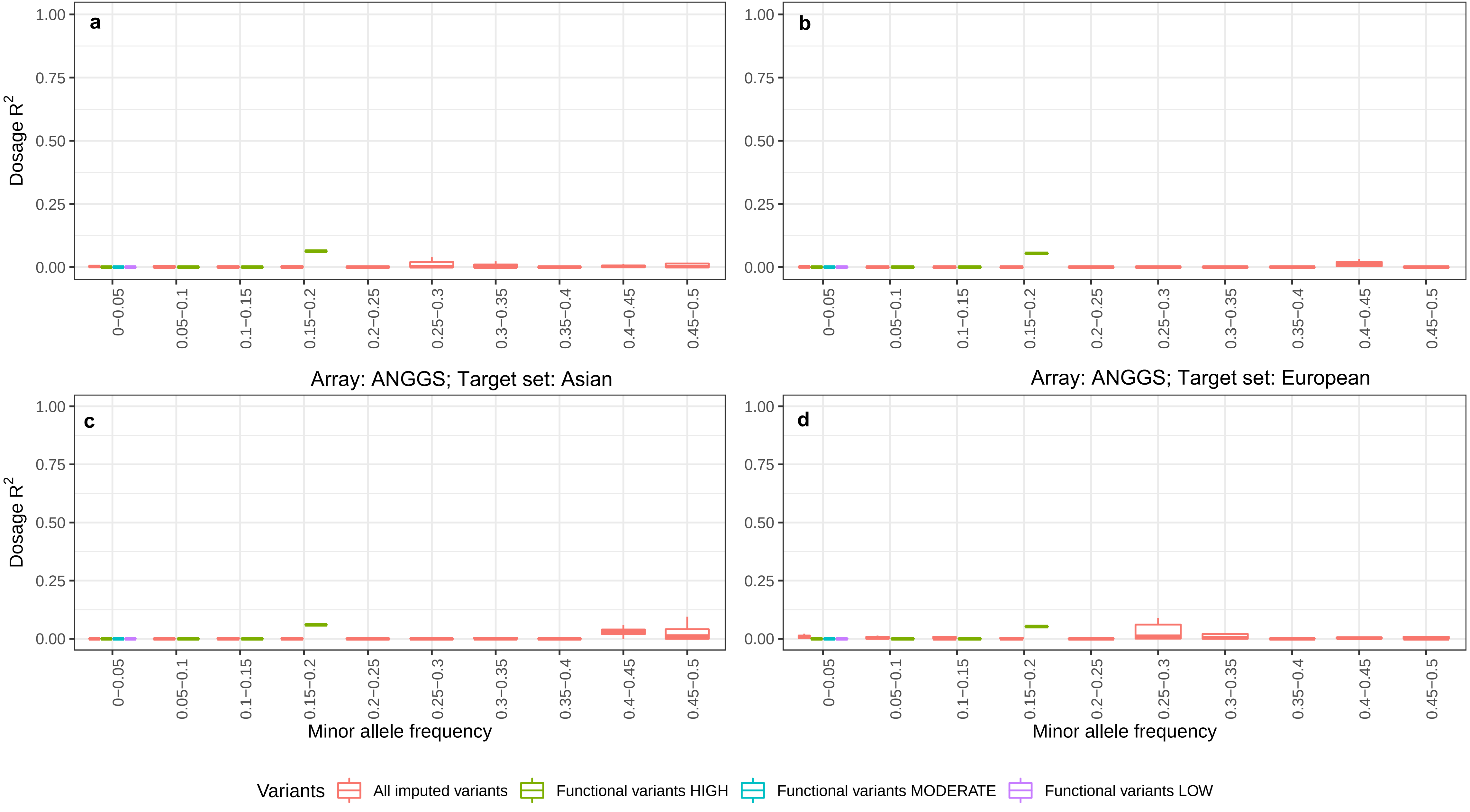

Additional file 11 Figure S48

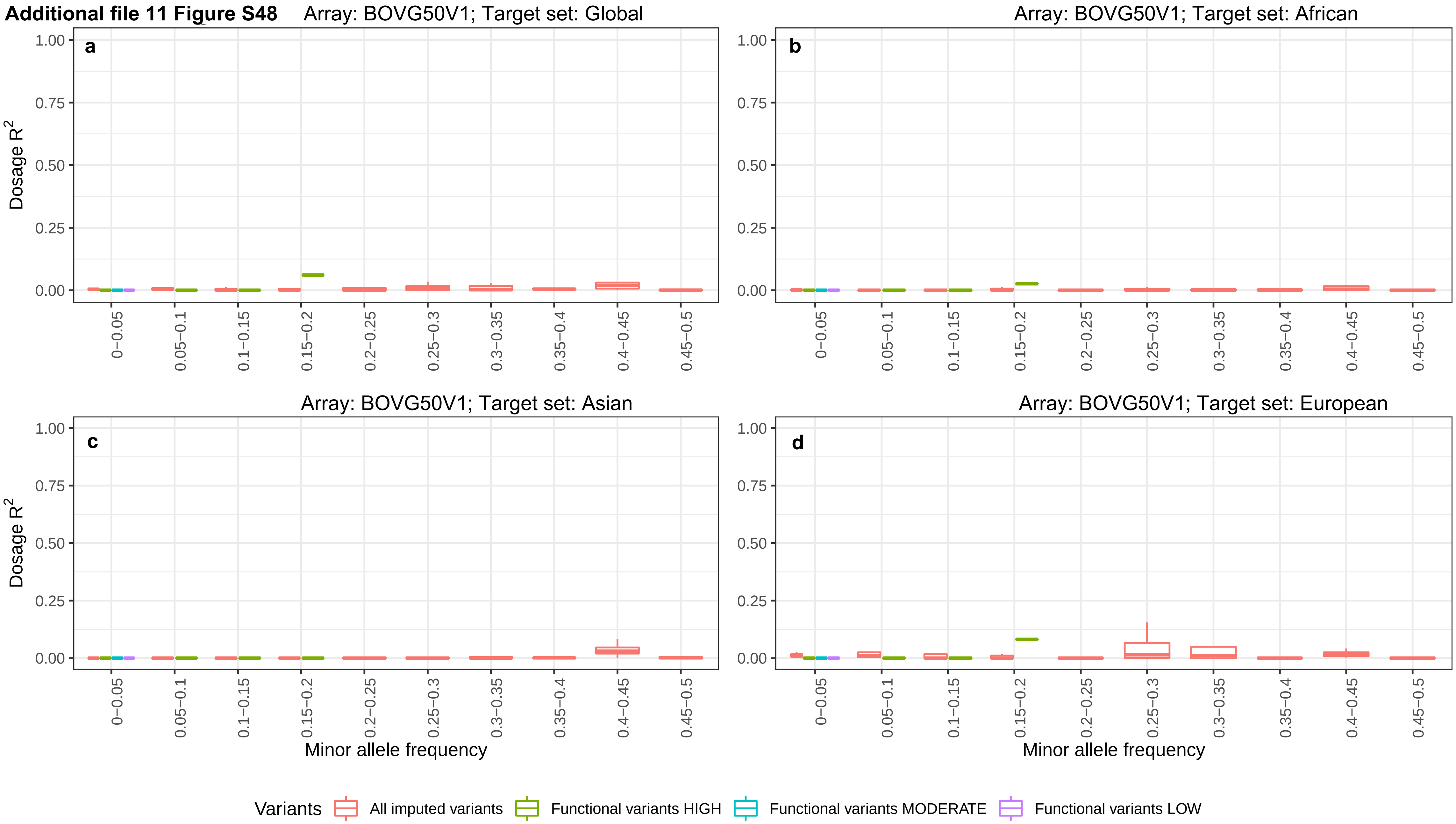

Additional file 11 Figure S49

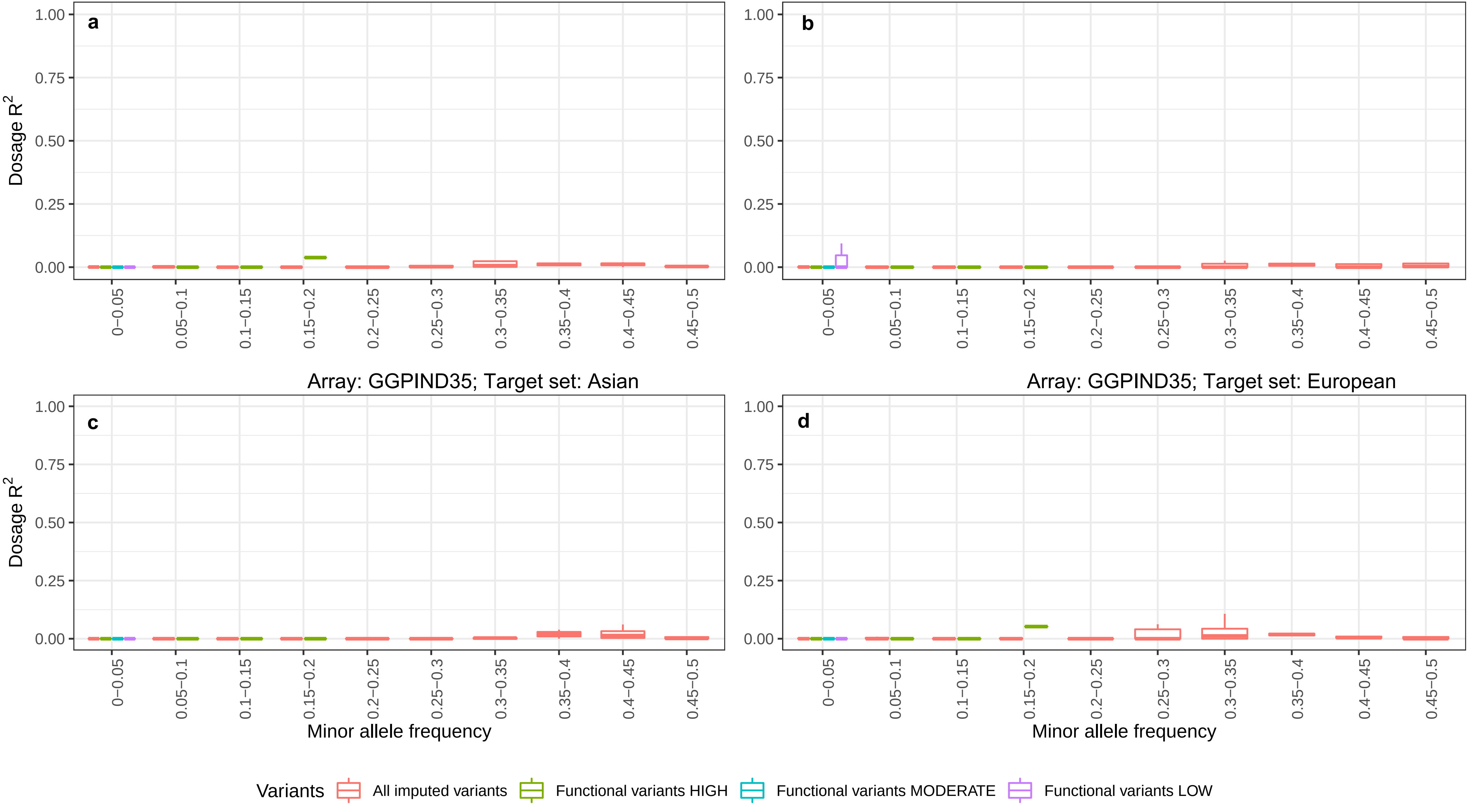

Additional file 11 Figure S50

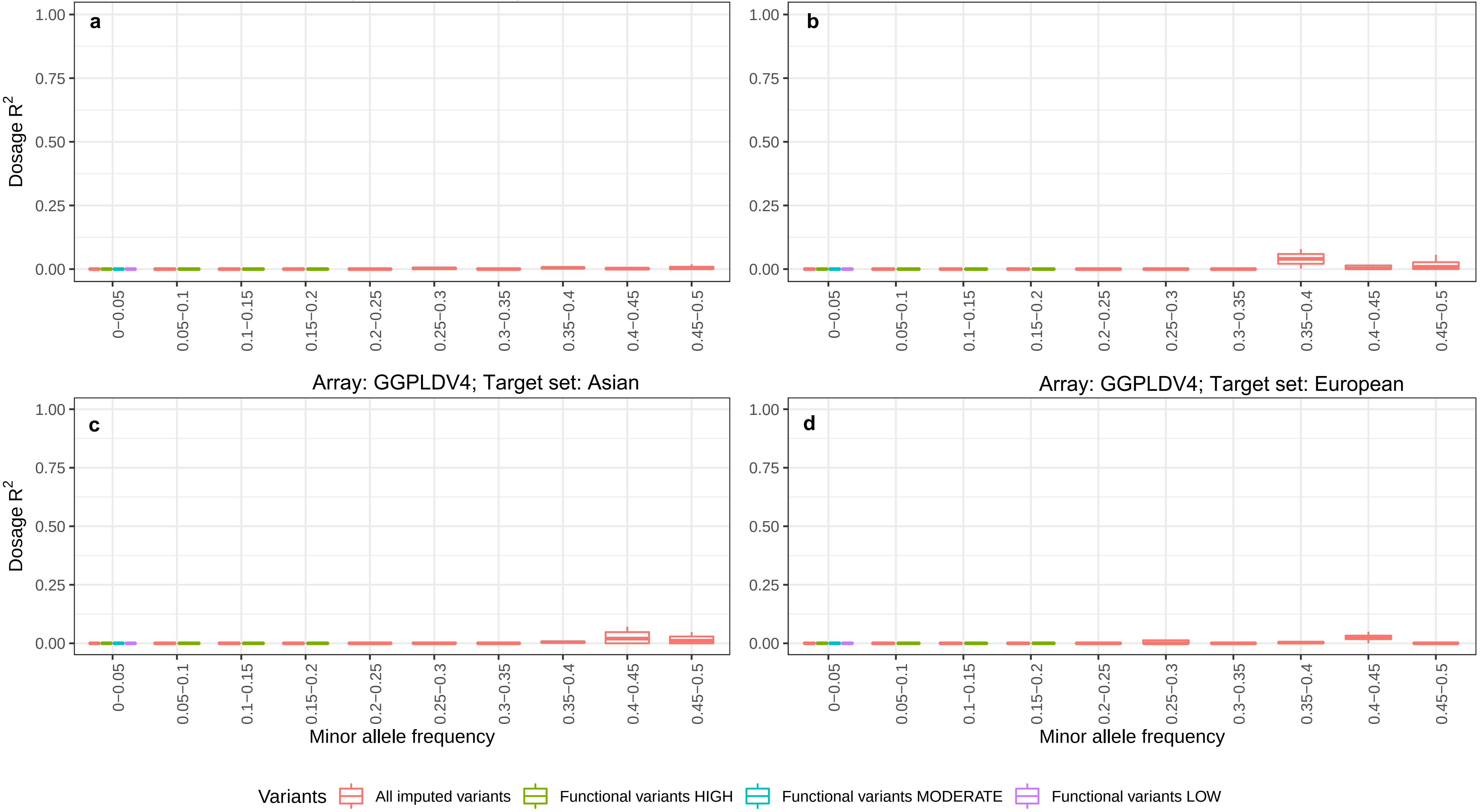

Additional file 11 Figure S51

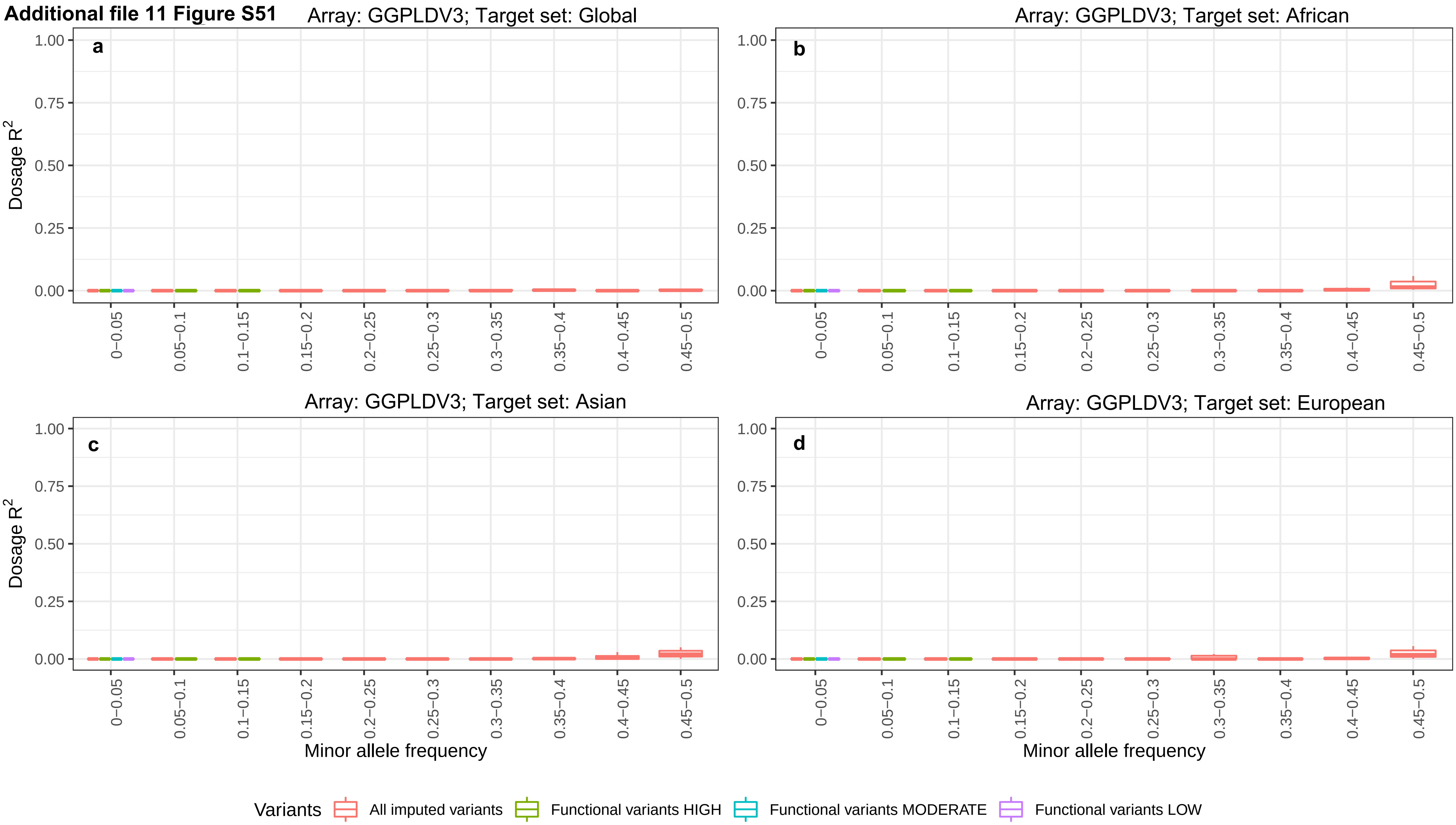

Supplement: Supplementary file 11 — Additional file 11: Figure S36. Dosage R2 for all imputed indels and for functional indels for the HD array when using the Global Reference Panel and four target sets (i.e., Global, African, Asian and European). Dosage R2 for all imputed variants and for functional indels (as annotated by the Ensembl VEP software (LOW, MODERATE and HIGH)) when imputing from the HD array to WGS level. The target sets were created by retaining only the WGS genotypes that overlapped with the variants of the HD array, from the Global Reference Panel as well as its subsets, generated according to the continent of origin (African (87 individuals), Asian (106 individuals) and European (77 individuals) subsets). These target sets (i.e. Global, African, Asian, and European) were then used to impute to WGS level using the Global Reference Panel. Figure S37. Dosage R2 for all imputed indels and for functional indels for the BOS1 array when using the Global Reference Panel and four target sets (i.e., Global, African, Asian and European). Dosage R2 for all imputed variants and for functional indels (as annotated by the Ensembl VEP software (LOW, MODERATE and HIGH)) when imputing from the BOS1 array to WGS level. The target sets were created by retaining only the WGS genotypes that overlapped with the variants of the BOS1 array, from the Global Reference Panel as well as its subsets, generated according to the continent of origin (African (87 individuals), Asian (106 individuals) and European (77 individuals) subsets). These target sets (i.e. Global, African, Asian, and European) were then used to impute to WGS level using the Global Reference Panel. Figure S38. Dosage R2 for all imputed indels and for functional indels for the GGPF250 array when using the Global Reference Panel and four target sets (i.e., Global, African, Asian and European). Dosage R2 for all imputed variants and for functional indels (as annotated by the Ensembl VEP software (LOW, MODERATE and HIGH)) when imputing from theGGPF [file 12711_2022_751_MOESM11_ESM.pdf]
